# Proinflammatory and Hyperinsulinemic Dietary Patterns Are Associated With Specific Profiles of Biomarkers Predictive of Chronic Inflammation, Glucose-Insulin Dysregulation, and Dyslipidemia in Postmenopausal Women

**DOI:** 10.3389/fnut.2021.690428

**Published:** 2021-09-20

**Authors:** Ni Shi, Desmond Aroke, Qi Jin, Dong Hoon Lee, Hisham Hussan, Xuehong Zhang, JoAnn E. Manson, Erin S. LeBlanc, Ana Barac, Chrisa Arcan, Steven K. Clinton, Edward L. Giovannucci, Fred K. Tabung

**Affiliations:** ^1^Comprehensive Cancer Center, The Ohio State University, Columbus, OH, United States; ^2^Division of Medical Oncology, Department of Internal Medicine, The Ohio State University, Columbus, OH, United States; ^3^Department of Medicine, Rutgers Health, Newark Beth Israel Medical Center, Newark, NJ, United States; ^4^Interdisciplinary Ph.D. Program in Nutrition, The Ohio State University, Columbus, OH, United States; ^5^Department of Nutrition, Harvard T. H. Chan School of Public Health, Boston, MA, United States; ^6^Division of Gastroenterology, Hepatology and Nutrition, Department of Internal Medicine, The Ohio State University, Columbus, OH, United States; ^7^Department of Medicine, Brigham and Women's Hospital, Harvard, Medical School, Boston, MA, United States; ^8^Department of Epidemiology, Harvard T. H. Chan School of Public Health, Boston, MA, United States; ^9^Kaiser Permanente Center for Health Research NW, Portland, OR, United States; ^10^Medstar Heart and Vascular Institute, Georgetown University, Washington, DC, United States; ^11^Department of Family, Population, and Preventive Medicine, Nutrition Division, Renaissance School of Medicine, Stony Brook University, Stony Brook, NY, United States; ^12^Division of Epidemiology, College of Public Health, The Ohio State University, Columbus, OH, United States

**Keywords:** hyperinsulinemic dietary pattern, proinflammatory dietary pattern, circulating biomarkers, inflammation, insulin response/IGF system, lipids

## Abstract

**Background:** Dietary patterns promoting hyperinsulinemia and chronic inflammation, including the empirical dietary index for hyperinsulinemia (EDIH) and empirical dietary inflammatory pattern (EDIP), have been shown to strongly influence risk of weight gain, type 2 diabetes, cardiovascular disease, and cancer. EDIH was developed using plasma C-peptide, whereas EDIP was based on plasma C-reactive protein (CRP), interleukin-6, and tumor necrosis factor alpha receptor 2 (TNF-αR2). We investigated whether these dietary patterns were associated with a broader range of relevant biomarkers not previously tested.

**Methods:** In this cross-sectional study, we included 35,360 women aged 50–79 years from the Women's Health Initiative with baseline (1993–1998) fasting blood samples. We calculated EDIH and EDIP scores from baseline food frequency questionnaire data and tested their associations with 40 circulating biomarkers of insulin response/insulin-like growth factor (IGF) system, chronic systemic inflammation, endothelial dysfunction, lipids, and lipid particle size. Multivariable-adjusted linear regression was used to estimate the percent difference in biomarker concentrations per 1 standard deviation increment in dietary index. FDR-adjusted *p* < 0.05 was considered statistically significant.

**Results:** Empirical dietary index for hyperinsulinemia (EDIH) and empirical dietary inflammatory pattern (EDIP) were significantly associated with altered concentrations of 25 of the 40 biomarkers examined. For EDIH, the percent change in biomarker concentration in the insulin-related biomarkers ranged from +1.3% (glucose) to +8% (homeostatic model assessment for insulin resistance) and −9.7% for IGF-binding protein-1. EDIH impacted inflammation and endothelial dysfunction biomarkers from +1.1% (TNF-αR2) to +7.8% (CRP) and reduced adiponectin by 2.4%; and for lipid biomarkers: +0.3% (total cholesterol) to +3% (triglycerides/total cholesterol ratio) while reducing high-density lipoprotein cholesterol by 2.4%. EDIP showed a similar trend of associations with most biomarkers, although the magnitude of association was slightly weaker for the insulin-related biomarkers and stronger for lipids and lipid particle size.

**Conclusions:** Dietary patterns with high potential to contribute to insulin hypersecretion and to chronic systemic inflammation, based on higher EDIH and EDIP scores, were associated with an unfavorable profile of circulating biomarkers of glucose-insulin dysregulation, chronic systemic inflammation, endothelial dysfunction and dyslipidemia. The broad range of biomarkers further validates EDIH and EDIP as mechanisms-based dietary patterns for use in clinical and population-based studies of metabolic and inflammatory diseases.

## Introduction

The dietary pattern approach has been widely integrated into nutritional epidemiology as a more comprehensive approach to understanding the relationship between whole diets and disease risk and prognosis (1). Two major strategies for defining dietary patterns are the *a priori* or hypothesis-oriented approach and the *a posteriori* or empirical approach. The hypothesis-oriented approach uses prevailing evidence regarding a diet-disease relation to define a pattern or dietary guidelines, such as the Dietary Guidelines for Americans assessed using the healthy eating index. In contrast, the *a posteriori* strategy employs statistical approaches to group dietary variables into patterns in a purely empirical manner (2). These strategies do not account for the potential of the diet to influence biomarkers of biological processes, such as insulin response or systemic inflammation, which are important in the pathogenesis of several chronic diseases.

Systemic inflammation and hyperinsulinemia are associated with the development of several chronic diseases, including type 2 diabetes, cardiovascular disease, and cancer (3, 4). Our team previously utilized a hybrid approach to define empirical hypothesis-oriented dietary patterns that are data driven, yet based on a specific hypothesis (e.g., hyperinsulinemia, chronic systemic inflammation) relating diet with disease. Two dietary indices were developed and validated: empirical dietary index for the hyperinsulinemia (EDIH) score and the empirical dietary inflammatory pattern (EDIP) score for systemic inflammation (5). Both indices were developed by weighting how foods consumed predicted concentrations of relevant biomarkers, C-peptide as a marker of insulin resistance and β-cell secretory activity for EDIH and inflammatory markers CRP, IL-6, and TNFa-R2 for EDIP (5). Although previous studies evaluating EDIH and EDIP reported significant associations with chronic diseases, including obesity (6), type 2 diabetes (7, 8), cardiovascular disease (9), and cancers (10–17), it is unclear if these dietary patterns predict other relevant biomarkers of metabolic disease beyond the few biomarkers involved in the original development of the indices.

Inflammation and hyperinsulinemia are conceptually distinct but interrelated phenomena, particularly as both are strongly associated with obesity, and each process acts through numerous and interacting pathways. Multiple biomarkers in these pathways have been investigated in various clinical and epidemiologic studies as objective measures of inflammation and insulinemia and relationships to downstream clinical disease processes. The EDIH and EDIP scores were developed and validated using a limited set of biomarkers. If the EDIH and EDIP broadly reflect the ability of respective dietary patterns to impact insulinemia and inflammation, we hypothesize that EDIH and EDIP scores will be linked to concentrations of a broader range of mediators and biomarkers related to these pathways. In addition, given the cross talk between inflammation and hyperinsulinemia, as well as the moderate statistical correlation between the EDIH and EDIP (Spearman *r* = 0.5–0.7), it is important to test the extent to which each dietary index predicts biomarkers representing the construct of the other dietary index.

In the current study, our objective was to examine a large cohort within the Women's Health Initiative and define associations of EDIH and EDIP dietary patterns with concentrations of a comprehensive panel of 40 circulating biomarkers of insulin response/IGF signaling, chronic systemic inflammation, endothelial dysfunction, and dyslipidemia. In secondary analyses, we examined the association of the dietary indices with type 2 diabetes and further explored associations in subgroups of potential modifying factors.

## Materials and Methods

### Study Population

About 161,808 postmenopausal women aged 50–79 years were enrolled in the Women's Health Initiative (WHI) in 40 clinical sites in the United States between 1993 and 1998 (18, 19). The full WHI consisted of four clinical trials (CT): dietary modification trial (DMT), calcium and Vitamin D trial, two hormone therapies—estrogen alone and estrogen plus progestin trials; and an observational study (OS). The WHI-OS was composed of 93,676 women who were not eligible or unwilling to participate in the CT (18). Trained study nurses drew blood samples and measured blood pressure, height, and weight at the baseline clinic visit.

**Figure 1:**
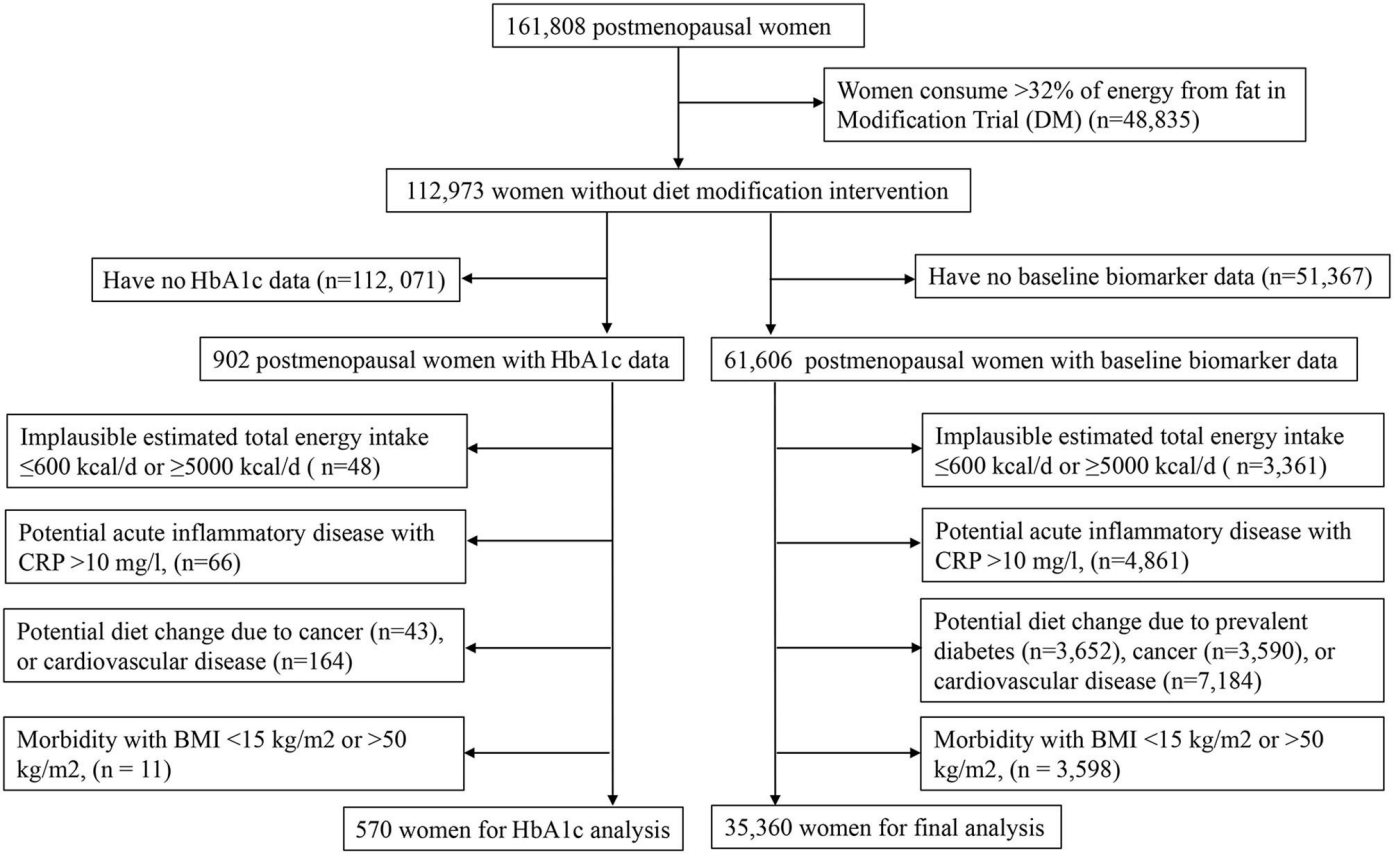
A flow chart for eligible participants enrolled in the Women's Health Initiative.

In this cross-sectional study, we considered 61,606 women for inclusion from OS and three CT except DMT, who provided baseline blood sample data on circulating biomarkers of insulin response/IGF system, inflammation, endothelial dysfunction, and dyslipidemia. After the exclusions described in Figure 1 were applied, a total of 35,360 women were retained for final analysis. The final analytic sample and the excluded sample were comparable in major demographic and lifestyle characteristics (Supplementary Table 1). The WHI protocol was approved by the institutional review boards at the Clinical Coordinating Center at the Fred Hutchinson Cancer Research Center in Seattle, WA, and at each of the 40 clinical centers (18).

### Dietary Assessment and Calculation of Dietary Indices

Baseline habitual diet was estimated from the WHI baseline semiquantitative food frequency questionnaire (FFQ) (20, 21). The WHI FFQ contained 122-line items, covering more than 300 foods and assessed dietary intake in the preceding 3-month period. The WHI FFQ was previously evaluated for validity against intakes from four 24-h dietary recall interviews and 4 days of food diaries recorded within the WHI. Intakes of 31 nutrients were found to be quite comparable across the dietary assessment instruments. Foods (servings per day) and nutrient intakes were then estimated using the Nutrition Data System for Research (NDSR) database version 2005 (22) of the University of Minnesota.

We developed and validated the EDIP and EDIH scores in the Nurses' Health Study (NHS) to empirically assess the insulinemic (EDIH) or inflammatory (EDIP) potential of dietary patterns based upon specific combinations of foods/food groups (5). Briefly, the EDIH is comprised of 18 food groups most predictive of plasma C-peptide concentrations. The food groups contributing to high EDIH scores include cream soups, butter, red meat, low-energy sugar-sweetened beverages (e.g., diet soda), high-energy sugar-sweetened beverages (e.g., regular soda), processed meat, poultry, margarine, French fries, non-dark fish, tomatoes, low-fat dairy, and eggs; whereas the food groups contributing to lower EDIH score include wine, whole fruit, coffee, full-fat dairy products and green leafy vegetables. More positive EDIH scores indicate a hyperinsulinemic dietary pattern (5). EDIP, developed in a separate study, is a weighted sum of 18 food groups most predictive of three plasma inflammatory biomarkers (IL6, CRP, and TNFαR-2). Food groups that contributed to higher EDIP scores are the following: red meat, organ meat, processed meat, non-dark fish, refined grains, other vegetables (i.e., vegetables other than green leafy vegetables and dark-yellow vegetables), low-energy sugar-sweetened beverages, high-energy sugar-sweetened beverages, and tomatoes; whereas the foods contributing to lower EDIP scores include tea, coffee, beer, wine, dark-yellow vegetables, green leafy vegetables, snacks, fruit juice, and pizza. Higher EDIP scores reflect more proinflammatory dietary patterns (5). We calculated both scores for each participant using the baseline WHI FFQ food data, and the food components comprising each food group in the WHI as listed in Supplementary Table 2.

### Biomarker Assessment

We obtained biomarker data assessed at baseline in several case-control studies nested within the WHI (Supplementary Table 3). These included insulin-related and insulin-like growth factors (IGF) and binding proteins (BP): glucose, insulin, C-peptide, homeostasis model assessment of insulin resistance, [HOMA-IR = insulin (μIU/ml) × glucose (mmol/L)/22.5], homeostasis model assessment of beta-cell function, [HOMA-β = (20 × insulin (μIU/ml)/(glucose (mmol/L)-3.5] (23), glycated hemoglobin (HbA1c), IGF-1, free IGF-1, IGFBP-1, IGFBP-3, and IGFBP-4 (24). Inflammatory biomarkers included C-reactive protein (CRP), serum amyloid A (SAA), interleukins (IL-6, IL-10), tumor necrosis factor (TNF)-alpha, TNF-alpha receptor 1 (TNF-αR1), TNF-αR2, adiponectin and leptin (25). Endothelial dysfunction biomarkers included vascular endothelial growth factor (VEGF), endothelial leukocyte adhesion molecule, E-selectin, vascular cell adhesion molecule 1 (VCAM-1), intercellular adhesion molecule 1 (ICAM-1), granulocyte colony stimulating factor (GCSF) (25). Blood lipid biomarkers including total cholesterol (TC), triglycerides (TG), high-density lipoprotein cholesterol (HDL), low-density lipoprotein cholesterol (LDL), the ratio of TG/HDL and the ratio of TG/TC (26). Lipid particles biomarkers included large LDL particles, medium LDL particles, small LDL particles, very small LDL particles, total size of all LDL particles, intermediate density lipoprotein (IDL) particles, large HDL particles, medium HDL particles, small HDL particles, and total size of all HDL particles (26). Biomarker assessment details are described in Supplementary Table 3.

### Statistical Analysis

To describe characteristics of participants, all categorical variables were presented using frequencies (%), and all continuous variables were presented using means (standard deviations) across quintiles of the dietary indices (EDIH and EDIP). Biomarker data were normalized *via* log transformation using natural logs. We estimated the relative change (as a percent) in biomarker concentration per 1 standard deviation increase in the dietary score using multivariable linear regression analyses with the continuous dietary index score, adjusting for all the covariates listed below. In addition, we estimated the absolute concentrations of biomarkers in quintiles of EDIH and EDIP *via* back transformation of the log-transformed values. We also calculated the percentage difference in biomarker concentration between the highest and lowest dietary index quintiles. A false discovery rate (FDR)-adjusted *p* < 0.05 was considered statistically significant.

In a subsample analysis among 902 women with data on glycated hemoglobin (HbA1c) and fasting blood glucose, we examined associations of the dietary indices with prediabetes and type 2 diabetes defined based on the American Diabetes Association (ADA) criteria: prediabetes—HbA1c 5.7–6.4% or impaired fasting glucose (IFG) 100 mg/dL to 125 mg/dL; and type 2 diabetes—HbA1c ≥ 6.5% and IFG ≥ 126 mg/dL (27). We applied the same exclusion criteria as for the main analysis except the exclusion of women with prevalent diabetes at baseline and retained 570 women for the subsample analysis. We used multivariable-adjusted logistic regression to estimate odds of prediabetes, diabetes, and prediabetes and diabetes combined.

The following covariates were assessed and included in the multivariable-adjusted models: total energy intake (kcal/day, continuous); age at the WHI baseline (years, continuous); body mass index [BMI = weight (kg)/(height (m)^2^, continuous]; total recreational physical activity (MET-hours/week, calculated as a continuous variable by summing the metabolic equivalent tasks for all reported activities for each individual, such as walking, aerobics, jogging, tennis, swimming, biking outdoors, exercise machine, calisthenics, popular or folk dancing); pack-years of smoking (continuous); number of nutrient supplements used (continuous); fasting status at blood draw (<8 h or ≥8 h); the self-reported racial/ethnic group [American Indian or Alaska Native, Asian or Pacific Islander, Black or African-American, Hispanic/Latino, White (not of Hispanic origin), and others]; educational levels (some high school or lower educational level, high school graduate or some college or associate degree, and ≥4 years of college); regular use of medications: aspirin and other non-steroidal anti-inflammatory drugs (NSAIDs) (yes/no), unopposed estrogen and/or estrogen plus progesterone (yes/no), and statins (yes/no) (regular use was defined as ≥2 times in each of the 2 weeks preceding the interview); hormone therapy (HT) study arms (not randomized to HT, estrogen-alone intervention, estrogen-alone control, estrogen-plus-progestin intervention, estrogen-plus progestin control). Data on these covariates were collected by self-administered questionnaires on demographics, medical history, and lifestyle factors at the baseline (18, 19).

In exploratory analyses, we performed six subgroup analyses in categories of potential effect modifiers that included BMI (normal weight −18.5 to <25, overweight −25 to <30, obese −30 to 50 kg/m2); waist-to-hip ratio (high WHR > 0.85; low WHR ≤ 0.85); the race/ethnic group (non-Hispanic White, Black/African American, Hispanic/Latino); NSAIDs (regular user/non-user); unopposed estrogen (never user, past user, and current user) and statin use (regular user /non-user). Interaction was assessed using the Wald *p* value of the interaction term. Significant interaction and associations within the subgroup were assessed at an FDR-adjusted *p* < 0.10. All analyses were conducted using SAS® version 9.4 (SAS Institute, Cary, NC).

## Results

### Characteristics of Participants

Table 1 shows the baseline characteristics of participants in quintiles of EDIH and EDIP dietary patterns. Women consuming the most hyperinsulinemic or proinflammatory dietary patterns (quintile 5 of EDIH or EDIP had higher proportions of Black or African-American and Hispanic/Latino women, lower proportion of White women, higher BMI, lower physical activity, and higher proportion with a lower level of education, compared with those in quintile 1. In terms of food intake, women in the highest quintile of EDIH and EDIP had a higher intake of red/processed meat, sugar-sweetened beverages, and lower intakes of fruit and green-leafy vegetables compared with those in the lowest quintile. The macronutrient profile of women in the highest quintile of EDIH and EDIP was characterized by higher intakes of total and saturated fat and lower total carbohydrate, coupled with lower total fiber, compared with those in the lowest quintiles.

**Table 1:** Baseline characteristics of the study sample in quintiles of dietary indices among 35,360 postmenopausal women in Women's Health Initiative.

	**Empirical Dietary Index for Hyperinsulinemia (EDIH) score quintiles** ^**a**^						**Empirical Dietary Inflammatory Index (EDIP) score quintiles**					
**Characteristic** ^**b**^	**Quintile 1** **(*n* = 7,072)**	**Quintile 2** **(*n* = 7,072)**	**Quintile 3** **(*n* = 7,072)**	**Quintile 4** **(*n* = 7,072)**	**Quintile 5** **(*n* = 7,072)**	***P* value** ^**c**^	**Quintile 1** **(*n* = 7,072)**	**Quintile 2** **(*n* = 7,072)**	**Quintile 3** **(*n* = 7,072)**	**Quintile 4** **(*n* = 7,072)**	**Quintile 5** **(*n* = 7,072)**	***P* value** ^**c**^
Race/ethnicity^d^, (%)												
Black or African American	595 (8.4)	710 (10.0)	906 (12.8)	1,166 (16.5)	1,649 (23.3)	<0.001	446 (6.3)	624 (8.8)	871 (12.3)	1,274 (18.0)	1,811 (25.6)	<0.001
American Indian or Alaskan Native	49 (0.7)	49 (0.7)	85 (1.2)	61 (0.9)	77 (1.1)		62 (0.9)	60 (0.9)	53 (0.8)	72 (1.0)	74 (1.1)	
Hispanic/Latino	349 (4.9)	400 (5.7)	446 (6.3)	524 (7.4)	605 (8.6)		232 (3.3)	274 (3.9)	367 (5.2)	518 (7.3)	933 (13.2)	
Asian or Pacific islander	175 (2.5)	249 (3.5)	259 (3.7)	259 (3.7)	184 (2.6)		104 (1.5)	165 (2.3)	191 (2.7)	286 (4.0)	380 (5.4)	
White (not of Hispanic origin)	5,836 (82.5)	5,605 (79.3)	5,318 (75.2)	5,007 (70.8)	4,494 (63.6)		6,170 (87.2)	5,896 (83.4)	5,527 (78.1)	4,852 (68.6)	3,815 (54.0)	
Other	68 (1.0)	59 (0.8)	58 (0.8)	55 (0.8)	63 (0.9)		58 (0.8)	53 (0.7)	63 (0.9)	70 (1.0)	59 (0.8)	
Age, years	64.7 ± 7.2	64.9 ± 7.3	65.1 ± 7.2	64.4 ± 7.1	62.9 ± 7.3	<0.001	64.4 ± 7.1	64.8 ± 7.2	65.0 ± 7.2	64.5 ± 7.3	63.1 ± 7.4	<0.001
Body mass index (BMI), kg/m^2^, (%)	25.8 ± 5.0	26.2 ± 5.2	26.6 ± 5.3	27.4 ± 5.7	28.8 ± 6.3	<0.001	26.1 ± 5.2	26.4 ± 5.3	26.7 ± 5.5	27.2 ± 5.6	28.3 ± 6.3	<0.001
Underweight (15 ≤ BMI <18.4)	305 (4.3)	280 (4.0)	324 (4.6)	335 (4.7)	319 (4.5)	<0.001	301 (4.3)	322 (4.6)	324 (4.6)	310 (4.4)	306 (4.3)	<0.001
Normal weight (18.5 ≤ BMI <25)	3,144 (44.5)	2,991 (42.3)	2,601 (36.8)	2,213 (31.3)	1,682 (23.8)		2,937 (41.5)	2,797 (39.6)	2,643 (37.4)	2,322 (32.8)	1,932 (27.3)	
Overweight (25 ≤ BMI <30)	2,380 (33.7)	2,377 (33.6)	2,546 (36.0)	2,526 (35.7)	2,285 (32.3)		2,457 (34.8)	2,408 (34.0)	2,420 (34.2)	2,467 (34.9)	2,362 (33.4)	
Obese (BMI ≥ 30)	1,243 (17.6)	1,424 (20.1)	1,601 (22.6)	1,998 (28.3)	2,786 (39.4)		1,377 (19.5)	1,545 (21.8)	1,685 (23.8)	1,973 (27.9)	2,472 (35.0)	
Physical activity, MET^f^-hours/week	16.8 ± 15.7	14.7 ± 14.3	12.8 ± 13.2	11.0 ± 12.4	9.0 ± 11.4	<0.001	15.7 ± 15.3	14.2 ± 14.2	12.8 ± 13.3	11.5 ± 12.6	10.0 ± 12.5	<0.001
Pack-years of smoking	11.1 ± 18.9	9.7 ± 17.5	9.1 ± 17.2	9.0 ± 17.3	10.7 ± 19.6	<0.001	13.4 ± 20.8	10.8 ± 18.6	9.5 ± 17.4	8.4 ± 16.9	7.5 ± 16.1	<0.001
Current Smoking, (%)	491 (7.0)	458 (6.6)	507 (7.3)	584 (8.3)	830 (11.9)	<0.001	720 (10.)3	529 (7.6)	541 (7.7)	494 (7.1)	586 (8.4)	<0.001
Aspirin/NSAIDs^f^ use, (%)	987 (14.0)	983 (13.9)	1,004 (14.2)	941 (13.3)	956 (13.5)	0.5493	1,087 (15.4)	1,060 (15.0)	929 (13.1)	892 (12.6)	903 (12.8)	<0.001
Statin use, (%)	147 (2.1)	180 (2.6)	150 (2.1)	170 (2.4)	147 (2.1)	0.1994	130 (1.8)	146 (2.1)	176 (2.5)	174 (2.5)	168 (2.4)	0.0351
Educational level, (%)												
Less than high school	321 (4.5)	335 (4.7)	410 (5.8)	524 (7.4)	718 (10.2)	<0.001	250 (3.5)	310 (4.4)	367 (5.2)	498 (7.0)	883 (12.5)	<0.001
High school/GED^f^	3,516 (46.9)	3,644 (51.5)	3,972 (56.2)	4,193 (64.0)	4,380 (61.9)		3,675 (52.0)	3,732 (52.8)	3,935 (55.6)	4,062 (57.4)	4,612 (58.0)	
≥4 years of college	3,424 (48.4)	3,068 (43.4)	2,678 (37.9)	2,342 (33.1)	1,951 (27.6)		3,134 (44.3)	2,815 (42.7)	2,759 (39.0)	2,489 (35.2)	2,064 (29.2)	
Total alcohol intake, servings/week	4.7 ± 7.4	2.4 ± 4.3	1.8 ± 3.8	1.5 ± 3.5	1.5 ± 3.7	<0.001	5.1 ± 7.7	2.8 ± 4.4	1.9 ±3.7	1.3 ± 3.0	0.9 ± 2.6	<0.001
Food intake^e^, servings/week												
Red meat	2.1 ± 2.0	2.4 ± 2.2	2.9 ± 2.3	3.5 ± 2.6	5.6 ± 3.9	<0.001	3.0 ± 2.7	3.0 ± 2.6	3.0 ± 2.6	3.2 ± 2.8	4.2 ± 3.7	<0.001
Processed meat	1.1 ± 1.4	1.3 ± 1.5	1.5 ± 1.6	1.9 ± 1.9	3.1 ± 3.0	<0.001	1.5 ± 1.7	1.5 ± 1.7	1.6 ± 1.7	1.7 ± 1.9	2.5 ± 2.9	<0.001
Sugar-sweetened beverages	0.4 ± 1.4	0.5 ± 1.3	0.7 ± 1.7	1.1 ± 2.4	3.4 ± 6.4	<0.001	0.4 ± 1.4	0.6 ± 1.7	0.7 ± 1.9	1.1 ± 2.3	3.4 ± 6.3	<0.001
Refined grains	14.7 ± 8.7	12.8 ± 7.4	11.9 ± 6.9	11.4 ± 6.9	12.1 ± 7.3	<0.001	11.5 ± 6.8	11.6 ± 6.6	11.8 ± 6.8	12.5 ± 7.4	15.4 ± 9.2	<0.001
Wine	3.4 ± 5.8	1.3 ± 2.4	0.8 ± 1.7	0.5 ± 1.3	0.4 ± 1.2	<0.001	3.4 ± 5.8	1.5 ± 2.5	0.8 ± 1.6	0.5 ± 1.2	0.3 ± 0.8	<0.001
Tea/coffee	21.9 ± 14.8	16.1 ± 11.6	13.3 ± 10.6	11.5 ± 10.1	10.2 ± 10.0	<0.001	27.9 ± 15.5	17.3 ± 9.2	12.8 ± 7.7	9.2 ± 6.8	6.0 ± 6.1	<0.001
Whole fruit	25.0 ± 16.2	19.8 ± 11.2	17.0 ± 9.7	14.7 ± 9.0	13.4 ± 9.7	<0.001	20.3 ± 13.7	19.0 ± 11.9	17.9 ± 11.4	16.7 ± 11.1	15.9 ± 12.2	<0.001
Green-leafy vegetables	8.3 ± 6.6	6.5 ± 4.7	5.5 ± 4.2	4.8 ± 3.9	4.7 ± 4.0	<0.001	9.2 ± 6.9	6.8 ± 4.5	5.5 ± 3.9	4.6 ± 3.4	3.7 ± 3.3	<0.001
Nutrient profile												
Total fiber, g/d	19.2 ± 6.2	16.6 ± 5.7	14.7 ± 5.5	13.4 ± 5.5	13.2 ± 5.6	<0.001	17.3 ± 6.2	16.1 ± 6.0	15.2 ± 5.9	14.3 ± 5.9	14.2 ± 6.1	<0.001
Total carbohydrate, g/d	233.4 ± 66.7	200.0 ± 61.3	181.7 ± 60.4	171.7 ± 63.2	188.7 ± 70.9	<0.001	206.2 ± 67.6	194.6 ± 64.9	188.0 ± 64.7	183.9 ± 67.0	202.7 ± 72.9	<0.001
Total protein, g/d	69.0 ± 23.1	62.2 ± 22.7	59.2 ± 22.7	59.5 ± 23.3	70.3 ± 25.5	<0.001	68.2 ± 23.3	63.9 ± 22.8	61.5 ± 23.0	60.5 ± 23.6	66.1 ± 26.0	<0.001
Branched-chain amino acids, g/d	12.3 ± 4.3	11.1 ± 4.2	10.5 ± 4.1	10.6 ± 4.2	12.4 ± 4.6	<0.001	12.1 ± 4.3	11.3 ± 4.2	10.9 ± 4.2	10.8 ± 4.3	11.7 ± 4.7	<0.001
Total fat, g/d	54.8 ± 25.2	50.6 ± 24.0	50.9 ± 24.1	53.8 ± 24.3	69.5 ± 26.7	<0.001	55.7 ± 25.3	53.4 ± 24.5	52.9 ± 24.6	54.0 ± 25.2	63.6 ± 28.0	<0.001
Saturated fat, g/d	18.4 ± 9.2	16.8 ± 8.6	16.9 ± 8.5	17.8 ± 8.7	23.2 ± 9.6	<0.001	18.5 ± 9.2	17.8 ± 8.8	17.6 ± 8.8	18.0 ± 9.0	21.1 ± 10.0	<0.001

a *Dietary indices were adjusted for total energy intake*.

b *Values presented are mean ± SD for continuous variables and percentages for categorical variables*.

c *p value for differences in participant characteristics across quintiles, calculated using chi square test for categorical variables and ANOVA test for continuous variables*.

d *In WHI, race/ethnicity was self-identified. Users acknowledge that American Indian or Alaskan Native participants were self-identified and were primarily dwelling in urban areas and are not representative of the diverse American Indian population across the United States. Users agree not to use the data to infer tribal status or affiliation*.

e *The food group variables (servings/d) in the WHI were as follows: processed meat (hot dog, chorizo, other sausage, bacon, breakfast sausage, scrapple; lunch meat, such as ham, turkey; other lunch meat such as bologna); red meat (ground meat including hamburgers, beef, pork, and lamb as a main dish or as a sandwich; stew, pot pie, and casseroles with meat; gravy made with meat dripping); refined grains (total grain variable minus whole grain variable, both WHI-computed food groups); sugar-sweetened beverages, all regular (not diet) soft drinks and fruit juice; wine (red wine, white wine); coffee or tea (all types); green leafy vegetables (cooked greens, such as spinach, mustard greens, turnip greens, collards; lettuce and plain lettuce salad; mixed lettuce or spinach salad with vegetables)*.

f *MET, metabolic equivalent of task; NSAID, non-steroidal anti-inflammatory drug; GED, general educational development*.

### Dietary Indices and Circulating Concentrations of Insulin Response/IGF-Signaling Biomarkers

**Figure 2:**
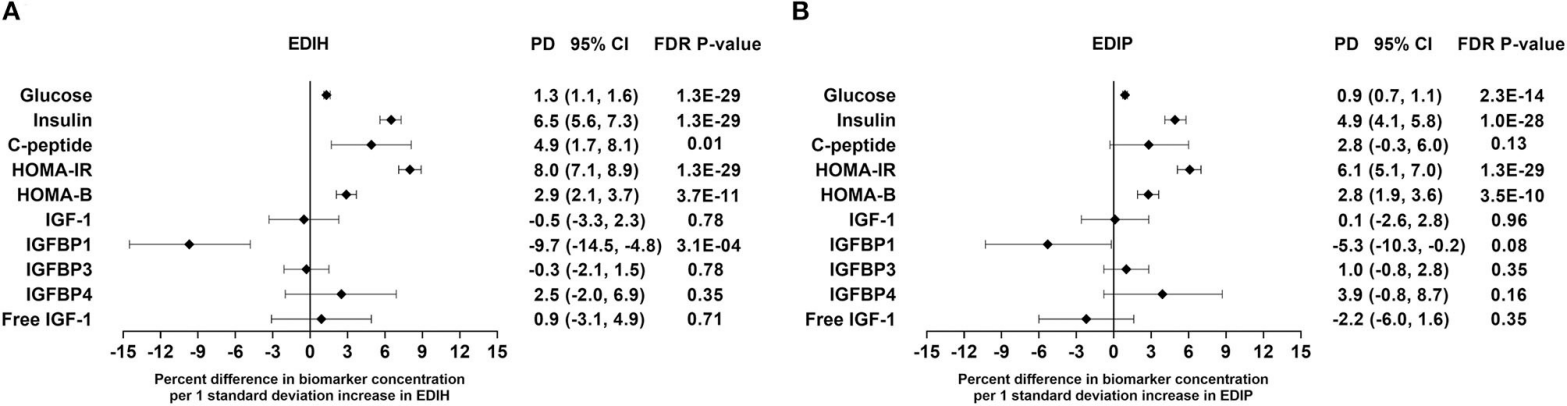
Percentage difference (PD—beta coefficients) (95% confidence intervals) in insulin response/IGF-signaling biomarker per 1 standard deviation increment in EDIH **(A)** and EDIP **(B)** in the Women's Health Initiative. Biomarkers were log-transformed, using natural logs. Values were obtained *via* multivariable-adjusted linear regression models adjusted for the following variables: total energy intake, BMI-continuous, age, total recreational physical activity, pack years of smoking; number of supplements used, fasting status at blood draw, race/ethnic groups, educational levels, regular use of NSAID, statins, unopposed estrogen and/or estrogen plus progesterone hormones, hormone therapy (HT) study arms. The sample sizes for each biomarker were as follows: glucose, 21,669; insulin, 23,756; C-peptide, 943; HOMA-IR, 19,865; HOMA-B, 19,865; insulin-like growth factor (IGF)-1, 3,126; IGF-binding protein (BP)-1, 993; IGF-BP3, 2,349; IGF-BP4, 354; free IGF-1, 2,203.

Intake of higher EDIH scores (representing more hyperinsulinemic dietary patterns) was associated with greater concentrations of all five insulin response markers, with the percent difference (PD) smallest for glucose (1.3%) and largest for insulin resistance (HOMA-IR, 8.%) (Figure 2A). EDIH was significantly associated with only one (IGFBP-1, −9.7%) of the five (IGF-1, free IGF-1, IGFBP-3, IGFBP-4) IGF-signaling biomarkers. Similarly, higher EDIP scores, reflecting more proinflammatory dietary patterns, were associated with greater concentration of the insulin response biomarkers, although the magnitude of associations was smaller: glucose (0.9%), HOMA-IR (6.1%), and IGFBP1 (−5.3%) (Figure 2B). The absolute concentrations of the insulin response/IGF-signaling biomarkers in each dietary index quintile and the percent difference between highest and lowest quintiles are presented in Table 2. The findings from these categorical analyses are consistent with the findings from the analyses with the continuous dietary scores.

**Table 2:** Multivariable-adjusted absolute concentration (95% CI) of circulating biomarkers of insulin response/IGF signaling in quintiles of the dietary indices.

**Statistical model^a^^,^^b^**	**Quintile 1**	**Quintile 2**	**Quintile 3**	**Quintile 4**	**Quintile 5**	**Percentage difference:** **Q5 – Q1**	**FDR *P*-Value**
	**Empirical Dietary Index for Hyperinsulinemia (EDIH) score quintiles**						
Glucose, mg/dL^c^ (*n* = 21,669)	95.3 (94.2, 96.4)	96.1 (95.0, 97.2)	96.6 (95.5, 97.7)	97.5 (96.3, 98.6)	99.0 (97.9, 100.2)	**3.8 (3.0, 4.7)**	**1.30E-29**
Insulin, uIU/mL^c^(*n* = 23,756)	8.4 (8.0, 8.8)	8.8 (8.4, 9.2)	9.0 (8.6, 9.4)	9.6 (9.2, 10.1)	10.1 (9.7, 10.6)	**18.7 (15.4, 22.0)**	**1.30E-29**
C-peptide, ng/mL^c^ (*n* = 943)	1.4 (1.1, 1.8)	1.4 (1.2, 1.8)	1.6 (1.2, 1.69)	1.5 (1.2, 1.9)	1.7 (1.3, 2.1)	**14.8 (2.8, 26.7)**	**0.008**
HOMA-IR^d^ (*n* = 19,865)	2.0 (1.9, 2.0)	2.1 (2.0, 2.2)	2.1 (2.0, 2.2)	2.3 (2.2, 2.4)	2.5 (2.3, 2.6)	**23.0 (19.4, 26.6)**	**1.30E-29**
HOMA-β^d^ (*n* = 19,865)	96.5 (92.3, 100.8)	98.2 (94.0, 102.6)	100.0 (95.8, 104.4)	103.8 (99.4, 108.4)	104.7 (100.2, 109.3)	**8.2 (5.0, 11.4)**	**3.71E-11**
IGF^d^-1, ng/mL^c^ (*n* = 3,126)	86.7 (57.6, 127.4)	79.7 (53.6, 118.5)	75.8 (50.9, 112.8)	78.5 (52.9, 116.6)	85.8 (57.8, 127.5)	0.2 (−10.4, 10.8)	0.777
IGFBP^d^1, ng/L^c^ (*n* = 993)	20.0 (14.4, 27.8)	19.3 (13.9, 26.7)	19.1 (13.7, 26.6)	16.8 (12.1, 23.3)	15.8 (11.4, 21.9)	**−23.4 (−41.6**, **−5.3)**	**0.0003**
IGFBP3, ng/mL (*n* = 2,349)	3,297 (2,867, 3,792)	3,481 (3,030, 3,999)	3,280 (2,853, 3,770)	3,389 (2,950, 3,893)	3,316 (2,891, 3,804)	0.6 (−6.2, 7.4)	0.777
IGFBP4, ng/mL^c^ (*n* = 354)	502 (401, 629)	444 (358, 552)	512 (408, 642)	538 (433, 669)	516 (415, 642)	2.7 (−15.0, 20.4)	0.355
free IGF-1, ng/mL^c^ (*n* = 2,203)	0.70 (0.55, 0.90)	0.73 (0.57, 0.93)	0.74 (0.58, 0.95)	0.72 (0.57, 0.92)	0.74 (0.58, 0.95)	6.0 (−9.1, 21.1)	0.712
	**Empirical Dietary Inflammatory Index (EDIP) score quintiles**						
Glucose, mg/dL (*n* = 21,669)	95.7 (94.6, 96.8)	96.3 (95.2, 97.4)	96.0 (94.9, 97.1)	97.4 (96.3, 98.6)	98.4 (97.3, 99.5)	**2.8 (1.9, 3.6)**	**2.26E-14**
Insulin, uIU/mL (*n* = 23,756)	8.4 (8.0, 8.8)	8.7 (8.3, 9.1)	9.0 (8.6, 9.4)	9.4 (9.0, 9.8)	9.9 (9.5, 10.3)	**16.0 (12.6, 19.4)**	**1.03E-28**
C-peptide, ng/mL (*n* = 943)	1.4 (1.1, 1.8)	1.5 (1.2, 1.9)	1.5 (1.2, 1.8)	1.6 (1.3, 2.0)	1.5 (1.2, 1.9)	4.7 (−7.4, 16.8)	0.135
HOMA-IR (*n* = 19,865)	2.0 (1.9, 2.1)	2.1 (2.0, 2.2)	2.1 (2.0, 2.2)	2.2 (2.1, 2.4)	2.4 (2.3, 2.5)	**18.6 (15.0, 22.3)**	**1.30E-29**
HOMA-β (*n* = 19,865)	96.0 (91.9, 100.4)	97.8 (93.6, 102.2)	101.6 (97.3, 106.2)	101.4 (97.1, 105.9)	104.0 (99.7, 108.6)	**8.0 (4.7, 11.3)**	**3.49E-10**
IGF-1, ng/mL (*n* = 3,126)	80.0 (53.6, 118.9)	83.4 (56.0, 124.2)	78.8 (53.0, 117.2)	81.9 (55.1, 121.8)	83.7 (56.4, 124.3)	4.7 (−5.9, 15.4)	0.955
IGFBP1, ng/L (*n* = 993)	19.6 (14.0, 27.3)	19.6 (14.1, 27.3)	17.2 (12.4, 24.1)	16.6 (11.9, 23.2)	17.7 (12.8, 24.5)	−10.0 (−28.5, 8.6)	0.077
IGFBP3, ng/mL (*n* = 2,349)	3,274 (2,847, 3,763)	3,328 (2,895, 3,826)	3,425 (2,981, 3,935)	3,344 (2,911, 3,842)	3,372 (2,938, 3,870)	3.0 (−3.8, 9.8)	0.353
IGFBP4, ng/mL (*n* = 354)	456 (636, 574)	492 (392, 618)	485 (391, 602)	528 (424, 658)	509 (409, 635)	11.0 (−6.8, 28.8)	0.163
free IGF-1, ng/mL (*n* = 2,203)	0.73 (0.57, 0.93)	075 (0.59, 0.95)	0.79 (0.62, 1.01)	0.74 (0.58, 0.95)	0.68 (0.53, 0.86)	−6.9 (−21.9, 8.0)	0.353

a *Values are absolute back-transformed biomarker concentrations (beta coefficients) since values were naturally log-transformed prior to analysis, and the bolded numbers represent statistically significant findings (i.e., FDR p < 0.05)*.

b *Values were adjusted for total energy intake, BMI-continuous, age, total recreational physical activity, pack years of smoking; number of supplements used; fasting status at blood draw, race/ethnic groups, educational levels, regular use of NSAID, statins, unopposed estrogen and/or estrogen plus progesterone hormones, hormone therapy (HT) study arms*.

c *Conversion to SI units: for blood glucose, to convert to mmol/L, multiply by.0555; for C-peptide, to convert to nmol/L, multiply by.331; for insulin, to convert to mIU/L, multiply by 1; for IGF-1, IGFBP-1, IGFBP-4, and free IGF-1, to convert to nmol/L, multiply by.131*.

d *HOMA-IR, homeostatic model assessment of insulin resistance; HOMA β, homeostatic model assessment of β-cell function; IGF-1, insulin-like growth factor-1; IGF-BP1/3/4, insulin-like growth factor-binding protein 1/3/4*.

### Dietary Indices and Odds of Prediabetes and Type 2 Diabetes

In this subsample analysis, characteristics of the 570 participants with HbA1c and fasting plasma glucose data were similar to the overall sample (Supplementary Table 4). For each one SD increase in the EDIH score, there were 33% higher odds of prediabetes and type 2 diabetes combined (OR, 1.33; 95% CI, 1.07, 1.66) and 47% higher odds of type 2 diabetes (OR, 1.47; 95% CI, 1.10, 1.96). Similarly, for each one SD increment in the EDIP score, there were 50% higher odds of prediabetes and type 2 diabetes combined (OR, 1.50; 95% CI, 1.15, 1.97) and 87% higher odds of type 2 diabetes (OR, 1.87; 95% CI, 1.25, 2.80). For both dietary patterns, odds of prediabetes were elevated but did not attain statistical significance (Table 3).

**Table 3:** Odds ratios (95% CI) for the associations of diet indices with diabetes.

		**Quartile 1**	**Quartile 2**	**Quartile 3**	**Quartile 4**	**Per 1 SD increment^a^**	**P trend**
		**Empirical Dietary Index for Hyperinsulinemia (EDIH) score quartiles**					
Prediabetes and diabetes^b^	Case/Non-case	15/127	22/121	39/104	37/105		
	Odds ratios	1 (ref)	1.54 (0.73, 3.27)	2.92 (1.45, 5.87)	1.97 (0.96, 4.02)	**1.33 (1.07, 1.66)**	**0.0109**
Diabetes	Case/Non-case	7/127	7/121	21/104	20/105		
	Odds ratios	1 (ref)	0.96 (0.31, 2.97)	3.21 (1.24, 8.33)	2.09 (0.79, 5.58)	**1.47 (1.10, 1.96)**	**0.0098**
Prediabetes	Case/Non-case	8/127	15/121	18/104	17/105		
	Odds ratios	1 (ref)	1.92 (0.75, 4.91)	2.41 (0.95, 6.07)	1.73 (0.67, 4.45)	1.16 (0.87, 1.55)	0.3007
		**Empirical Dietary Inflammatory Index (EDIP) score quartiles**					
Prediabetes and diabetes	Case/Non-case	9/133	32/111	28/115	44/98		
	Odds ratios	1 (ref)	4.35 (1.92, 9.85)	3.07 (1.33, 7.08)	4.72 (2.08, 10.7)	**1.50 (1.15, 1.97)**	**0.0029**
Diabetes	Case/Non-case	3/133	10/111	17/115	25/98		
	Odds ratios	1 (ref)	3.72 (0.97, 14.3)	5.31 (1.45, 19.4)	7.09 (1.97, 25.6)	**1.87 (1.25, 2.80)**	**0.0024**
Prediabetes	Case/Non-case	6/133	22/111	11/115	19/98		
	Odds ratios	1 (ref)	4.41 (1.67, 11.7)	1.86 (0.64, 5.40)	3.09 (1.10, 8.64)	1.22 (0.88, 1.71)	0.2384

a *Values are odds ratios, adjusted for total energy intake, BMI-continuous, age, total recreational physical activity, pack years of smoking; number of supplements used; fasting status at blood draw, race/ethnic groups, educational levels, regular use of NSAID, statins, unopposed estrogen and/or estrogen plus progesterone hormones, hormone therapy (HT) study arms. Quartile 1 was used as the reference. The bolded numbers represent statistically significant (p < 0.05)*.

b *Prediabetes and type 2 diabetes defined based on the American Diabetes Association (ADA) criteria: prediabetes—HbA1c 5.7–6.4% or impaired fasting glucose (IFG) 100–125 mg/dL; and type 2 diabetes—HbA1c ≥ 6.5% and IFG ≥ 126 mg/dL*.

### Dietary Indices and Circulating Concentrations of Inflammatory and Endothelial Dysfunction Biomarkers

**Figure 3:**
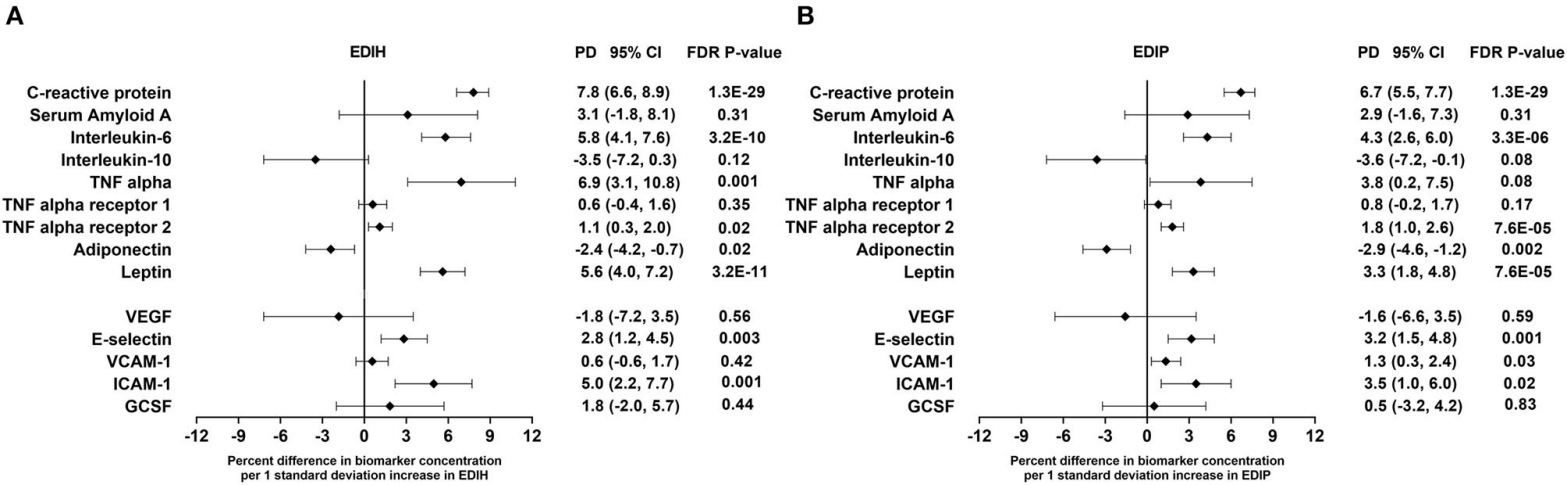
Percentage difference (PD—beta coefficients) (95% confidence intervals) in inflammation and endothelial dysfunction biomarkers biomarker per 1 standard deviation increment in EDIH **(A)** and EDIP **(B)** in the Women's Health Initiative. Biomarkers are log-transformed using natural logs. Values were obtained *via* multivariable-adjusted linear regression models adjusted for the following variables: total energy intake, BMI-continuous, age, total recreational physical activity, pack years of smoking; number of supplements used; fasting status at blood draw, race/ethnic groups, educational levels, regular use of NSAID, statins, unopposed estrogen and/or estrogen plus progesterone hormones, hormone therapy (HT) study arms. The sample size for each biomarker was as follows: C-reactive protein, 26,482; Serum Amyloid A, 1,181; Interleukin-6, 12,408; Interleukin-10, 2,466; TNF alpha, 5,302; TNF alpha receptor 13,908; TNF alpha receptor 27,746; adiponectin, 7,552; leptin, 8,045; vascular endothelial growth factor (VEGF), 873; E-selectin, 3,817; vascular cell adhesion molecule (VCAM), 14,050; intercellular adhesion molecule (ICAM), 11,075; granulocyte colony-stimulating factor (GCSF), 1,413.

Higher EDIH scores were associated with greater concentration of five out of the nine inflammatory biomarkers and with lower concentrations of adiponectin. For a one SD increment in the EDIH score, the percent differences in biomarker concentrations were as follows: CRP, 7.8%; IL-6, 5.8%; TNF-α, 6.9%; TNF-αR2, 1.1%; leptin, 5.6%; and adiponectin, −3.1%. EDIH was not associated with SAA and TNF-αR1 (Figure 3A). EDIP followed a similar trend with higher EDIP scores associated with greater concentrations of four out of the nine inflammatory biomarkers and with lower concentrations of adiponectin; and with percent differences as follows: CRP, 6.7%; IL-6, 4.3%; TNF-αR2, 1.8%; leptin, 3.3%; and adiponectin, −2.9% (Figure 3B). EDIH and EDIP both had an inverse trend of associations with IL-10, although not statistically significant. For the five endothelial dysfunction biomarkers, higher EDIH scores were associated with greater concentration of E-selectin (2.8%) and ICAM-1 (5.%) (Figure 3A). Higher EDIP scores were associated with greater concentration of E-selectin (3.2%), ICAM-1 (1.3%), and VCAM-1 (3.5%) (Figure 3B). Absolute concentrations of these inflammatory biomarkers in dietary score quintiles and the percent difference between highest and lowest quintiles are presented in Table 4.

**Table 4:** Multivariable-adjusted absolute concentrations (95% CI) of circulating biomarkers of inflammation and endothelial dysfunction in quintiles of the dietary indices.

**Statistical model^a^^,^^b^**	**Quintile 1**	**Quintile 2**	**Quintile 3**	**Quintile 4**	**Quintile 5**	**Percentage difference:** **Q5 – Q1**	**FDR *P*-Value**
	**Empirical Dietary Index for Hyperinsulinemia (EDIH) score quintiles**						
C-reactive protein, mg/L^c^ (*n* = 26,482)	1.6 (1.5, 1.7)	1.8 (1.6, 1.9)	1.8 (1.7, 1.9)	1.9 (1.8, 2.0)	2.0 (1.9, 2.2)	**23.1 (18.8, 27.5)**	**1.30E-29**
Serum Amyloid A, mg/L (*n* = 1,181)	13.2 (8.1, 21.5)	13.2 (8.2, 21.3)	12.8 (7.8, 20.8)	13.7 (8.4, 22.2)	14.2 (8.8, 22.9)	7.5 (−9.7, 24.7)	0.306
Interleukin-6, pg/mL (*n* = 12,408)	2.6 (2.3, 2.8)	2.7 (2.4, 3.0)	2.7 (2.5, 3.0)	2.9 (2.6, 3.3)	3.0 (2.7, 3.3)	**16.3 (9.7, 23.0)**	**3.15E-10**
Interleukin 10, pg/L (*n* = 2,466)	3.7 (2.8, 4.8)	3.7 (2.9, 4.9)	3.6 (2.7, 4.6)	3.3 (2.5, 4.3)	3.3 (2.6, 4.3)	−11.1 (−25.4, 3.2)	0.118
TNF^d^ alpha, pg/mL (*n* = 5,302)	10.4 (7.9, 13.7)	10.6 (8.0, 14.0)	10.7 (8.1, 14.2)	12.2 (9.2, 16.1)	12.9 (9.8, 17.0)	**21.9 (7.5, 36.3)**	**0.001**
TNF alpha receptor 1, pg/mL (*n* = 3,908)	1,335 (1,259, 1,415)	1,359 (1,282, 1,440)	1,386 (1,308, 1,469)	1,346 (1,270, 1,426)	1,365 (1,289, 1,447)	2.3 (−1.4, 6.0)	0.353
TNF alpha receptor 2, ng/mL (*n* = 7,746)	4.7 (3.8, 5.9)	4.7 (3.8, 5.9)	4.8 (3.9, 6.0)	4.8 (3.8, 5.9)	4.8 (3.9, 6.1)	2.9 (−0.3, 6.1)	0.024
Adiponectin, ng/mL (*n* = 7,552)	9,103 (8,155, 10,161)	8,961 (8,028, 10,003)	8,577 (7,685, 9,572)	8,555 (7,672, 9,541)	8,610 (7,719, 9,605)	−5.6 (−12.0, 0.9)	0.0167
Leptin, ng/mL^c^ (*n* = 8,045)	22.5 (16.6, 30.5)	23.7 (17.5, 32.1)	25.6 (18.9, 34.6)	26.1 (19.3, 35.4)	26.2 (19.3, 35.5)	**15.1 (9.2, 21.0)**	**3.16E-11**
VEGF^d^, pg.ml (*n* = 873)	149 (87.3, 255)	154 (91.1, 262)	148 (86.8, 252)	155 (91.5, 264)	153 (89.6, 262)	2.7 (−16.5, 21.8)	0.558
E-selectin, ng/ml (*n* = 3,817)	43.7 (39.2, 48.8)	43.5 (39.0, 48.5)	41.8 (37.5, 46.6)	46.1 (41.3, 51.3)	46.1 (41.3, 51.4)	5.2 (−1.1, 11.6)	0.003
VCAM-1^d^, ng.ml (*n* = 4,050)	741 (670, 821)	733 (662, 811)	730 (660, 808)	742 (671, 820)	756 (684, 836)	1.9 (−2.2, 6.1)	0.415
ICAM-1^d^, ng/ml (*n* = 1,075)	181 (142, 231)	183 (144, 232)	186 (146, 237)	199 (157, 252)	212 (167, 269)	**15.5 (5.7, 25.4)**	**0.001**
GCSF^d^, pg/ml (*n* = 1,413)	140 (115, 169)	150 (124, 182)	141 (117, 171)	149 (123, 180)	146 (121, 177)	4.6 (−10.3, 19.6)	0.440
	**Empirical Dietary Inflammatory Index (EDIP) score quintiles**						
C-reactive protein, mg/L (*n* = 26,482)	1.6 (1.5, 1.7)	1.8 (1.7, 1.9)	1.8 (1.7, 1.9)	1.9 (1.8, 2.0)	2.0 (1.9, 2.1)	**18.6 (14.1, 23.0)**	**1.30E-29**
Serum Amyloid A, mg/L (*n* = 1,181)	12.8 (7.9, 20.8)	12.7 (7.8, 20.6)	13.8 (8.5, 22.4)	13.8 (8.5, 22.3)	14.4 (8.8, 22.7)	9.4 (−7.9, 26.8)	0.306
Interleukin-6, pg/mL (*n* = 12,408)	2.6 (2.3, 2.9)	2.8 (2.5, 3.1)	2.8 (2.5, 3.1)	2.8 (2.5, 3.1)	3.0 (2.7, 3.3)	**16.0 (9.3, 22.7)**	**3.30E-06**
Interleukin 10, pg/L (*n* = 2,466)	3.9 (3.0, 5.0)	3.5 (2.7, 4.6)	3.4 (2.6, 4.4)	3.4 (2.6, 4.4)	3.4 (2.6, 4.4)	−13.5 (−28.0, 1.1)	0.079
TNF alpha, pg/mL (*n* = 5,302)	10.7 (8.1, 14.2)	10.6 (8.1, 14.1)	10.6 (8.0, 14.0)	12.3 (9.3, 16.1)	12.6 (9.6, 16.6)	16.1 (1.6, 30.5)	0.077
TNF alpha receptor 1, pg/mL (*n* = 3,908)	1,319 (1,244, 1,399)	1,357 (1,281, 1,439)	1,375 (1,298, 1,456)	1,359 (1,283, 1,440)	1,371 (1,294, 1,453)	3.9 (0.2, 7.6)	0.169
TNF alpha receptor 2, ng/mL (*n* = 7,746)	4.7 (3.8, 5.9)	4.8 (3.8, 6.0)	4.8 (3.9, 6.0)	4.9 (3.9, 6.1)	4.9 (3.9, 6.1)	**4.6 (1.4, 7.9)**	**7.56E-05**
Adiponectin, ng/mL (*n* = 7,552)	9,234 (8,271, 10,309)	8,959 (8,021, 10,006)	8,576 (7,685, 9,571)	8,675 (7,779, 9,674)	8,482 (7,606, 9,458)	**−8.5 (−15.1**, **−1.9)**	**0.0023**
Leptin, ng/mL (*n* = 8,045)	23.3 (17.2, 31.6)	24.5 (18.1, 33.2)	25.7 (18.9, 34.8)	25.9 (19.1, 35.1)	25.2 (18.6, 34.2)	**77.8 (18.1, 137)**	**7.56E-05**
VEGF, pg.ml (*n* = 873)	156 (92.3, 265)	152 (89.3, 158)	164 (96.3, 280)	151 (88.5, 257)	144 (84.4, 246)	−8.3 (−28.0, 11.5)	0.588
E-selectin, ng/ml (*n* = 3,817)	42.2 (37.8, 47.1)	43.8 (39.3, 48.9)	42.9 (38.5, 47.9)	44.8 (40.2, 50.0)	46.7 (41.9, 52.1)	**10.3 (3.8, 16.7)**	**0.00056**
VCAM-1, ng.ml (*n* = 4,050)	729 (659, 807)	726 (656, 803)	734 (664, 812)	746 (674, 825)	765 (692, 846)	**4.8 (0.6, 9.0)**	**0.0334**
ICAM-1, ng/ml (*n* = 1,075)	186 (146, 237)	188 (148, 239)	191 (151, 242)	204 (161, 260)	212 (167, 269)	**13.0 (2.8, 23.1)**	**0.018**
GCSF, pg/ml (*n* = 1,413)	148(122, 179)	146 (121, 177)	137 (113, 165)	156 (129, 188)	146 (121, 176)	−1.4 (−16.6, 13.9)	0.827

a *Values are absolute back-transformed biomarker concentrations (beta coefficients) since values were naturally log-transformed prior to analysis, and the bolded numbers represent statistically significant findings (i.e., FDR p < 0.05)*.

b *Values were adjusted for total energy intake, BMI-continuous, age, total recreational physical activity, pack years of smoking; number of supplements used; fasting status at blood draw, race/ethnic groups, educational levels, regular use of NSAID, statins, unopposed estrogen and/or estrogen plus progesterone hormones, hormone therapy (HT) study arms*.

c *Conversion to SI units: for C-reactive protein, mg/L is SI unit; for leptin, μg/L is SI unit; for other biomarkers, SI units are not readily available*.

d *TNF, tumor necrosis factor; VEGF, vascular endothelial growth factor; VCAM-1, vascular cell adhesion protein 1; ICAM-1, intercellular adhesion molecule 1; GCSF, granulocyte colony-stimulating factor*.

### Dietary Indices and Circulating Concentrations of Lipid Biomarkers

**Figure 4:**
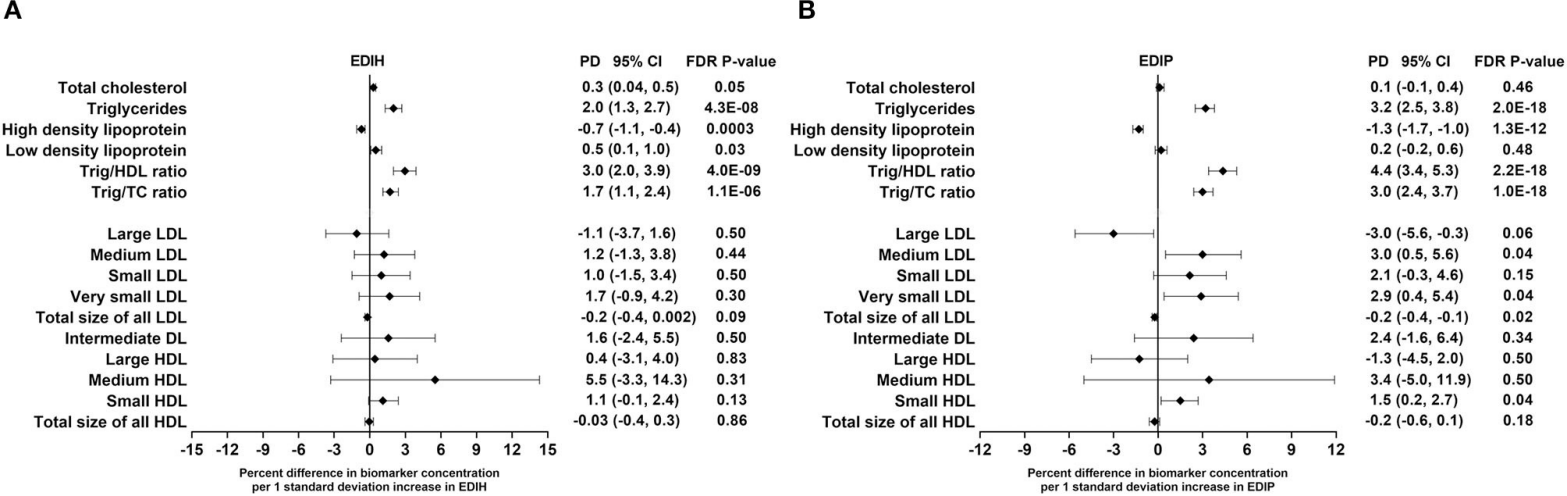
Percentage difference (PD—beta coefficients) (95% confidence intervals) in lipid and lipid particles biomarkers biomarker per 1 standard deviation increment in EDIH **(A)** and EDIP **(B)** in the Women's Health Initiative. Biomarkers were log transformed using natural logs. Values were obtained *via* multivariable-adjusted linear regression models adjusted for the following variables: total energy intake, BMI-continuous, age, total recreational physical activity, pack years of smoking; number of supplements used; fasting status at blood draw, race/ethnic groups, educational levels, regular use of NSAID, statins, unopposed estrogen and/or estrogen plus progesterone hormones, hormone therapy (HT) study arms. The sample sizes for each biomarker were as follows: total cholesterol (TG), 21,378; triglycerides (TC), 18,833; high-density lipoprotein (HDL), 20,508; low-density lipoprotein (LDL), 16,525; TG/HDL ratio, 17,761; TG/TC ratio, 18,631; large LDL, 1,653; medium LDL, 1,356; small LDL, 1,653; very small LDL, 1,356; total size of all LDL, 1,652; intermediate density lipoprotein, 1,653; large HDL, 1,653; medium HDL, 1,241; small HDL, 1,653; total size of all HDL, 1,653.

EDIH was significantly associated with the lipid panel as follows (for one SD increments): TC, 0.3%; TG, 2.%; HDL, −0.7%; LDL, 0.5%; TG/HDL ratio, 3.%; TG/TC ratio, 1.7%. The magnitude of associations was slightly higher for EDIP: TG, 3.2%; HDL, −1.3%; TG/HDL ratio, 4.4%; TG/TC ratio, 3.%; although there was no significant association with TC and LDL (Figure 4A). EDIH was generally not associated with lipid particles of differing sizes; however, higher EDIP was associated with higher medium LDL (3%), very small LDL (2.9%), and with lower large LDL (−3%) and total size of all LDL particles (−0.2%) (Figure 4B). Absolute concentrations of lipids and lipid particles in dietary index quintiles and the percent difference between highest and lowest quintiles are presented in Table 5.

**Table 5:** Multivariable-adjusted percent difference (95% CI) in the relative concentrations of circulating lipid and lipid particle biomarkers in quintiles of the dietary indices.

**Statistical model^a^^,^^b^**	**Quintile 1**	**Quintile 2**	**Quintile 3**	**Quintile 4**	**Quintile 5**	**Percentage difference:** **Q5 – Q1**	**FDR *P*-Value**
	**Empirical Dietary Index for Hyperinsulinemia (EDIH) score quintiles**						
Total cholesterol, mg/dL^c^ (*n* = 21,378)	227 (224, 230)	227 (224, 231)	227 (224, 230)	228 (225, 231)	229 (226, 322)	0.78 (−0.2, 1.8)	0.0501
Triglycerides, mg/dL^c^ (*n* = 18,833)	138 (133, 143)	141 (136, 146)	141 (136, 146)	144 (139, 150)	147 (142, 153)	**6.5 (3.8, 9.2)**	**4.32E-08**
High density Lipoprotein (HDL), mg/dL^c^ (*n* = 20,508)	52.6 (51.6, 53.6)	52.6 (51.6, 53.6)	52.5 (51.5, 53.5)	51.9 (51.0, 52.6)	51.3 (50.4, 52.3)	–**2.5 (**–**3.9**, –**1.1)**	**0.0003**
Low density Lipoprotein (LDL), mg/dL^c^ (*n* = 16,525)	141 (137, 144)	141 (138, 144)	141 (137, 144)	141 (138, 145)	143 (140, 146)	**1.7 (0.01, 3.3)**	**0.034**
Triglycerides/High density Lipoprotein (*n* = 17,761)	2.6 (2.5, 2.8)	2.7 (2.6, 2.8)	2.7 (2.6, 2.8)	2.8 (2.6, 2.9)	2.9 (2.7, 3.0)	**9.8 (6.1, 13.5)**	**4.00E-09**
Triglycerides/Total cholesterol (*n* = 18,631)	0.61 (0.59, 0.63)	0.62 (0.59, 0.64)	0.62 (0.60, 0.64)	0.63 (0.61, 0.65)	0.64 (0.62, 0.66)	**5.7 (3.2, 8.2)**	**1.10E-06**
Large LDL, nmol/L (*n* = 1,653)	547 (466, 628)	537 (456, 617)	547 (467, 626)	536 (456, 617)	528 (449, 608)	−3.4 (−12.6, 5.9)	0.500
Medium LDL, nmol/L (*n* = 1,356)	223 (190, 256)	225 (192, 258)	222 (189, 255)	224 (192, 257)	230 (198, 263)	3.2 (−5.8, 12.2)	0.440
Small LDL, nmol/L (*n* = 1,653)	1,078 (927, 1,230)	1,052 (903, 1,202)	1,062 (913, 1,210)	1,054 (904, 1,204)	1,109 (960, 1,258)	2.9 (−6.0, 11.7)	0.500
Very small LDL, nmol/L (*n* = 1,356)	928 (791, 1,066)	934 (797, 1,071)	929 (794, 1,065)	931 (796, 1,067)	970 (935, 1,105)	4.5 (−4.5, 13.4)	0.297
Total size of all LDL, nm (*n* = 1,652)	20.8 (20.6, 21.1)	20.8 (20.6, 21.0)	20.8 (20.6, 21.1)	20.8 (20.6, 21.0)	20.7 (20.5, 21.0)	−0.4 (−1.1, 0.2)	0.0923
Intermediate density lipoprotein, nmol/L (*n* = 1,653)	126 (98, 155)	128 (99, 156)	121 (93, 149)	125 (97, 154)	137 (109, 164)	7.9 (−6.1, 22.0)	0.500
Large HDL, nmol/L (*n* = 1,653)	5.0 (4.0, 6.0)	5.2 (4.2, 6.2)	5.3 (4.3, 6.3)	5.0 (4.0, 6.0)	5.0 (4.0, 6.0)	0.7 (−11.9, 13.4)	0.829
Medium HDL, nmol/L (*n* = 1,241)	1.7 (1.0, 3.0)	2.0 (1.1, 3.4)	1.5 (0.9, 2.6)	2.0 (1.2, 3.5)	2.1 (1.2, 3.6)	16.5 (−15.3, 48.4)	0.312
Small HDL, nmol/L (*n* = 1,653)	24.2 (22.5, 26.0)	24.0 (22.3, 25.8)	24.6 (22.9, 26.4)	24.4 (22.6, 26.1)	24.8 (23.1, 26.6)	2.5 (−2.1, 7.0)	0.132
Total size of all HDL, nm (*n* = 1,653)	8.8 (8.6, 8.9)	8.8 (8.6, 9.0)	8.8 (8.6, 9.0)	8.7 (8.6, 8.9)	8.8 (8.6, 8.9)	−0.1 (−1.3, 1.0)	0.865
	**Empirical Dietary Inflammatory Index (EDIP) score quintiles**						
Total cholesterol, mg/dL (*n* = 21,378)	227 (224, 230)	229 (225, 232)	227 (224, 230)	227 (224, 230)	228 (225, 231)	0.5 (−0.5, 1.5)	0.459
Triglycerides, mg/dL (*n* = 18,833)	134 (129, 139)	140 (135, 145)	140 (135, 146)	146 (141, 151)	148 (143, 153)	**9.9 (7.2, 12.6)**	**2.04E-18**
High density Lipoprotein (HDL), mg/dL (*n* = 20,508)	53.4 (52.4, 54.5)	52.8 (51.8, 53.8)	52.3 (51.3, 53.3)	51.9 (50.9, 52.9)	51.2 (50.2, 52.2)	−4.3 (−5.7, 2.9)	1.27E-12
Low density Lipoprotein (LDL), mg/dL (*n* = 16,525)	140 (137, 143)	142 (139, 145)	141 (138, 144)	141 (137, 144)	142 (139, 146)	1.3 (−0.4, 3.0)	0.480
Triglycerides/High density Lipoprotein (*n* = 17,761)	2.5 (2.4, 2.7)	2.7 (2.5, 2.8)	2.7 (2.6, 2.8)	2.8 (2.7, 3.0)	2.9 (2.8, 3.0)	**13.8 (10.0, 17.6)**	**2.16E-18**
Triglycerides/Total cholesterol (*n* = 18,631)	0.59 (0.57, 0.61)	0.61 (0.59, 0.63)	0.62 (0.60, 0.64)	0.64 (0.62, 0.66)	0.65 (0.62, 0.67)	**9.2 (6.6, 11.7)**	**1.00E-18**
Large LDL, nmol/L (*n* = 1,653)	559 (478, 640)	554 (473, 635)	551 (471, 630)	525 (445, 605)	515 (435, 595)	−8.6 (−18.6, 1.4)	0.056
Medium LDL, nmol/L (*n* = 1,356)	214 (181, 247)	224 (191, 257)	227 (194, 260)	225 (193, 258)	232 (199, 265)	8.3 (−1.2, 17.7)	0.042
Small LDL, nmol/L (*n* = 1,653)	1,031 (881, 1,182)	1,089 (938, 1,239)	1,060 (911, 1,209)	1,081 (932, 1,230)	1,103 (953, 1,252)	6.9 (−2.4, 16.2)	0.148
Very small LDL, nmol/L (*n* = 1,356)	898 (761, 1,035)	937 (799, 1,074)	945 (810, 1,081)	941 (807, 1,076)	970 (835, 1,107)	8.1 (−1.2, 17.5)	0.047
Total size of all LDL, nm (*n* = 1,652)	20.9 (20.7, 21.1)	20.8 (20.6, 21.0)	20.8 (20.6, 21.0)	20.8 (20.6, 21.0)	20.7 (20.5, 20.9)	–**0.8 (**–**1.4**, –**0.1)**	**0.018**
Intermediate density lipoprotein, nmol/L (*n* = 1,653)	120 (91.3, 148)	128 (100, 156)	125 (97.3, 153)	132 (104, 160)	132 (104, 160)	10.4 (−4.7, 25.5)	0.343
Large HDL, nmol/L (*n* = 1,653)	5.2 (4.2, 6.2)	5.3 (4.3, 6.3)	5.2 (4.2, 6.1)	5.0 (4.0, 6.0)	5.0 (4.0, 6.0)	−4.2 (−16.4, 8.0)	0.500
Medium HDL, nmol/L (*n* = 1,241)	1.8 (1.0, 3.1)	1.7 (1.0, 3.0)	1.8 (1.0, 3.1)	2.0 (1.1, 3.4)	2.1 (1.2, 3.6)	15.5 (−16.5, 47.6)	0.500
Small HDL, nmol/L (*n* = 1,653)	23.9 (22.1, 25.6)	24.7 (22.9, 26.4)	24.3 (22.6, 26.0)	25.0 (23.3, 26.8)	24.5 (22.7, 26.2)	2.4 (−2.2, 7.1)	**0.042**
Total size of all HDL, nm (*n* = 1,653)	8.8 (8.7, 9.0)	8.8 (8.6, 8.9)	8.8 (8.6, 8.9)	8.8 (8.6, 8.9)	8.7 (8.6, 8.9)	−0.8 (−2.0, 0.3)	0.179

a *Values are absolute back-transformed biomarker concentrations (beta coefficients) since values were naturally log-transformed prior to analysis, and the bolded numbers represent statistically significant findings (i.e., FRD p < 0.05)*.

b *Values were adjusted for total energy intake, BMI-continuous, age, total recreational physical activity, pack years of smoking; number of supplements used; fasting status at blood draw, race/ethnic groups, educational levels, regular use of NSAID, statins, unopposed estrogen and/or estrogen plus progesterone hormones, hormone therapy (HT) study arms*.

c *Conversion to SI units: for triglycerides, to convert from mg/dL to mmol/L, multiply by.0113; for LDL, HDL, and total cholesterol, to convert from mg/dL to mmol/L, multiply by.0259*.

### Associations of Dietary Indices and Biomarkers in Subgroups of Potential Effect Modifiers

**Figure 5:**
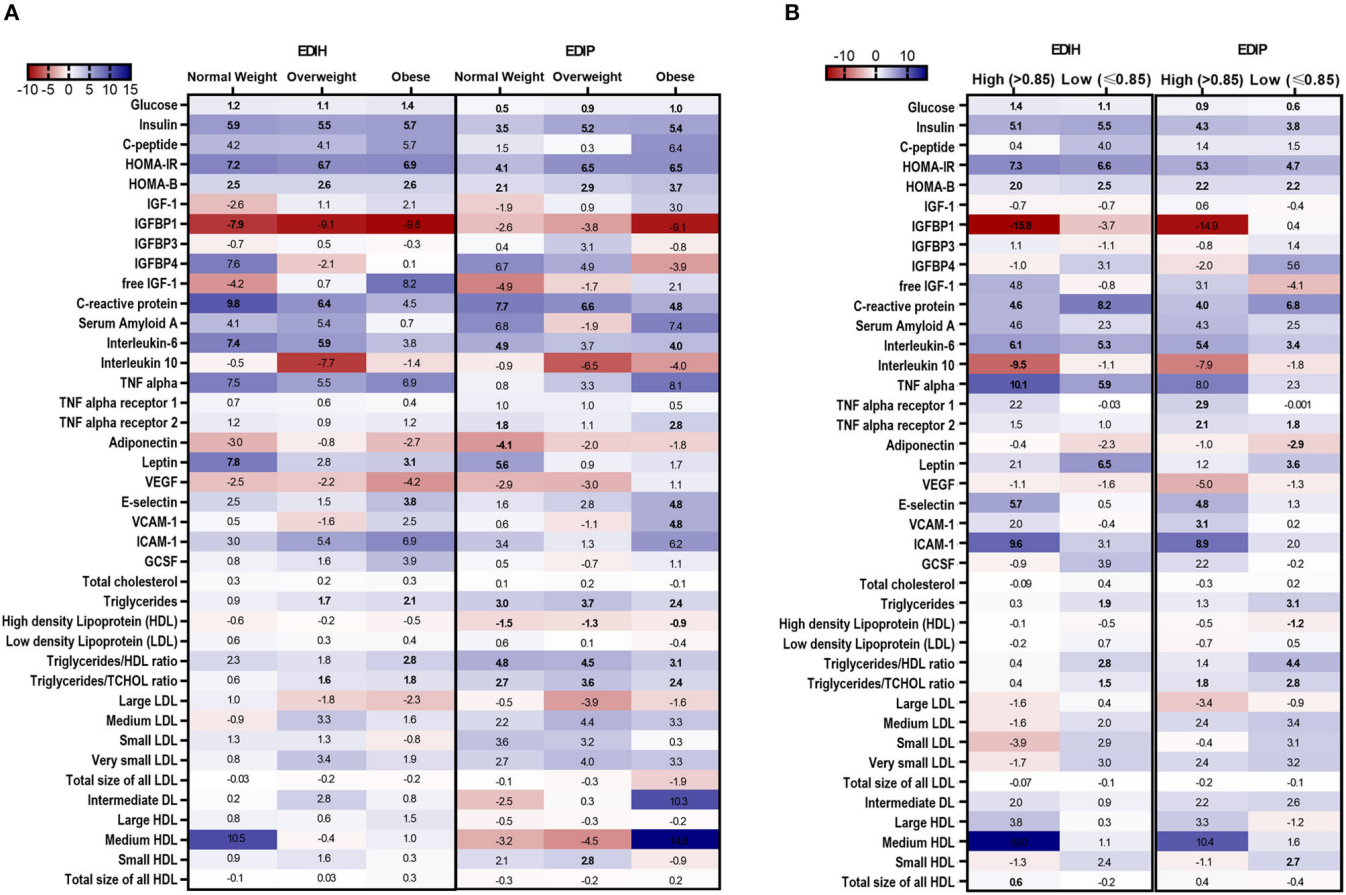
Heat map showing the percentage difference (PD) in biomarker concentrations for each 1 standard deviation increment in the dietary index score in subgroups of BMI and waist-to-hip ratio (WHR). **(A)** BMI subgroups: normal weight, 18.5–24.9; overweight, 25–29.9; obesity BMI, 30–50 kg/m^2^. **(B)** WHR subgroups: high WHR > 0.85; low WHR ≤ 0.85. Biomarker concentrations were log transformed using natural logs. Values (beta coefficients) presented were obtained *via* multivariable-adjusted linear regression models adjusted for the following variables: total energy intake, BMI-continuous, age, total recreational physical activity, pack years of smoking; number of supplements used; fasting status at blood draw, race/ethnic groups, educational levels, regular use of NSAID, statins, unopposed estrogen and/or estrogen plus progesterone hormones, hormone therapy (HT) study arms. The color gradient ranges from red (inverse associations) to blue (positive associations). Values in bold font were statistically significant (FDR *p* < 0.10).

**Figure 6:**
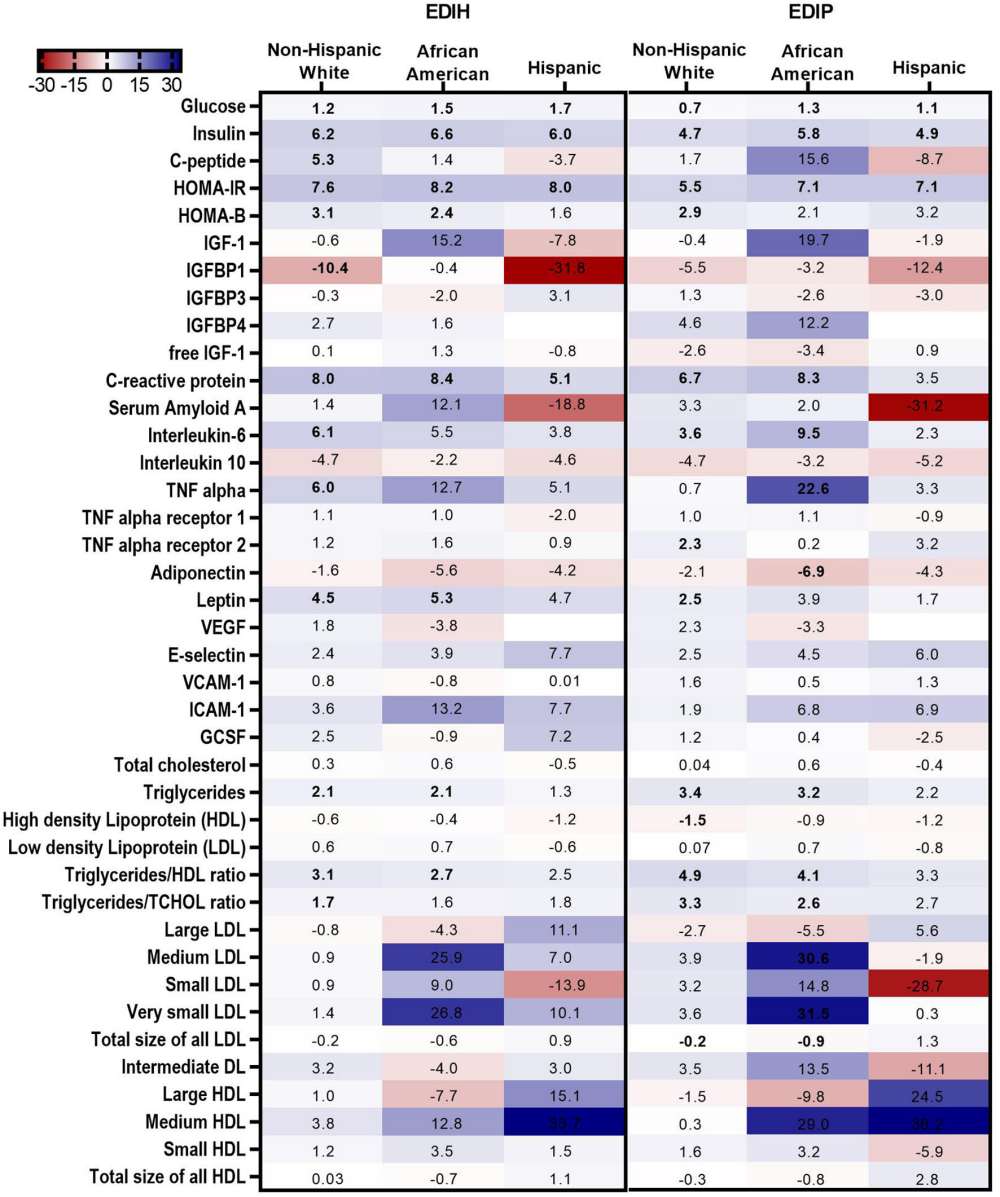
Heat map showing the percentage difference (PD) in biomarker concentrations for each 1 standard deviation increment in the dietary index score in race/ethnic categories. Biomarker concentrations were log transformed using natural logs. Values (beta coefficients) presented were obtained *via* multivariable-adjusted linear regression models adjusted for the following variables: total energy intake, BMI-continuous, age, total recreational physical activity, pack years of smoking; number of supplements used; fasting status at blood draw, educational levels, regular use of NSAID, statins, unopposed estrogen and/or estrogen plus progesterone hormones, hormone therapy (HT) study arms. The color gradient ranges from red (inverse associations) to blue (positive associations). Values in bold font were statistically significant (FDR *p* < 0.10). Small sample sizes in race groups, such as Asian/Pacific Islander and American Indian/Alaskan Native, could not allow for subgroup analyses.

In BMI subgroups (Figure 5A; Supplementary Table 5), there were significant interactions between BMI and EDIH for glucose, insulin, HOMA-IR, and HOMA-β, but no clinically meaningful differences in these markers between BMI categories. Generally, there were no clear patterns of associations between BMI categories. Among categories of WHR, both dietary indices showed stronger associations with IGFBP-1, IL-10 (both inverse), TNF-α, TNF-αR1, E-selectin, ICAM-1 among women with high WHR; and stronger associations with TG, HDL, TG/HDL ratio and TG/TC ratio among those with low WHR, although interactions were not always significant (Figure 5B; Supplementary Table 6). In subgroups defined by race/ethnicity, interactions were, generally, not significant, although the dietary patterns had particularly stronger associations among African-American women for some biomarkers: TNF-α, adiponectin, medium LDL, and very small LDL (Figure 6; Supplementary Table 7).

**Figure 7:**
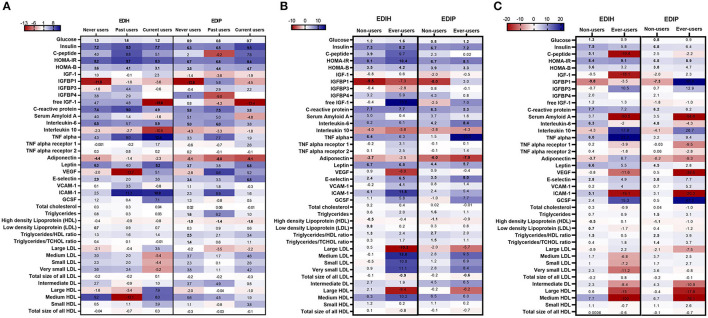
Heat map showing the percentage difference (PD) in biomarker concentrations for each 1 standard deviation increment in the dietary index score in subgroups defined by **(A)** estrogen usage status, **(B)** NSAIDs use and **(C)** statin use. Biomarker concentrations were log transformed using natural logs. Values (beta coefficients) presented were obtained *via* multivariable-adjusted linear regression models adjusted for the following variables: total energy intake, BMI-continuous, age, total recreational physical activity, pack years of smoking; number of supplements used; fasting status at blood draw, race/ethnic groups, educational levels, regular use of NSAID, statins, unopposed estrogen and/or estrogen plus progesterone hormones, hormone therapy (HT) study arms, except when the covariate was the potential effect modifier. The color gradient ranges from red (inverse associations) to blue (positive associations). Values in bold font were statistically significant (FDR *p* < 0.10).

Associations between the dietary indices and biomarkers were mainly limited to those who reported not regularly using statins: glucose, insulin, C-peptide, HOMA-IR, HOMA-β, and IGFBP1 for the insulin-related biomarkers; CRP, IL-6, adiponectin, leptin, E-selectin, and ICAM-1 for the inflammation/endothelial dysfunction biomarkers; HDL and TG/HDL ratio for lipids (Figure 7A; Supplementary Table 8). For NSAIDs subgroup analyses, the within-group significant associations for the insulin-related and inflammatory/endothelial dysfunction biomarkers were found among non-users and regular users alike (Figure 7; Supplementary Table 9). Interactions between the dietary indices and estrogen usage status were also not significant. Higher scores of both dietary indices were associated with higher levels of insulin, glucose, HOMA-IR, HOMA-β, CRP, and IL-6 in all three estrogen use categories and strongly associated with lower levels of IGFBP-1 levels mainly among never users (Figure 7C; Supplementary Table 10).

## Discussion

The evolution of research on diet and chronic diseases has for decades employed a reductionist approach focusing upon specific nutrients and foods with findings informing public health recommendations for healthy eating and disease prevention. Improvements in dietary assessment tools, establishment of large cohort studies, and banking of blood samples for biomarker evaluation, coupled with advances in biostatical approaches, has created an opportunity for the evaluation of more complex patterns of food intake relative to disease risk. In this large study in the WHI, we leveraged the well-characterized dietary data and an extensive array of blood biomarker assessment to examine the interrelationships with two recently established empirical hypothesis-oriented indices of dietary patterns: the EDIH as a pattern associated with dysfunctions in glucose and insulin homeostasis and EDIP, a pattern reflecting the ability of diet to contribute to chronic systemic inflammation (5). EDIH and EDIP were significantly associated with concentrations of 25 of the 40 biomarkers examined. We also observed that EDIH was more strongly related to biomarkers of insulin response, showed about equal magnitude of associations with biomarkers of inflammation and endothelial dysfunction, and showed weaker associations with lipids, compared to EDIP. Our findings provide additional insight into mechanisms linking dietary patterns to disease outcomes while also providing insights into where EDIH and EDIP show overlapping or distinctive impacts on relevant biomarkers.

EDIH and EDIP, as empirical hypothesis-oriented dietary indices, are unique and different than other dietary indices. The underlying hypothesis is that we can more precisely elucidate dietary patterns linked to relevant biomarkers and thus to disease risk, perhaps leading to more personalized dietary recommendations for those at risk. Given that the goal was to identify the combination of foods and beverages that optimize circulating concentrations of the relevant biomarkers, the resulting food combination may not always align with prevailing knowledge from studies of single foods or nutrients or from dietary patterns designed, using other dietary pattern methods, such as the Healthy Eating Index (28). Interestingly, EDIP and EDIH both contain wine, green-leafy vegetables, and full-fat dairy as favorable components to increase in the dietary pattern and low/non-fat dairy as an unfavorable component to reduce in the dietary pattern, in complex combinations with the other foods. Increasing the intake of green-leafy vegetables in proportion to all other vegetables could achieve the goal of optimal insulin response or circulating inflammatory markers. The fat content alone in the dairy products may not be as important as the whole dairy food regarding circulating levels of these biomarkers, e.g., although cheese and butter may have similar proportions of saturated fat, cheese has been associated with a more favorable biomarker profile than butter (29, 30). However, much remains to be learned regarding specific foods that may be mechanistically involved or could themselves simply be markers for other foods within a specific dietary pattern. Although, the empirical hypothesis-oriented patterns may not mimic patterns derived by other approaches, such as HEI or the Mediterranean pattern, the EDIP and EDIH warrant further investigation, especially in clinical trials, as they are robustly associated with risk of weight gain or incidence and mortality from several major chronic diseases, including type 2 diabetes (6–8), inflammatory bowel disease (31), digestive system cancer, including colorectal cancer (10–14), multiple myeloma (15), and prostate cancer (16, 17).

Hyperinsulinemia and insulin resistance, as well as chronic low-grade inflammation, both cluster within the pathogenesis of metabolic syndrome; therefore, it may not be surprising that the EDIH and EDIP, developed separately, share many food items and are demonstrating similar impacts on multiple biomarkers. The dietary indices generally have a moderate statistical correlation (Spearman *r* = 0.50–0.70), and higher scores of both dietary indices are associated with higher BMI and diets rich in red or processed meat, sugar-sweetened beverages, total fat and saturated fat, and reduced wine, tea/coffee, whole fruit, green-leafy vegetables, and total fiber. Yet EDIH and EDIP maintain some unique features. A hyperinsulinemic dietary pattern appears to be more strongly driven by the relative low intake of total fiber and, perhaps, has higher intake of total and saturated fat than a proinflammatory dietary pattern. We note that this is a nutrient profile that may not appear to align with the food components in these dietary patterns, especially in relation to fats, based on the prevailing knowledge base underlying the *a priori* dietary pattern approach. That is, the healthy or low insulinemic or the anti-inflammatory dietary pattern includes full-fat dairy foods yet is lower overall in total and saturated fats compared to hyperinsulinemic or proinflammatory dietary patterns that are high in low/non-fat dairy.

In the WHI cohort, we observed that, although EDIH showed stronger associations, both EDIH and EDIP were linked to elevations in the glucose/insulin biomarkers but with minimal association with the IGF system biomarkers. As would be hypothesized, the EDIH compared to EDIP, is more strongly associated with greater concentrations of glucose, insulin, C-peptide, HOMA-IR, and HOMA-β, yet we do see significant associations between dysregulated glucose homeostasis with higher EDIP scores, illustrating the pathophysiologic interface of hyperinsulinemic and proinflammatory dietary patterns. Interestingly, the dietary indices were generally not associated with IGF system biomarkers (IGF-1, free IGF-1, IGFBP-3, and IGFBP-4), except for the inverse association with IGFBP-1. Although greater IGF-1 bioactivity has been linked to the risk of carcinogenesis in laboratory (32), rodent (33), and some human studies (34–36), the regulation of the IGF system is likely related to dietary patterns unique from EDIH or EDIP or related to dietary patterns acting during earlier phases of the life course, for example, during periods of rapid growth (37–39). Indeed, components of the IGF system (IGF-1, IGFBP-3, etc.) are dependent on and positively correlated with growth hormone levels in children and adolescents (40). Therefore, the IGF system may be more important in early life events and less impacted by diet and lifestyle factors in adulthood. Also, insulin and IGF-1 concentrations are regulated differently and have very specific biological functions, although there may be overlap. Insulin and IGF-1 signal through their respective cell surface receptors (IR and IGF-1R), although the downstream-signaling pathways of the two receptors share components. The IR pathway is central to glucose homeostasis, while the IGF-1R pathway is crucial in mediating tissue growth in response to growth hormone (41), which may partly explain the differential associations between the dietary patterns and the insulin response biomarkers versus the IGF system biomarkers in the current study. However, the inverse association between both EDIH and EDIP with IGFBP-1 is intriguing, as lower IGFBP-1 has been associated with risk of insulin resistance, type 2 diabetes, obesity, and cardiovascular disease in human and rodent studies (42, 43). Additional studies on IGFBP-1 as impacted by EDIH and EDIP as a link to disease processes are warranted.

Among the 14 biomarkers of inflammation and endothelial dysfunction, EDIH performed similarly to or slightly better than EDIP. Both indices were predictive of β-cell function (HOMA-β) and insulin resistance (HOMA-IR), and human studies have linked insulin resistance with inflammatory signaling. The metabolic and inflammatory changes associated with EDIH and EDIP overlap and may share master regulators (44). Large prospective studies and meta-analyses have concluded that anti-TNF therapy improved hyperglycemia or insulin sensitivity and, importantly, reduced lifetime risk of diabetes (45). One study conducted among obese individuals without diabetes reported that prolonged (6 months) TNF inhibition significantly decreased fasting glucose and increased adiponectin, probably reflecting improved insulin sensitivity (46). Also, repeated observations that the treatment of inflammatory diseases such as psoriasis, rheumatoid arthritis, and Crohn's disease with TNF antagonists improves glycemia, provides additional hints that TNF may, indeed, have an important role in metabolic diseases (45). Other studies have shown that IL-6 has metabolic effects, which are pleotropic and context dependent (47). In the current study, EDIH was more strongly associated with IL-6, TNF-α, leptin, and ICAM-1 than EDIP; however, both dietary indices were strongly associated with type 2 diabetes prevalence in the current study and with type 2 diabetes risk in recent prospective cohort studies (7, 8), although with EDIP showing stronger associations than EDIH. The reasons for the stronger association of EDIP with type 2 diabetes are not entirely clear but could be related to its stronger associations with lipids.

It has been known for decades that nutrients, particularly circulating lipids, have a role in determining insulin sensitivity (48). Since then, it has become clear from human and animal studies that lipid-induced insulin resistance and impaired glucose metabolism may also involve other mechanisms, including the activation of inflammatory pathways (49, 50). In the current study, EDIP was more strongly associated with lipids including TG/HDL—a marker of insulin resistance (51), and lipid particle size than EDIH. Although previous studies have shown TG/HDL not to be a good marker of insulin resistance among African Americans (52), both dietary patterns were associated with TG/HDL among non-Hispanic white and African-African women but not among Hispanic women. Small-dense LDL particles have been associated with higher risk of metabolic syndrome, atherosclerosis, and CVD risk than large-size LDL particles (53, 54). Although EDIH was generally not associated with lipid particle size, EDIP was positively associated with medium and very small LDL particles and inversely associated with large LDL size and total LDL particle size. Among individuals with a proinflammatory constitution, such as in obesity, EDIH, and EDIP, dietary patterns may drive a vascular immunometabolic stress response within blood vessel endothelial and smooth muscle cells to promote vascular dysfunction and disease (55). For example, in vascular atherosclerosis, a disease process representing an interface between metabolism and a local inflammatory response, the plaques formed on the arterial walls are composed of lipids (cholesterol) and other substances, and the condition begins with dysfunction of the vascular endothelium, leading to higher concentrations of circulating inflammatory and endothelial adhesion molecules such as E-selectin, ICAM-1, and VCAM-1 (56). These vascular cell adhesion molecules respond to inflammatory cytokines and initiate the pathologic process of coronary artery disease and insulin resistance by forming atherosclerotic plaque and inhibiting insulin sensitivity and other biological functions. The consistently strong associations between the EDIH and EDIP and lipids, insulin response biomarkers, inflammatory and endothelial dysfunction markers in the current study could explain recent findings showing robust associations between higher EDIP scores and higher CVD (including coronary heart disease and stroke) (9).

Limitations of our study include potential measurement error in the FFQ (57, 58), although the WHI FFQ was evaluated for measurement characteristics prior to using it (20). Although we controlled for several confounding factors, the potential for confounding by unmeasured variables or residual confounding by inadequately measured variables may not be completely removed. Although we had a multiethnic sample, it was composed of postmenopausal women; therefore, future studies are warranted to examine these associations in the broader population of men and women over a wider age range. Although the sample sizes differed between biomarkers, the distribution of the exposures of interest (dietary indices) did not vary materially by outcome (biomarker) sample size, suggesting that differing biomarker sample sizes may not have induced selection bias. We could only conduct exploratory analyses in subgroups because of smaller sample sizes. Our study has several strengths as well, including the use of novel food-based empirical hypothesis-oriented dietary patterns in a well-characterized study population and use of a comprehensive set of circulating biomarkers for the construct validation of the dietary indices.

## Conclusion

In this large cohort of postmenopausal women in the United States, both hyperinsulinemic and proinflammatory dietary patterns, assessed using EDIH and EDIP scores, respectively, were associated with a broad range of circulating biomarkers of glucose-insulin dysregulation, chronic systemic inflammation, endothelial dysfunction, and dyslipidemia. Our findings further validate the EDIH and EDIP as dietary patterns reflective of broader chronic metabolic and inflammatory dysfunction associated with obesity and likely mediating a greater risk for type 2 diabetes, CVD, several cancers, and other disease processes. The future application of EDIH and EDIP scores in clinical and population-based studies to gain greater insight into dietary pattern and disease relationships is warranted. Most critically, the translation of low insulinemic (low-EDIH) and low inflammatory (low-EDIP) dietary patterns to future human clinical intervention trials may define strategies to reduce disease risk.

## Data Availability Statement

The original contributions presented in the study are included in the article/Supplementary Material, further inquiries can be directed to the corresponding author/s.

## Ethics Statement

The studies involving human participants were reviewed and approved by WHI. The WHI protocol was approved by the institutional review boards at the Clinical Coordinating Center at the Fred Hutchinson Cancer Research Center in Seattle, WA, and at each of the 40 Clinical Centers. The patients/participants provided their written informed consent to participate in this study.

## Author Contributions

DA and FT designed research. NS performed statistical analyses and drafted the manuscript. DA performed statistical analyses, and DA and QJ reviewed statistical programs for accuracy. DL, HH, XZ, JM, EL, AB, CA, SC, EG, and FT analyzed and interpreted the data and provided critical intellectual input. DA, NS, and FT had full access to all the data and take responsibility for the integrity of the data and the accuracy of the data analysis and results. FT provided overall study oversight. All the authors read, edited, and approved the final manuscript.

## Funding

The WHI program is funded by the National Heart, Lung, and Blood Institute, National Institutes of Health, U.S. Department of Health and Human Services through contracts HHSN268201600018C, HHSN268201600001C, HHSN268201600002C, HHSN268201600003C, and HHSN268201600004C.

## Conflict of Interest

The authors declare that the research was conducted in the absence of any commercial or financial relationships that could be construed as a potential conflict of interest.

## Publisher's Note

All claims expressed in this article are solely those of the authors and do not necessarily represent those of their affiliated organizations, or those of the publisher, the editors and the reviewers. Any product that may be evaluated in this article, or claim that may be made by its manufacturer, is not guaranteed or endorsed by the publisher.
