# Supplementary material for: Proinflammatory and Hyperinsulinemic Dietary Patterns Are Associated With Specific Profiles of Biomarkers Predictive of Chronic Inflammation, Glucose-Insulin Dysregulation, and Dyslipidemia in Postmenopausal Women
Source: Front Nutr. 2021 Sep 20;8:690428. doi: 10.3389/fnut.2021.690428 (PMC8488136; doi:10.3389/fnut.2021.690428)
Supplement: Supplementary file 1 [file Data_Sheet_1.pdf]

Online Supplemental Material

Number of Supplemental Tables: 10.

Number of other files (Appendix): 1.

Supplemental Table 1. Baseline characteristics of the study sample compared to excluded participants.

Supplemental Table 2. Food group components of the empirical dietary index for hyperinsulinemia (EDIH) score and empirical dietary inflammatory pattern (EDIP) score.

Supplemental Table 3. Biomarker assay description and quality control measures.

Supplemental Table 4. Baseline characteristics of the diabetes study sample in quartiles of dietary indices

Supplemental Table 5. Associations of EDIH and EDIP with biomarkers in body mass index subgroups

Supplemental Table 6. Associations of EDIH and EDIP with biomarkers in waist-to-hip ratio subgroups

Supplemental Table 7. Associations of EDIH and EDIP with biomarkers in race/ethnicity subgroups

Supplemental Table 8. Associations of EDIH and EDIP with biomarkers in categories of regular statin use

Supplemental Table 9. Associations of EDIH and EDIP with biomarkers in categories of regular NSAID use

Supplemental Table 10 Associations of EDIH and EDIP with biomarkers in categories of unopposed estrogen use.

Other files (Appendix): List of Women’s Health Initiative Investigators.

Supplemental Table 1. Baseline characteristics of the study sample compared to excluded participants

**Characteristic**[a]	**Included Sample (N=35,360)**	**Excluded Sample (N=26,246)**	**P-value**
Race/ethnicity[b], %			
Black or African-America	5026 (14.2)	6351 (24.2)	<0.0001
American Indian or Alaskan Native	321 (0.9)	326 (1.2)	
Hispanic/Latino	2324 (6.6)	2316 (8.8)	
Asian or Pacific Islander	1126 (3.2)	428 (1.6)	
White (not of Hispanic origin)	26260 (74.3)	16674 (63.5)	
Other race groups	255 (0.9)	124 (0.5)	
Age, years	64.4 ± 7.3	64.8 ± 7.3	0.3335
Body mass index (BMI), kg/m^2, %	27.0 ± 5.6	26.7 ± 9.5	<0.0001
Normal weight (15≤BMI<25)	14191 (40.1)	9935 (39.9)	<0.0001
Overweight (25≤BMI<30)	12114 (34.3)	6433 (25.8)	
Obese (BMI ≥30)	9052 (25.6)	8532 (34.3	
Physical activity, MET[d]-hours/week	12.9 ± 13.8	10.9 ± 12.9	<0.0001
Pack-years of smoking	9.9 ± 18.1	11.1 ± 20.0	<0.0001
Current Smoking, %	2870 (8.2)	2194 (8.5)	0.0073
Aspirin/NSAIDs[d] use, %	4871 (13.8)	3606 (13.7)	0.8462
Statin Use, %	794 (2.3)	688 (2.6)	0.0024
Educational level, %			
Less than high school	2160 (6.2)	2288 (8.8)	<0.0001
High school/GED[d]	19465 (55.5)	15064 (57.9)	
≥4 years of college	16459 (38.3)	8681 (33.3)	
Total alcohol intake, servings/week[5]	2.4 ± 4.9	1.9 ± 5.0	0.0055
Food intake[c], servings/week			
Red meat	3.3 ± 3.0	3.7 ± 4.2	<0.0001
Processed meat	1.8 ± 2.1	2.2 ± 3.0	<0.0001
Sugar-sweetened beverages	1.2 ± 3.4	1.5 ± 4.5	<0.0001
Refined grains	12.6 ± 7.6	12.6 ± 9.6	<0.0001
Wine	1.3 ± 3.2	0.9 ± 3.0	<0.0001
Tea/coffee	14.6 ± 12.3	13.4 ± 12.6	0.0004
Whole fruit	18.0 ± 12.2	17.8 ± 13.2	<0.0001
Green-leafy vegetables	6.0 ± 5.1	5.9 ± 5.2	<0.0001
Nutrient profile			
Total fiber, g/d	15.6 ± 6.7	15.7 ± 8.4	<0.0001
Total carbohydrate, g/d	15.6 ± 7.0	201 ± 111	<0.0001
Total protein, g/d	64.9 ± 26.0	68.5 ±42.0	<0.0001
Branched-chain amino acids, g/d	11.5 ± 4.8	12.2 ± 7.6	<0.0001
Total fat, g/d	56.7 ± 28.6	62.7 ± 47.1	<0.0001
Saturated fat, g/d	19.0 ± 10.3	20.8 ± 16.4	<0.0001

[a]Values presented are mean ± SD for continuous variables and percentages for categorical variables
[b]In WHI, race/ethnicity was self-identified. Users acknowledge that American Indian or Alaskan Native participants were self-identified and were primarily dwelling in urban areas and are not representative of the diverse American Indian population across the United States. Users agree not to use the data to infer tribal status or affiliation.
[c]The food group variables (servings/d) in the WHI were as follows: processed meat (hot dog, chorizo, other sausage, bacon, breakfast sausage, scrapple; lunch meat such as ham, turkey; other lunch meat such as bologna); red meat (ground meat including hamburgers, beef, pork, and lamb as a main dish or as a sandwich; stew, pot pie, and casseroles with meat; gravies made with meat drippings); refined grains (total grain variable minus whole grain variable, both WHI-computed food groups); Sugar-sweetened beverages all regular (not diet) soft drinks and fruit juice; wine (red wine, white wine); coffee or tea (all types); green leafy vegetables (cooked greens such as spinach, mustard greens, turnip greens, collards; lettuce and plain lettuce salad; mixed lettuce or spinach salad with vegetables);
[d]Abbreviation: MET, Metabolic equivalent of task; NSAID, Nonsteroidal anti-inflammatory drug; GED, General Educational Development

Supplemental Table 2. Food group components of the empirical dietary index for hyperinsulinemia (EDIH) score and empirical dietary inflammatory pattern (EDIP) score.

EDIH components[a]	Weight	Food items
		Food components positively associated with c-peptide concentrations
Processed meat	0.199	Processed meats (lunch meat other lunch meat), bacon, hot dog
Red meat	0.25	Beef, pork and lamb as a main dish, ground meat including hamburgers, Beef, pork, and lamb as a sandwich, stew, pot pie and casseroles with meat, gravies made with meat drippings, Menudo and tortilla soup
High-energy sugary beverages	0.104	Regular soft drinks (not diet)
Margarine	0.054	Margarine
Butter	0.094	Butter
French fries	0.581	French fries
Non-dark fish	0.172	Tuna, shrimp, lobster, scallops, seafood other than dark fish
Eggs	0.124	Egg
Low-fat dairy	0.025	Low-fat milk, sherbet or ice milk, yogurt, low-fat desserts
Cream soup	0.787	Chowder or cream soup
Tomatoes	0.095	Fresh tomato & tomato juice, tomato sauce
Poultry	0.183	Chicken & turkey, fried chicken, Chicken or turkey with or without skin
		Food components inversely associated with c-peptide concentrations
Green leafy vegetables	-0.055	Spinach& mustard greens& turnip greens& collards, iceberg or head lettuce, romaine or leaf lettuce
Wine	-0.165	Red, white wine
Coffee	-0.035	Coffee (regular or decaffeinated)
High-fat diary	-0.046	Whole milk, cream, sour cream ice cream, cream cheese, other cheese
Whole fruit	-0.029	Raisins, grapes, avocado, banana, cantaloupe, watermelon, orange, apple, pear, grapefruit, strawberries, blueberries, peaches, apricots, plums

EDIP components[b]	Weight	Food items
		Food components positively associated with inflammatory biomarkers

Processed meat	157.12082	Hot dogs, processed meats (including processed meat sandwich) , bacon
Red meat	135.78592	Hamburger, beef /pork /lamb sandwich, beef /pork/ lamb main dish
Organ meat	45.52817	Livers
Other fish	243.82936	Canned tuna, shrimp, breaded fish, lobster, scallops or other seafood
Other vegetables	136.89117	Corn, mixed vegetables, eggplant, celery, alfalfa sprouts, mushrooms, green/yellow/red peppers, zucchini, cucumbers
Refined grain	87.0247	White bread, white rice, bagels/English muffins/rolls, muffins or biscuits, pasta, pancakes or waffles, refined cold breakfast cereals
High energy beverage	154.80026	Cola, Hawaiian punch, caffeine-free coke, pepsi, carbonated beverage with caffeine and sugar, other carbonated beverage with sugar
Tomato	160.65882	Fresh tomatoes, tomato juice, tomato sauce
		Food components inversely associated with inflammatory biomarkers
Beer	-135.2397	Beer, light beer
Wine	-248.8155	White wine, red wine
Tea	-128.297	Tea, tea (not herbal)
Coffee	-128.297	Coffee, decaffeinated coffee
Dark yellow vegetable	-166.1962	Carrots, sweet potatoes, winter squash
Green leafy vegetable	-188.9353	Spinach, iceberg lettuce, romaine lettuce
Snack	-43.82505	Potato/corn chips, popcorn, crackers
Fruit juice	-60.66021	Apple juice, orange juice, grape juice, prune juice, other juice
Pizza	-1169.052	Pizza

[a]The EDIH component foods (servings/d) in the WHI were: Red meat (ground meat including hamburgers, beef, pork and lamb as a main dish, or as a sandwich; stew, pot pie and casseroles with meat; gravies made with meat drippings); high-energy sugary beverages, (all regular - not diet - soft drinks); low-energy sugary beverages (the WHI FFQ did not assess low-energy beverages separately from other sugar-sweetened beverages); cream soup (such as chowders, potato, tomato, cheese, ajiaco); processed meat (hot dogs, chorizo; other sausage, bacon, breakfast sausage, scrapple; lunch meat such as ham, turkey; other lunch meat such as bologna); butter, margarine (butter, margarine or oil, on bread or tortillas; margarine or butter added to cooked cereal or grits; butter, margarine, sour cream, oils, or other fat added to vegetables, beans, rice, and potatoes, after cooking); poultry (poultry); French fries (French fries, fried potatoes, fried rice, fried cassava and fritters); non-dark or non-oily fish (fried fish, shrimp, lobster, crab and oysters, canned tuna, tuna salad, and tuna casserole, white fish such as sole, snapper, cod); tomatoes (fresh tomato, tomato juice, tomato sauce, cooked tomato, salsa and salsa picante); low-fat dairy (part-skim or reduced fat cheeses, such as Mexican-type cheeses or mozzarella. Include cheese added to foods and in cooking; low-fat cottage cheese; low-fat or no-fat frozen desserts, such as frozen yogurt, sherbet, ice milk, and low-fat milkshakes; non-fat yogurt (not frozen); all other yogurt (not frozen); low-fat milk; Milk, cream, or creamer in coffee or tea); eggs (eggs); wine (red wine, white wine); coffee or tea (all types); fruits (all types); high-fat dairy (whole milk, evaporated/condense milk, ice cream, cottage cheese and ricotta cheese, other cheese); green leafy vegetables (cooked greens such as spinach, mustard greens, turnip greens, collards; lettuce and plain lettuce salad; mixed lettuce or spinach salad with vegetables).

[b]The EDIP component foods (servings/d) in the WHI were: processed meat (hot dogs, chorizo, other sausage, bacon, breakfast sausage, scrapple; lunch meat such as ham, turkey; other lunch meat such as bologna); red meat (ground meat including hamburgers, beef, pork, and lamb as a main dish or as a sandwich; stew, pot pie, and casseroles with meat;gravies made with meat drippings); organ meat (liver, including chicken liver; other organ meats); fish other than dark-meat fish (fried fish, shrimp, lobster, crab and oysters, canned tuna, tuna salad, and tuna casserole, white fish such as sole, snapper, cod); other vegetables (i.e., vegetables other than green leafy vegetables and dark yellow vegetables: red peppers and red chilies, green peppers, green chilies, jalapenos, and green chili salsa, corn, and hominy); refined grains (total grain variable minus whole grain variable, both WHI-computed food groups); high-energy beverages [all regular (not diet) soft drinks]; low-energy beverages (the WHI FFQ did not assess low-energy beverages); tomatoes (fresh tomato, tomato juice, tomato sauce, cooked tomato, salsa and salsa picante); beer (all types); wine (red wine, white wine); coffee or tea (all types); dark-yellow vegetables (carrots, including mixed dishes with carrots; summer squash, zucchini, nopales, and okra; winter squash, such as acorn, butternut, and pumpkin; sweet potatoes and yams; other potatoes, cassava, and yucca—boiled, baked, or mashed); green leafy vegetables (cooked greens such as spinach, mustard greens, turnip greens, collards; lettuce and plain lettuce salad; mixed lettuce or spinach salad with vegetables); pizza (low-fat pizza; other pizza); fruit juice (orange juice and grapefruit juice; other fruit juices such as apple and grape); snacks (snacks such as potato chips, corn chips, tortilla chips, Ritz and cheese crackers; saltines, Snackwell’s, fat-free tortilla chips and fat-free potato chips; popcorn).

Supplemental Table 3. Biomarker assay description and quality control measures

Biomarker	Sample size	# of labs that assessed biomarker	Mean coefficient of variation (CV, %)	CV range	Measurement Method	Specimen Type
Glucose	21,669	7	1.9	1.9 - 3.5	Hexokinase method	Serum
Insulin	23,756	15	7.0	2.3 - 14.5	RIA/ELISA/EI	Serum, plasma
C-peptide	943	3	6.2	2.7 - 12.8	RIA/ELISA	Serum, plasma
Insulin growth factor (IGF)-1	3,126	8	15.0	2.3 - 33.8	ELISA/RIA/Cytokine array/NMR	Serum, plasma
IGF binding protein-1	993	5	4.1	1.9 - 9.4	RIA/ELSIA/IRMA	Serum, plasma
IGF binding protein-3	2,349	7	7.0	2.2 - 21.5	RIA/ELSIA/IRMA	Serum, plasma
IGF binding protein-4	354	1	16.0	NA	ELISA	Plasma
Free IGF-1	2,203	2	15.0	16.4 - 19.5	ELISA	Serum
C-reactive protein (high sensitivity)	26,482	20	4.6	0.9 - 10.8	Nephelometry/XRF/MS/IT	Serum, plasma
serum Amyloid A	1,181	3	9.8	1.2 - 23.4	latex-enhanced nephelometry/cytokine array	Serum
Interleukin 6	12,408	11	19.3	4.0 - 41.6	ELISA/ cytokine array	Serum, plasma
Interleukin 10	2,466	6	32.4	22.0 - 65.0	ELISA/ cytokine array	Serum, plasma
Tumor necrosis factor Alpha	5,302	8	22.0	14.6 - 30.7	ELISA/ cytokine array	Serum, plasma
Tumor necrosis factor receptor 1	3,908	3	7.9	6.2 - 10.6	ELISA/ cytokine array	Serum, plasma
Tumor necrosis factor receptor 2	7,746	7	7.1	4.6 - 11.0	ELISA/ cytokine array	Serum, plasma
Adiponectin	7,552	11	9.7	5.4 - 14.1	ELISA/ cytokine array	Serum, plasma
Leptin	8,045	7	6.9	1.5 - 12.8	RIA/cytokine array	Serum, plasma
VEGF	873	3	14.8	7.8 - 28.0	Cytokine array	Serum
E-Selectin	3,817	3	9.7	7.1 - 14.3	ELISA	Plasma
VCAM-1	4,050	5	6.6	4.9 - 9.1	ELISA/ cytokine array	Serum, plasma
ICAM-1	1,075	3	8.0	3.9 - 12.2	ELISA/ cytokine array	Serum, plasma
GCSF	1,413	4	18.7	11.7 - 26.5	Cytokine array	Serum, plasma
Hemoglobin A1c	570	1	2.1	NA	IT	RBC

Total Cholesterol	21,378	8	1.8	1.1 - 3.8	Enzymatic method	Serum, plasma
Triglyceride	18,833	10	2.9	1.6 - 4.2	Enzymatic colorimetric	Serum, plasma
High-density lipoprotein cholesterol	20,508	9	4.0	2.3 - 6.1	Precipitation/NMR	Serum, plasma
Low-density lipoprotein cholesterol	16,525	8	3.8	1.8 - 8.6	Calculation using Friedewald formula	Serum, plasma
Large LDL Particles	1,653	2	18.9	14.9 - 22.9	NMR	Serum, plasma
Medium Small LDL Particles	1,356	1	16.8	NA	NMR	plasma
Small LDL Particles Total	1,653	2	19.0	17.7 - 20.2	NMR	Serum, plasma
Very Small LDL Particles	1,356	1	16.3	NA	NMR	Plasma
Total size of all LDL	1,652	2	0.9	0.89 - 0.94	NMR	Serum, plasma
Intermediate density lipoprotein	1,653	2	32.5	28.7 - 36.3	NMR	Serum, plasma
Large HDL Particles	1,653	2	13.7	12.7 - 14.6	NMR	Serum, plasma
Medium HDL Particles	1,241	2	33.2	23.1 - 43.3	NMR	Serum, plasma
Small HDL Particles	1,653	2	10.5	6.5 - 14.4	NMR	Serum, plasma
HDL Size	1,653	2	1.0	0.8 - 1.01	NMR	Serum, plasma

Abbreviations: RIA: radioimmunoassay EI, electrochemiluminescence immunoassay; ELISA, enzyme-linked, NA, not available; immunosorbent assay; IT: Immunoturbidimetric assay; XRF: X-ray fluorescence; MS: Mass spectrometry; NMR: Nuclear Magnetic Resonance; VEGF, Vascular endothelial growth factor; VCAM-1, vascular cell adhesion protein 1; ICAM-1, intercellular adhesion molecule 1; GCSF, Granulocyte colony-stimulating factor; RBC: red blood cell;

Supplemental Table 4. Baseline characteristics of the diabetes study sample in quartiles of dietary indices

	Empirical Dietary Index for Hyperinsulinemia (EDIH) score[b]				Empirical Dietary Inflammatory Index (EDIP) score			
**Characteristic[a]**	Quartile 1 (n=142)	Quartile 2 (n=143)	Quartile 3 (n=143)	Quartile 4 (n=142)	Quartile 1 (n=142)	Quartile 2 (n=143)	Quartile 3 (n=143)	Quartile 4 (n=142)
Race/ethnicity[c], %								
Black or African-American	7 (5)	9 (6)	16 (11)	20 (14)	4 (3)	6 (4)	17 (12)	25 (18)
American Indian or Alaskan Native	2 (1)	1(1)	1(1)	2 (1)	2 (1)	1(1)	2 (1)	1(1)
Hispanic/Latino	1 (1)	5(3)	2(1)	5 (4)	1(1)	3 (2)	2 (1)	7 (5)
Asian or Pacific Islander	3 (2)	9 (6)	9 (6)	9 (6)	1(1)	4 (3)	11 (8)	14 (10)
White (not of Hispanic origin)	129 (90)	117 (82)	114 (80)	104 (73)	132 (93)	127 (88)	110 (77)	94 (66)
Other race groups	1 (1)	2 (1)	1(1)	2 (1)	2 (1)	2 (1)	1(1)	1(1)
Age, years	65.9 ± 7.2	66.5 ± 7.0	66.5 ± 7.2	64.9 ± 7.7	66.1 ± 7.2	65.7 ± 7.3	66.9 ± 6.9	65.1 ± 7.7
Body mass index (BMI), kg/m^2, %	26.8 ± 5.2	26.7 ± 5.0	27.2 ± 5.6	29.9 ± 6.2	26.8 ± 5.5	27.8 ± 5.2	27.1 ± 5.8	28.9 ± 6.3
Underweight (15≤BMI<18.4)	2 (1)	2 (1)	2 (1)	1(1)	1(1)	1(1)	5 (4)	0 (0)
Normal weight (18.5≤BMI<25)	64 (46)	60 (42)	56 (39)	32 (23)	62 (45)	53 (37)	51 (36)	46 (33)
Overweight (25≤BMI<30)	49 (35)	43 (30)	47 (33)	39 (28)	47 (34)	43 (30)	54 (38)	34 (24)
Obese (BMI ≥30)	24 (17)	37 (26)	37 (260	68 (49)	29 (21)	45 (32)	32 (22)	60 (43)
Physical activity, MET[e]-hours/week	16.2 ± 15.5	15.3 ± 16.0	12.2 ± 13.3	9.6 ± 12.1	16.5 ± 15.4	13.4 ± 13.1	12.9 ± 12.9	10.5 ± 15.9
Pack-years of smoking	14.1 ± 23.5	8.6 ± 15.4	9.6 ± 18.5	12.7 ± 22.7	13.6 ± 21.1	10.7 ± 17.1	10.3 ± 22.1	10.4 ± 21.0
Current Smoking, %	5 (4)	9 (6)	9 (6)	18 (13)	12 (9)	9 (10)	7 (5)	13 (9)
Aspirin/NSAIDs[e] use, %	20 (14)	14 (10)	19 (13)	18 (13)	18 (13)	19 (13)	15 (11)	19 (13)
Statin Use, %	3 (2)	6 (4)	4 (3)	4 (3)	5 (4)	6 (4)	4 (3)	2 (1)
Educational level, %								
Less than high school	9 (6)	6 (4)	9 (6)	14 (10)	5 (4)	7 (5)	8 (5)	17 (12)
High school/GED[e]	63 (44)	71 (50)	88 (61)	91 (63)	68 (48)	77 (54)	81 (57)	86 (61)
≥4 years of college	70 (49)	66 (46)	47 (33)	38 (27)	66 (48)	59 (41)	54 (38)	38 (27)

Total alcohol intake, servings/week	3.9 ± 5.5	2.5 ± 5.4	2.1 ± 4.2	1.6 ± 3.5	4.1 ± 6.4	3.5 ± 5.3	1.7 ± 3.5	0.8 ± 1.8
Food intake[d], servings/week								
Red meat	2.0 ± 2.0	2.3 ± 2.1	2.8 ± 2.2	6.1 ± 4.3	2.8 ± 2.9	3.1 ± 2.9	3.0 ± 2.7	4.3 ± 4.1
Processed meat	1.1 ± 1.6	1.4 ± 1.6	1.7 ± 1.7	3.1 ± 3.0	1.6 ± 2.0	1.7 ± 3.1	1.8 ± 2.1	2.3 ± 2.5
Sugar-sweetened beverages	0.1 ± 0.8	0.3 ± 0.9	0.7 ± 2.3	1.7 ± 3.5	0.2 ± 0.9	0.5 ± 2.1	0.5 ± 2.1	1.5 ± 3.1
Refined grains	14.5 ± 8.2	11.6 ± 5.7	10.1 ± 6.3	11.7 ± 7.7	12.2 ± 6.6	10.9 ± 6.6	11.3 ± 6.0	13.5 ± 9.0
Wine	2.8 ± 4.5	1.2 ± 2.1	0.8 ± 2.1	0.5 ± 1.1	2.7 ± 4.4	1.7 ± 2.7	0.6 ± 1.6	0.2 ± 0.9
Tea/coffee	21.9 ± 15.3	14.7 ± 13.3	10.9 ± 7.3	10.3 ± 9.3	27.4 ± 15.1	13.9 ± 8.3	10.7 ± 6.2	5.9 ± 6.6
Whole fruit	24.6 ± 16.6	19.8 ± 11.3	15.9 ± 9.8	14.9 ± 9.7	21.8± 13.0	18.3 ± 11.4	18.9 ± 13.1	16.1 ± 12.7
Green-leafy vegetables	8.0 ± 6.2	6.2 ± 4.3	5.3 ± 4.9	5.1 ± 4.1	9.3 ± 6.5	6.6 ± 4.5	5.4 ± 3.6	3.3 ± 3.0
Nutrient profile								
Total fiber, g/d	20.3 ± 7.6	16.2 ± 5.8	15.0 ± 6.4	14.2 ± 6.4	18.5 ± 7.2	16.7 ± 6.8	16.0 ± 6.8	14.5 ± 6.6
Total carbohydrate, g/d	236.5 ± 77.1	186.0 ± 57.8	168.7 ± 58.6	181.6 ± 70.4	211.0 ± 67.5	189.0 ± 72.4	187.6 ± 71.1	185.2 ± 71.0
Total protein, g/d	73.3 ± 27.1	59.6 ± 23.1	57.0 ± 21.6	75.5 ± 30.1	70.4 ± 25.2	66.5 ± 27.0	62.5 ± 26.5	65.9 ± 28.3
Branched-chain amino acids, g/d	13.1 ± 5.1	10.6 ± 4.2	10.1 ± 4.0	13.4 ± 5.4	12.5 ± 4.7	11.8 ± 4.9	11.1 ± 4.9	11.7 ± 5.1
Total fat, g/d	52.9 ± 26.6	44.8 ± 20.9	48.8 ± 22.9	71.9 ± 34.8	53.7 ± 26.0	54.7 ± 30.3	51.5 ± 24.4	58.5 ± 33.1
Saturated fat, g/d	17.8 ± 9.4	14.6 ± 7.5	15.9 ± 8.2	23.7 ± 11.9	17.7 ± 9.3	18.0 ± 10.2	16.9 ± 8.8	19.4 ± 11.9

[a]Values presented are mean ± SD for continuous variables and percentages for categorical variables.
[b]Dietary indices were adjusted for total energy intake.
[c]In WHI, race/ethnicity was self-identified. Users acknowledge that American Indian or Alaskan Native participants were self-identified and were primarily dwelling in urban areas and are not representative of the diverse American Indian population across the United States. Users agree not to use the data to infer tribal status or affiliation.
[d]The food group variables (servings/d) in the WHI were as follows: processed meat (hot dog, chorizo, other sausage, bacon, breakfast sausage, scrapple; lunch meat such as ham, turkey; other lunch meat such as bologna); red meat (ground meat including hamburgers, beef, pork, and lamb as a main dish or as a sandwich; stew, pot pie, and casseroles with meat; gravies made with meat drippings); refined grains (total grain variable minus whole grain variable, both WHI-computed food groups); Sugar-sweetened beverages all regular (not diet) soft drinks and fruit juice; wine (red wine, white wine); coffee or tea (all types); green leafy vegetables (cooked greens such as spinach, mustard greens, turnip greens, collards; lettuce and plain lettuce salad; mixed lettuce or spinach salad with vegetables).
[e]Abbreviation: MET, Metabolic equivalent of task; NSAID, Nonsteroidal anti-inflammatory drug; GED, General Educational Development

Supplemental Table 5. Associations of EDIH and EDIP with biomarkers in body mass index subgroups

Biomarkers	Empirical Dietary Index for Hyperinsulinemia (EDIH) score							Empirical Dietary Inflammatory Index (EDIP) score						
	Normal weight[a] (18.5, 24.9)		Overweight (25, 29.9)		Obese (30, 50)		FDR P-interaction	Normal weight (18.5, 24.9)		Overweight (25, 29.9)		Obese (30, 50)		FDR P-interaction
	Percentage Difference[b,c]	FDR p value	Percentage Difference	FDR p value	Percentage Difference	FDR p value		Percentage Difference	FDR p value	Percentage Difference	FDR p value	Percentage Difference	FDR p value	
**Insulin and IGF pathway biomarkers**														
Glucose, mg/dL (n=21,669)	**1.2 (0.8, 1.5)**	**9.83E-11**	**1.1 (0.7, 1.5)**	**4.24E-08**	**1.4 (0.9, 1.8)**	**3.16E-08**	0.9624	**0.5 (0.2, 0.8)**	**0.0140**	**0.9 (0.5, 1.3)**	**1.48E-05**	**1.0 (0.5, 1.4)**	**0.0004**	0.9640
Insulin, ng/mL (n=23,756)	**5.9 (4.3, 7.5)**	**1.47E-11**	**5.5 (4.0, 6.9)**	**2.52E-12**	**5.7 (4.2, 7.1)**	**3.94E-13**	**3.41E-05**	**3.5 (2.0, 5.0)**	**5.31E-05**	**5.2 (3.8, 6.6)**	**3.06E-11**	**5.4 (3.9, 6.9)**	**3.17E-11**	0.1617
C-peptide, ng/mL (n=943)	4.2 (-0.9, 9.2)	0.2834	4.1 (-1.9, 10.2)	0.3789	5.7 (-0.2, 11.6)	0.1850	0.9964	1.5 (-3.1, 6.1)	0.6907	0.3 (-5.4, 6.0)	0.9282	6.4 (-0.9, 13.6)	0.2082	0.9573
HOMA-IR[d] (n=19,865)	**7.2 (5.6, 8.9)**	**4.02E-16**	**6.7 (5.4, 8.4)**	**1.45E-16**	**6.9 (5.3, 8.5)**	**4.02E-16**	**1.00E-04**	**4.1 (2.5, 5.7)**	**4.70E-06**	**6.5 (4.9, 8.0)**	**9.71E-15**	**6.5 (4.8, 8.1)**	**6.42E-13**	0.3220
HOMA-B[d] (n=19865)	**2.5 (0.9, 4.0)**	**9.93E-03**	**2.6 (1.2, 4.0)**	**0.0023**	**2.6 (1.2, 4.1)**	**0.0027**	**0.0007**	**2.1 (0.6, 3.6)**	**0.0195**	**2.9 (1.5, 4.3)**	**0.0003**	**3.7 (2.2, 5.2)**	**1.17E-05**	0.5191
IGF[d]-1, pg/mL (n=3,126)	-2.6 (-7.4, 2.1)	0.4865	1.1 (-6.0, 3.9)	0.8277	2.1 (-2.6, 6.9)	0.5970	0.7677	-1.9 (-6.2, 2.4)	0.5768	0.9 (-3.8, 5.7)	0.8013	3.0 (-2.0, 8.0)	0.4400	0.4402
IGFBP[d]1, pg/L (n= 993)	**-7.9 (-14.0, -1.8)**	**0.0504**	**-9.1 (-17.9, -0.2)**	0.1452	-9.8 (-23.3, 3.8)	0.3551	0.7437	-2.6 (-8.7,3.4)	0.5768	-3.8 (-12.6, 5.0)	0.5768	-9.1 (-25.9, 7.7)	0.4928	0.9160
IGFBP3, ng/mL (n=2,349)	-0.7 (-3.6, 2.1)	0.8209	0.5 (-2.8, 3.9)	0.8879	-0.3 (-4.0, 3.3)	0.9115	0.9532	0.4 (-2.2, 3.0)	0.8524	3.1 (-0.2, 6.3)	0.1777	-0.8 (-4.4, 2.8)	0.7782	0.4273
IGFBP4, ng/mL (n=354)	7.6 (-2.5, 17.6)	0.3321	-2.1 (-10.6, 6.4)	0.8209	0.1 (-7.7, 7.8)	0.9812	0.6332	6.7 (-2.4, 15.7)	0.3023	4.9 (-3.8, 13.5)	0.4686	-3.9 (-13.4, 5.6)	0.5958	**0.0943**
free IGF-1, pg/mL (n=2,203)	-4.2 (-11.0, 2.6)	0.4443	0.7 (-6.3, 7.7)	0.9115	8.2 (0.6, 15.9)	0.1176	0.8543	-4.9 (-11.2, 1.4)	0.2715	-1.7 (-8.4, 5.0)	0.7492	2.1 (-5.5, 9.6)	0.7315	0.8691
**Inflammation and epithelial dysfunction biomarkers**														
C-reactive protein, mg/L (n=26,482)	**9.8 (7.5, 11.9)**	**3.66E-16**	**6.4 (4.5, 8.3)**	**5.00E-10**	**4.5 (2.7, 6.3)**	**8.38E-06**	**3.20E-18**	**7.7 (5.6, 9.8)**	**1.94E-11**	**6.6 (4.8, 8.5)**	**5.96E-11**	**4.8 (3.0, 6.7)**	**2.96E-06**	**9.55E-09**
Serum Amyloid A, mg/L (n=1,181)	4.1 (-4.5, 12.7)	0.5869	5.4 (-2.6, 13.5)	0.3951	0.7 (-9.1, 10.4)	0.9336	0.8311	6.8 (-0.9, 14.6)	0.2082	-1.9 (-9.0, 5.2)	0.7315	7.4 (-2.0, 16.9)	0.2715	0.8353
Interleukin-6, pg/mL (n=12,408)	**7.4 (4.2, 10.7)**	**6.72E-05**	**5.9 (3.0, 8.7)**	**5.95E-04**	**3.8 (0.9, 6.8)**	**0.0504**	0.4036	**4.9 (1.9, 8.0)**	**0.0062**	**3.7 (0.9, 6.5)**	**0.0323**	**4.0 (1.0, 7.6)**	**0.0330**	0.8691
Interleukin 10, pg/L (n=2,466)	-0.5 (-7.2, 6.3)	0.9336	**-7.7 (-14.3, -1.1)**	**0.0798**	-1.4 (-8.0, 5.3)	0.8301	0.9888	-0.9 (-7.3, 5.4)	0.8524	**-6.5 (-12.3, -0.7)**	**0.0844**	-4.0 (-10.7, 2.8)	0.4527	0.9964
TNF[d] alpha, mg/mL (n=5,302)	**7.5 (1.0, 14.0)**	**0.0856**	5.5 (-0.9, 11.9)	0.2583	6.9 (-0.8, 14.7)	0.2384	0.6717	0.8 (-5.5, 7.2)	0.8712	3.3 (-2.7, 9.2)	0.4928	8.1 (0.6, 15.7)	0.1003	0.9964
TNF alpha receptor 1, pg/mL (n=3,908)	0.7 (-1.0, 2.5)	0.6329	0.6 (-1.0, 2.1)	0.7066	0.4 (-1.8, 2.5)	0.8823	0.7913	1.0 (-0.6, 2.5)	0.4345	1.0 (-0.5, 2.4)	0.4070	0.5 (-1.6, 2.6)	0.7610	0.7952
TNF alpha receptor 2, pg/mL (n=7,746)	1.2 (-0.4, 2.7)	0.3309	0.9 (-0.6, 2.3)	0.4447	1.2 (-0.4, 2.8)	0.3500	0.8543	**1.8 (0.4, 3.2)**	**0.0419**	1.1 (-0.3, 2.5)	0.2659	**2.8 (1.1, 4.4)**	**0.0041**	0.7345

Adiponectin, ng/mL (n=7552)	-3.0 (-6.1, 0.004)	0.1587	-0.8 (-3.7, 2.0)	0.7871	-2.7 (-6.1, 0.6)	0.2917	0.7913	**-4.1 (-7.0, -1.3)**	**0.0171**	-2.0 (-4.8, 0.8)	0.3154	-1.8 (-5.2, 1.6)	0.5066	0.3220
Leptin, ng/mL (n=8,045)	**7.8 (5.1, 10.6)**	**3.23E-07**	**2.8 (0.6, 5.1)**	**0.0533**	**3.1 (0.8, 5.4)**	**0.0426**	**2.40E-18**	**5.6 (3.0, 8.1)**	**0.0001**	0.9 (-1.2, 3.0)	0.5768	1.7 (-0.6, 4.0)	0.2965	**2.40E-18**
VEGF[d], pg.ml (n=873)	-2.5 (-10.8, 5.9)	0.7871	-2.2 (-11.3, 7.0)	0.8211	-4.2 (-16.8, 8.4)	0.7360	0.8543	-2.9 (-11.1, 5.3)	0.6686	-3.0 (-11.1, 5.1)	0.6580	1.1 (-11.3, 13.5)	0.9060	0.8606
E-selectin, ng/ml (n=3,817)	2.5 (-0.6, 5.6)	0.3028	1.5 (-1.5, 4.5)	0.5482	**3.8 (1.0, 6.7)**	**0.0426**	0.8543	1.6 (-1.4, 4.6)	0.5057	2.8 (-0.1, 5.7)	0.1512	**4.8 (1.9, 7.8)**	**0.0057**	0.6934
VCAM[d]-1, ng.ml (n=4,050)	0.5 (-1.5, 2.5)	0.8209	-1.6 (-3.5, 0.3)	0.2786	**2.5 (0.4, 4.5)**	**0.0744**	0.2770	0.6 (-1.3, 2.5)	0.6907	-1.1 (-2.9, 0.8)	0.4527	**4.8 (2.7, 6.8)**	**3.23E-05**	0.1713
ICAM[d]-1, ng/ml (n=1,075)	3.0 (-1.6, 7.5)	0.4175	**5.4 (0.8, 10.0)**	**0.0798**	**6.9 (1.1, 12.6)**	**0.0744**	0.8126	3.4 (-0.9, 7.7)	0.2715	1.3 (-2.8, 5.5)	0.6907	**6.2 (0.4, 11.9)**	**0.1003**	0.8543
GCSF, pg/ml (n=2,160)	0.8 (-6.2, 7.7)	0.9115	1.6 (-5.6, 8.7)	0.8277	3.9 (-2.8, 10.5)	0.4649	0.9532	0.5 (-6.0, 7.1)	0.9151	-0.7 (-7.1, 5.7)	0.8918	1.1 (-5.7, 7.8)	0.8524	0.8543
**Lipid and lipid particle biomarkers**														
Total cholesterol, mg/dL (n=21,378)	0.3 (-0.2, 0.8)	0.4354	0.2 (-0.3, 0.6)	0.6492	0.3 (-0.2, 0.7)	0.4700	0.1085	0.1 (-0.3, 0.6)	0.7315	0.2 (-0.2, 0.6)	0.5656	-0.1 (-0.6, 0.4)	0.7598	**0.0516**
Triglycerides, mg/dL (n=18,833)	0.9 (-0.4, 2.2)	0.3743	**1.7 (0.5, 2.9)**	**0.0303**	**2.1 (0.9, 3.3)**	**0.0031**	**0.0170**	**3.0 (1.7, 4.2)**	**2.06E-05**	**3.7 (2.5, 4.9)**	**2.13E-08**	**2.4 (1.2, 3.6)**	**0.0007**	**8.76E-06**
High density Lipoprotein (HDL), mg/dL (n=20,508)	-0.6 (-1.3, 0.1)	0.2583	-0.2 (-0.9, 0.4)	0.6492	-0.5 (-1.1, 0.1)	0.2917	**0.0007**	**-1.5 (-2.2, -0.9)**	**5.31E-05**	**-1.3 (-1.9, -0.7)**	**0.0001**	**-0.9 (-1.5, -0.3)**	**0.0182**	**8.76E-06**
Low density Lipoprotein (LDL), mg/dL (n=16,525)	0.6 (-0.2, 1.5)	0.3321	0.3 (-0.5, 1.0)	0.7003	0.4 (-0.4, 1.1)	0.5713	0.1085	0.6 (-0.2, 1.4)	0.3023	0.1 (-0.7, 0.8)	0.9045	-0.4 (-1.1, 0.4)	0.5324	**0.0162**
Triglycerides/High density Lipoprotein (n=17,761)	**2.3 (0.4, 4.1)**	**0.0622**	**1.8 (0.1, 3.5)**	0.1119	**2.8 (1.2, 4.4)**	**0.0043**	**0.0009**	**4.8 (3.1, 6.6)**	**1.02E-06**	**4.5 (2.8, 6.1)**	**1.18E-06**	**3.1 (1.4, 4.8)**	**0.0012**	**6.53E-08**
Triglycerides/Total cholesterol (n=18,631)	0.6 (-0.7, 1.8)	0.5970	**1.6 (0.5, 2.8)**	**0.0316**	**1.8 (0.7, 2.9)**	**0.0099**	0.1237	**2.7 (1.5, 3.8)**	**5.31E-05**	**3.6 (2.5, 4.7)**	**7.94E-09**	**2.4 (1.3, 3.6)**	**0.0002**	**0.0003**
Large LDL, nmol/L (n=1,653)	1.0 (-3.0, 5.0)	0.8209	-1.8 (-5.5, 2.0)	0.5879	-2.3 (-7.4, 2.8)	0.5970	0.6273	-0.5 (-4.2, 3.1)	0.8524	-3.9 (-7.5, -0.3)	**0.1003**	-1.6 (-6.5, 3.3)	0.6907	0.9874
Medium LDL, nmol/L (n=1,356)	-0.9 (-6.9, 5.2)	0.9015	3.3 (-2.2, 8.8)	0.4447	1.6 (-5.3, 8.5)	0.8211	0.4036	2.2 (-3.6, 8.0)	0.6518	4.4 (-0.8, 9.6)	0.2343	3.3 (-3.1, 9.7)	0.5090	0.6044
Small LDL, nmol/L (n=1,653)	1.3 (-4.7, 7.2)	0.8277	1.3 (-3.9, 6.5)	0.8209	-0.8 (-7.3, 5.6)	0.9015	0.2735	3.6 (-1.9, 9.1)	0.4027	3.2 (-1.8, 8.1)	0.4070	0.3 (-5.8, 6.5)	0.9282	0.4036
Very small LDL, nmol/L (n=1,356)	0.8 (-5.1, 6.7)	0.9015	3.4 (-1.8, 8.7)	0.4147	1.9 (-5.0, 8.8)	0.8099	0.4718	2.7 (-3.0, 8.4)	0.5536	4.0 (-1.0, 9.0)	0.2659	3.3 (-3.1, 9.6)	0.5090	0.6738
Total size of all LDL, nm (n=1,652)	-0.03 (-0.3, 0.3)	0.9115	-0.2 (-0.5, 0.1)	0.3421	-0.2 (-0.6, 0.2)	0.5217	0.9837	-0.1 (-0.4, 02)	0.7625	-0.3 (-0.6, -0.05)	**0.0646**	-1.9 (-0.5, 0.2)	0.5090	0.8691
Intermediate density lipoprotein, nmol/L (n=1,653)	0.2 (-9.5, 9.9)	0.9777	2.8 (-5.7, 11.3)	0.7360	0.8 (-8.0, 9.6)	0.9115	0.7140	-2.5 (-11.5, 6.4)	0.7315	0.3 (-73-.8, 8.5)	0.9418	10.3 (1.9, 18.8)	**0.0502**	0.8207
Large HDL, nmol/L (n=1,653)	0.8 (-4.2, 5.8)	0.8823	0.6 (-4.2, 5.3)	0.9115	1.5 (-5.1, 8.2)	0.8211	0.6545	-0.5 (-5.2, 4.1)	0.8828	-0.3 (-4.8, 4.2)	0.9229	-0.2 (-6.7, 6.2)	0.9418	0.6659
Medium HDL, nmol/L (n=1,241)	10.5 (-5.9, 27.0)	0.4175	-0.4 (-15.6, 14.7)	0.9710	1.0 (-15.4, 17.5)	0.9353	0.5926	-3.2 (-18.3, 11.9)	0.7820	-4.5 (-19.0, 10.1)	0.7054	14.8 (-1.5, 31.1)	0.1913	0.9930
Small HDL, nmol/L (n=1,653)	0.9 (-25.9, 3.3)	0.7066	1.6 (-0.6, 3.7)	0.3562	0.3 (-2.4, 2.9)	0.9115	0.6083	2.1 (-0.2, 4.3)	0.1884	**2.8 (0.7, 4.8)**	**0.0294**	-0.9 (-3.4, 1.7)	0.6875	0.2375
Total size of all HDL, nm (n=1,653)	-0.1 (-0.7, 0.5)	0.9015	0.03 (-0.5, 0.5)	0.9452	0.3 (-0.3, 1.0)	0.4975	**0.0185**	-0.3 (-0.9, 0.2)	0.4345	-0.2 (-0.7, 0.3)	0.5768	0.2 (-0.4, 0.8)	0.7315	**0.0727**

[a]Individuls were divided into 3 subgroups based on BMI scores: normal weight: 18.5 ≤BMI≤24.9, overweight: 25≤BMI≤29.9, obese: 30≤BMI≤50.
[b]Values presented are percent differences per 1 standard deviation increment in dietary index score obtained from multivariable-adjusted linear regression analyses and the bolded numbers represent statistically significant findings (i.e., FDR p value <0.10). Values are the beta coefficients.
[c]Linear regression models were adjusted for total energy intake, age, BMI-continuous, total recreational physical activity, pack-years of smoking, number of supplements used, fasting status at blood draw, race/ethnic groups, educational levels, regular use of NSAID, statins, unopposed estrogen and/or estrogen plus progesterone hormones, hormone therapy (HT) study arms.
[d]Abbreviations: HOMA-IR, Homeostatic model assessment of insulin resistance; HOMA β, Homeostatic model assessment of β-cell function; IGF-1, Insulin-like growth factor-1; IGF-BP1/3/4, Insulin-like growth factor-binding protein 1/3/4; TNF, tumor necrosis factor; VEGF, Vascular endothelial growth factor; VCAM-1, vascular cell adhesion protein 1; ICAM-1, intercellular adhesion molecule 1; GCSF, Granulocyte colony-stimulating factor

Supplemental Table 6. Associations of EDIH and EDIP with biomarkers in wasit-to-hip ratio subgroups

	**Empirical Dietary Index for Hyperinsulinemia (EDIH) score**					**Empirical Dietary Inflammatory Index (EDIP) score**				
	High waist-to-hip ratio (WHR[d] > 0.85)[a]		Low waist-to-hip ratio (WHR[d] ≤ 0.85)		FDR P-interaction	High waist-to-hip ratio (WHR > 0.85)		Low waist-to-hip ratio (WHR ≤ 0.85)		FDR P-interaction
Biomarkers	Percentage Difference[b,c]	FDR p value	Percentage Difference	FDR p value		Percentage Difference	FDR p value	Percentage Difference	FDR p value	
**Insulin and IGF pathway biomarkers**										
Glucose, mg/dL (n=21,669)	**1.4 (1.0, 1.9)**	**2.50E-08**	**1.1 (0.8, 1.3)**	**4.08E-18**	**1.82E-08**	**0.9 (0.4, 1.3)**	**0.0015**	**0.6 (0.4, 0.9)**	**7.58E-07**	**0.0001**
Insulin, ng/mL (n=23,756)	**5.1 (3.6, 6.5)**	**7.93E-11**	**5.5 (4.5, 6.5)**	**6.45E-25**	0.3532	**4.3 (2.9, 5.8)**	**5.98E-08**	**3.8 (2.8, 4.8)**	**4.06E-12**	0.1085
C-peptide, ng/mL (n=943)	0.4 (-5.9, 6.7)	0.9133	4.0 (0.2, 7.8)	0.1045	0.4869	1.4 (-5.7, 8.5)	0.7832	1.5 (-2.1, 5.1)	0.5264	0.6934
HOMA-IR[d] (n=19,865)	**7.3 (5.7, 8.9)**	**4.08E-18**	**6.6 (5.5, 7.6)**	**8.00E-29**	**0.0076**	**5.3 (3.8, 6.9)**	**6.00E-10**	**4.7 (3.6, 5.7)**	**3.03E-16**	**0.0007**
HOMA-B[d] (n=19865)	**2.0 (0.5, 3.5)**	**0.0302**	**2.5 (1.6, 3.5)**	**3.51E-06**	0.7345	**2.2 (0.8, 3.8)**	**0.01044**	**2.2 (1.2, 3.2)**	**0.0001**	0.6458
IGF[d]-1, pg/mL (n=3,126)	-0.7 (-5.1, 3.8)	0.8617	-0.7 (-4.2, 2.8)	0.8228	0.9532	0.6 (-3.8, 5.0)	0.8502	-0.4 (-3.3, 3.3)	0.9925	0.9874
IGFBP[d]1, pg/L (n= 993)	**-15.8 (-26.0, -5.5)**	**0.0126**	-3.7 (-9.2, 1.8)	0.3552	0.8126	**-14.9 (-27.7, -2.1)**	**0.0663**	0.4 (-5.0, 5.8)	0.9225	**0.0240**
IGFBP3, ng/mL (n=2,349)	1.1 (-2.7, 4.9)	0.7770	-1.1 (-3.2, 1.0)	0.5168	0.8311	-0.8 (-4.5, 2.8)	0.7487	1.4 (-0.6, 3.5)	0.2781	0.6432
IGFBP4, ng/mL (n=354)	-1.0 (-8.8, 6.8)	0.8617	3.1 (-2.9, 9.1)	0.5176	0.6083	-2.0 (-11.5, 7.5)	0.7574	5.6 (-0.4, 11.7)	0.1729	0.6083
free IGF-1, pg/mL (n=2,203)	4.8 (-2.7, 12.3)	0.3922	-0.8 (-5.6, 4.0)	0.8617	0.6458	3.1 (-4.3, 10.6)	0.5264	-4.1 (-8.6, 0.4)	0.1788	0.8749
**Inflammation and epithelial dysfunction biomarkers**										
C-reactive protein, mg/L (n=26,482)	**4.6 (2.7, 6.5)**	**1.49E-05**	**8.2 (6.8, 9.6)**	**8.00E-29**	**0.2060**	**4.0 (2.1, 5.9)**	**0.0002**	**6.8 (5.5, 8.2)**	**1.17E-20**	0.2894
Serum Amyloid A, mg/L (n=1,181)	4.6 (-4.7, 13.8)	0.5486	2.3 (-3.7, 8.2)	0.7015	0.9755	4.3 (-4.5, 13.2)	0.4916	2.5 (-2.9, 7.9)	0.5104	0.9964
Interleukin-6, pg/mL (n=12,408)	**6.1 (3.2, 9.1)**	**0.0003**	**5.3 (3.1, 7.4)**	**1.01E-05**	0.9176	**5.4 (2.5, 8.3)**	**0.0015**	**3.4 (.3, 5.4)**	**0.0063**	0.6083
Interleukin 10, pg/L (n=2,466)	**-9.5 (-17.2, -1.8)**	**0.0479**	-1.1 (-5.4, 3.2)	0.7997	0.8329	**-7.9 (-15.0, -0.8)**	**0.0779**	-1.8 (-5.9, 2.3)	0.5264	0.8353
TNF alpha, mg/mL (n=5,302)	**10.1 (2.6, 17.6)**	**0.0302**	**5.9 (1.4, 10.5)**	**0.0355**	0.8377	**8.0 (0.9, 15.1)**	**0.0765**	2.3 (-2.1, 6.7)	0.4499	0.7913
TNF alpha receptor 1, pg/mL (n=3,908)	**2.2 (0.2, 4.2)**	**0.0970**	-0.03 (-1.2, 1.1)	0.9549	**0.1085**	**2.9 (1.0, 4.8)**	**0.0106**	-0.001 (-1.1, 1.1)	0.9983	0.1085
TNF alpha receptor 2, pg/mL (n=7,746)	1.5 (-0.05, 3.1)	0.1338	1.0 (0.01, 2.1)	0.1145	0.8543	**2.1 (0.5, 3.7)**	**0.0294**	**1.8 (0.8, 2.8)**	**0.0017**	0.6083
Adiponectin, ng/mL (n=7552)	-0.4 (-3.7, 3.0)	0.8755	**-2.3 (-4.3, -0.2)**	**0.0793**	0.9532	-1.0 (-4.3, 2.3)	0.6591	**-2.9 (-4.8, -0.9)**	**0.0141**	0.7701
Leptin, ng/mL (n=8,045)	2.1 (-0.5, 4.7)	0.2340	**6.5 (4.6, 8.5)**	**4.14E-10**	**0.0016**	1.2 (-1.2, 3.6)	0.4916	**3.6 (1.7, 5.4)**	**0.0008**	0.1644

VEGF[d], pg.ml (n=873)	-1.1 (-19.1, 16.8)	0.9133	-1.6 (-7.1, 3.9)	0.7770	0.8543	-5.0 (-21.3, 11.4)	0.6591	-1.3 (-6.6, 3.9)	0.7214	0.9935
E-selectin, ng/ml (n=3,817)	**5.7 (2.8, 8.6)**	**7.51E-04**	0.5 (-1.5, 2.6)	0.7997	0.3373	**4.8 (1.9, 7.7)**	**0.0048**	1.3 (-0.8, 3.3)	0.3525	0.1617
VCAM-1[d], ng.ml (n=4,050)	2.0 (-0.2, 4.2)	0.1703	-0.4 (-1.8, 0.9)	0.7742	0.6458	**3.1 (0.9, 5.2)**	**0.0160**	0.2 (-1.1, 1.5)	0.8493	0.1735
ICAM-1[d], ng/ml (n=1,075)	**9.6 (3.1, 16.2)**	**0.0194**	3.1 (0.1, 6.2)	0.1072	0.7437	**8.9 (2.6, 15.1)**	**0.0190**	2.0 (-0.8, 4.9)	0.2781	0.4125
GCSF[d], pg/ml (n=2,160)	-0.9 (-8.2, 6.3)	0.8617	3.9 (-0.8, 8.5)	0.2279	0.9532	2.2 (-4.6, 9.0)	0.6442	-0.2 (-4.6, 4.3)	0.9642	0.8691
**Lipid and lipid particle biomarkers**										
Total cholesterol, mg/dL (n=21,378)	-0.09 (-0.6, 0.4)	0.8329	**0.4 (0.06, 0.7)**	**0.0593**	0.1911	-0.3 (-0.7, 0.2)	0.3811	0.2 (-0.1, 0.5)	0.2719	**0.0579**
Triglycerides, mg/dL (n=18,833)	0.3 (-1.0, 1.5)	0.8228	**1.9 (1.1, 2.7)**	**2.45E-05**	0.6015	**1.3 (0.1, 2.5)**	**0.1014**	**3.1 (2.3, 3.9)**	**6.34E-13**	0.6458
High density Lipoprotein (HDL), mg/dL (n=20,508)	-0.1 (-0.8, 0.5)	0.8228	**-0.5 (-1.0, -0.1)**	**0.0415**	0.6293	-0.5 (-1.2, 0.1)	0.1914	**-1.2 (-1.7, -0.8)**	**7.24E-08**	0.9532
Low density Lipoprotein (LDL), mg/dL (n=16,525)	-0.2 (-1.0, 0.5)	0.7765	**0.7 (0.2, 1.2)**	**0.0240**	**0.0211**	-0.7 (-1.4, 0.1)	0.1788	0.5 (-0.05, 1.0)	0.1788	**0.0005**
Triglycerides/High density Lipoprotein (n=17,761)	0.4 (-1.2, 2.1)	0.7997	**2.8 (1.7, 3.9)**	**8.56E-06**	0.6468	1.4 (-0.3, 3.1)	0.2088	**4.4 (3.2, 5.5)**	**5.17E-13**	0.5176
Triglycerides/Total cholesterol (n=18,631)	0.4 (-0.7, 1.6)	0.7015	**1.5 (0.7, 2.2)**	**7.51E-04**	**0.1393**	**1.8 (0.6, 2.9)**	**0.0118**	**2.8 (2.0, 3.5)**	**1.21E-11**	0.9532
Large LDL, nmol/L (n=1,653)	-1.6 (-6.1, 2.9)	0.7446	0.4 (-2.4, 3.1)	0.8617	0.3923	-3.4 (-7.8, 1.0)	0.2430	-0.9 (-3.5, 1.7)	0.6337	0.5666
Medium LDL, nmol/L (n=1,356)	-1.6 (-7.0, 3.9)	0.7823	2.0 (-2.3, 6.3)	0.5733	0.9937	2.4 (-2.9, 7.7)	0.5134	3.4 (-0.7, 7.5)	0.2226	0.9532
Small LDL, nmol/L (n=1,653)	-3.9 (-8.9, 1.1)	0.2611	2.9 (-1.3, 7.1)	0.3364	0.6717	-0.4 (-5.3, 4.5)	0.9090	3.1 (-0.9, 7.0)	0.2458	0.7030
Very small LDL, nmol/L (n=1,356)	-1.7 (-7.1, 3.7)	0.7765	3.0 (-1.2, 7.2)	0.3266	0.9532	2.4 (-2.8, 7.5)	0.2458	3.2 (-0.8, 7.2)	0.2430	0.9965
Total size of all LDL, nm (n=1,652)	-0.07 (-0.4, 0.3)	0.8228	-0.1 (-0.3, 0.1)	0.4243	0.6934	-0.2 (-0.6, 0.1)	0.5134	-0.1 (-0.3, 0.1)	0.3523	0.6934
Intermediate density lipoprotein, nmol/L (n=1,653)	2.0 (-6.3, 10.3)	0.7997	0.9 (-5.5, 7.3)	0.8617	0.7913	2.2 (-5.9, 10.3)	0.7011	2.6 (-3.5, 8.7)	0.5264	0.8438
Large HDL, nmol/L (n=1,653)	3.8 (-1.6, 9.2)	0.3364	0.3 (-3.2, 3.7)	0.9133	0.7437	3.3 (-2.0, 8.7)	0.3525	-1.2 (-4.5, 2.1)	0.5918	0.6087
Medium HDL, nmol/L (n=1,241)	**16.0 (0.6, 31.4)**	**0.1062**	1.1 (-9.9, 12.1)	0.8897	0.7437	10.4 (-5.2, 26.1)	0.3249	1.6 (-8.7, 12.0)	0.8279	0.6194
Small HDL, nmol/L (n=1,653)	-1.3 (-3.5, 0.8)	0.4097	**2.4 (0.7, 4.1)**	**0.0227**	0.6738	-1.1 (-3.2, 1.0)	0.4454	**2.7 (1.1, 4.3)**	**0.0045**	0.2487
Total size of all HDL, nm (n=1,653)	**0.6 (0.1, 1.1)**	**0.0460**	-0.2 (-0.6, 0.2)	0.5492	0.3220	0.4 (-0.05, 0.9)	0.1788	-0.4 (-0.7, 0.01)	0.1422	0.2072

[a]Individuls were grouped based on Waist/Hip Ratio (WHR) scores. WHR=waist circumference (cm)/hip circumference (cm). Two subgroups: WHR>0.85 and WHR≤0.85

[a]Values presented are percent differences per 1 standard deviation increment in dietary index score obtained from multivariable-adjusted linear regression analyses and the bolded numbers represent statistically significant findings (i.e., FDR p value <0.10). Values are the beta coefficients.

[c]Models were adjusted for total energy intake, age, BMI-continuous, total recreational physical activity, pack-years of smoking; number of supplements used, fasting status at blood draw, race/ethnic groups, educational levels, regular use of NSAID, statins, unopposed estrogen and/or estrogen plus progesterone hormones, hormone therapy (HT) study arms.

[d]Abbreviations: WHR, Waist/Hip Ratio; HOMA-IR, Homeostatic model assessment of insulin resistance; HOMA β, Homeostatic model assessment of β-cell function; IGF-1, Insulin-like growth factor-1; IGF-BP1/3/4, Insulin-like growth factor-binding protein 1/3/4; TNF, tumor necrosis factor; VEGF, Vascular endothelial growth factor; VCAM-1, vascular cell adhesion protein 1; ICAM-1, intercellular adhesion molecule 1; GCSF, Granulocyte colony-stimulating factor

Supplemental Table 7. Associations of EDIH and EDIP with biomarkers in race and ethnicity subgroups

	Empirical Dietary Index for Hyperinsulinemia (EDIH) score							Empirical Dietary Inflammatory Index (EDIP) score						
	Non-Hispanic White[a]		African American[a]		Hispanic[a]			Non-Hispanic White		African American		Hispanic		
Biomarkers	Percentage Difference[b,c]	FDR p-value	Percentage Difference	FDR p-value	Percentage Difference	FDR p-value	FDR P-interaction	Percentage Difference	FDR p-value	Percentage Difference	FDR p-value	Percentage Difference	FDR p-value	FDR P-interaction
**Insulin and IGF pathway biomarkers**														
Glucose, mg/dL (n=21,669)	**1.2 (0.9, 1.5)**	**7.08E-17**	**1.5 (1.0, 2.0)**	**3.13E-08**	**1.7 (1.0, 2.3)**	**1.31E-05**	0.9261	**0.7 (0.4, 1.0)**	**1.52E-06**	**1.3 (0.7, 1.8)**	**6.79E-05**	**1.1 (0.4, 1.8)**	**0.0079**	0.9755
Insulin, ng/mL (n=23,756)	**6.1 (5.0, 7.2)**	**1.95E-26**	**6.6 (5.0, 8.3)**	**3.06E-13**	**6.0 (3.3, 8.7)**	**0.0001**	0.8353	**4.7 (3.6, 5.7)**	**2.76E-16**	**5.8 (3.9, 7.6)**	**1.50E-08**	**4.9 (2.2, 7.6)**	**0.0027**	0.6934
C-peptide, ng/mL (n=943)	**5.3 (1.8, 8.7)**	**0.0195**	1.4 (-14.1, 16.9)	0.9308	-3.7 (-38.4, 30.9)	0.8553	0.9532	1.7 (-1.7, 5.1)	0.5146	15.6 (-2.4, 33.7)	0.2054	-8.7 (-36.6, 19.2)	0.5985	0.8543
HOMA-IR[d] (n=19,865)	**7.6 (6.4, 8.7)**	**1.53E-28**	**8.2 (6.2, 10.1)**	**2.77E-15**	**8.0 (5.1, 11.0)**	**1.18E-06**	0.8126	**5.5 (4.4, 6.6)**	**1.03E-19**	**7.1 (4.9, 9.2)**	**1.23E-09**	**7.1 (4.2, 10.0)**	**2.55E-05**	0.4819
HOMA-B[d] (n=19865)	**3.1 (2.0, 4.1)**	**1.38E-07**	**2.4 (0.7, 4.2)**	**0.0352**	1.6 (-1.1, 4.2)	0.4818	0.5027	**2.9 (1.9, 3.9)**	**4.76E-07**	**2.1 (0.2, 4.0)**	**0.1079**	**3.2 (0.6, 5.9)**	**0.0738**	0.5313
IGF[d]-1, pg/mL (n=3,126)	-0.6 (-3.6, 2.3)	0.7943	15.2 (-2.0, 32.3)	0.2402	-7.8 (-24.3, 8.7)	0.5642	0.9524	-0.4 (-3.3, 2.4)	0.8630	19.7 (4.2, 35.3)	**0.0619**	-1.9 (-18.9, 15.1)	0.8935	0.9532
IGFBP[d]1, pg/L (n= 993)	**-10.4 (-15.4, -5.4)**	**0.0005**	-0.4 (-43.4, 42.5)	0.9958	-31.8 (-74.7, 11.2)	0.3226	0.9261	-5.5 (-10.7, -0.2)	0.1202	-3.2 (-53.7, 47.2)	0.9457	-12.4 (-65.9, 41.2)	0.7462	0.9621
IGFBP3, ng/mL (n=2,349)	-0.3 (-2.3, 1.6)	0.8469	-2.0 (-10.5, 6.4)	0.7561	3.1 (-3.7, 9.9)	0.5775	0.6934	1.3 (-0.6, 3.2)	0.3446	-2.6 (-10.6, 5.5)	0.7145	-3.0 (-12.0, 6.0)	0.6956	0.1713
IGFBP4, ng/mL (n=354)	2.7 (-2.3, 7.6)	0.4994	1.6 (-48.6, 51.8)	0.9717	-	-	0.7651	4.6 (-0.7, 9.9)	0.2054	12.2 (-33.2, 57.6)	0.7236	-	-	0.6015
free IGF-1, pg/mL (n=2,203)	0.1 (-4.3, 4.5)	0.9900	1.3 (-13.8, 16.4)	0.9313	-0.8 (-18.0, 16.3)	0.9580	0.9874	-2.6 (-6.8, 1.5)	0.3619	-3.4 (-18.2, 11.4)	0.7712	0.9 (-20.4, 22.2)	0.9647	0.9937
**Inflammation and epithelial dysfunction biomarkers**														
C-reactive protein, mg/L (n=26,482)	**8.0 (6.6, 9.3)**	**2.30E-28**	**8.4 (5.6, 11.2)**	**7.42E-08**	**5.1 (1.6, 8.7)**	**0.0309**	0.7651	**6.7 (5.4, 8.1)**	**1.11E-21**	**8.3 (5.2, 11.3)**	**1.52E-06**	3.5 (0.02, 7.1)	0.1354	0.6934
Serum Amyloid A, mg/L (n=1,181)	1.4 (-4.1, 7.0)	0.7531	12.1 (-5.9, 30.2)	0.4230	-18.8 (-60.2, 22.5)	0.5325	0.6293	3.3 (-1.7, 8.3)	0.3558	2.0 (-16.9, 20.9)	0.8992	-31.2 (-70.3, 7.8)	0.2210	0.9964
Interleukin-6, pg/mL (n=12,408)	**6.1 (1.0, 8.1)**	**1.40E-07**	**5.5 (1.2, 9.7)**	**0.0601**	3.8 (-2.8, 10.4)	0.4807	0.9937	**3.6 (1.6, 5.6)**	**0.0026**	**9.5 (4.9, 14.1)**	**0.0005**	2.3 (-4.1, 8.7)	0.6799	0.9160
Interleukin 10, pg/L (n=2,466)	-4.7 (-9.6, 0.1)	0.1794	-2.2 (-10.4, 6.1)	0.7531	-4.6 (-13.2, 4.0)	0.5024	0.7437	-4.7 (-9.2, -0.2)	0.1193	-3.2 (-12.0, 5.6)	0.6754	-5.2 (-13.6, 3.1)	0.3705	0.9532
TNF[d] alpha, mg/mL (n=5,302)	**6.0 (1.5, 10.5)**	**0.0504**	**12.7 (1.9, 23.4)**	**0.0886**	5.1 (-5.8, 16.0)	0.5721	0.9524	0.7 (-3.5, 4.9)	0.8504	**22.6 (11.3, 33.9)**	**0.0007**	3.3 (-7.8, 14.4)	0.7227	0.1268

TNF alpha receptor 1, pg/mL (n=3,908)	1.1 (-0.3, 2.4)	0.3003	1.0 (-1.4, 3.3)	0.6239	-2.0 (-4.8, 0.9)	0.4074	0.7913	1.0 (-0.2, 2.2)	0.2298	1.1 (-1.3, 3.5)	0.5730	-0.9 (-3.6, 1.2)	0.7227	0.8838
TNF alpha receptor 2, pg/mL (n=7,746)	1.2 (0.05, 2.3)	0.1386	1.6 (-0.02, 3.2)	0.1747	0.9 (-2.2, 4.0)	0.7332	0.8691	**2.3 (1.2, 3.4)**	**0.0003**	0.2 (-1.6, 1.9)	0.9110	3.2 (0.2, 6.2)	0.1160	0.7437
Adiponectin, ng/mL (n=7552)	-1.6 (-3.6, 0.5)	0.3358	**-5.6 (-10.1, -1.0)**	**0.0796**	-4.2 (-11.2, 2.7)	0.4684	0.9261	-2.1 (-4.1, -0.2)	0.1160	**-6.9 (-11.7, -2.1)**	**0.0274**	-4.3 (-11.3, 2.7)	0.3824	0.7951
Leptin, ng/mL (n=8,045)	**4.5 (2.6, 6.5)**	**4.81E-05**	**5.3 (1.8, 8.7)**	**0.0212**	4.7 (-0.5, 9.9)	0.2359	0.3923	**2.5 (0.7, 4.3)**	**0.0368**	3.9 (0.1, 7.6)	0.1203	1.7 (-3.4, 6.8)	0.6956	0.4036
VEGF[d], pg.ml (n=873)	1.8 (-4.6, 8.3)	0.7343	-3.8 (-10.5, 2.9)	0.4855	-	-	**0.0091**	2.3 (-3.9, 8.6)	0.6661	-3.3 (-9.6, 2.9)	0.4885	-	-	**0.0091**
E-selectin, ng/ml (n=3,817)	**2.4 (0.4, 4.5)**	**0.0870**	3.9 (-0.2, 7.9)	0.1926	**7.7 (1.1, 14.3)**	**0.0886**	0.8691	**2.5 (0.6, 4.5)**	**0.0554**	4.5 (-0.04, 9.0)	0.1427	6.0 (-0.2, 12.3)	0.1453	0.8904
VCAM-1[d], ng.ml (n=4,050)	0.8 (-0.6, 2.2)	0.4799	-0.8 (3.4, 1.9)	0.7343	0.01 (-3.9, 3.9)	0.9958	0.6083	**1.6 (0.3, 2.9)**	**0.0619**	0.5 (-2.5, 3.5)	0.8515	1.3 (-2.2, 4.8)	0.6661	0.4345
ICAM-1[d], ng/ml (n=1,075)	**3.6 (0.4, 6.9)**	**0.1010**	13.2 (-8.9, 35.4)	0.4684	**7.7 (1.5, 13.8)**	**0.0764**	0.7109	1.9 (-1.1, 4.9)	0.3619	6.8 (-17.4, 30.9)	0.7227	**6.9 (1.5, 12.4)**	**0.0606**	0.7437
GCSF[d], pg/ml (n=2,160)	2.5 (-2.4, 7.4)	0.5300	-0.9 (-7.3, 5.4)	0.8733	7.2 (-9.6, 24.1)	0.6113	0.8543	1.2 (-3.3, 5.6)	0.7462	0.4 (-6.5, 7.3)	0.9457	-2.5 (-19.4, 14.4)	0.8717	0.8950
**Lipid and lipid particle biomarkers**														
Total cholesterol, mg/dL (n=21,378)	0.3 (0.01, 0.6)	0.1386	0.6 (0.04, 1.2)	0.1254	-0.5 (-1.3, 0.2)	0.4094	0.3220	0.04 (-0.3, 0.3)	0.8747	0.6 (-0.04, 1.2)	0.1596	-0.4 (-1.2, 0.4)	0.4689	0.5622
Triglycerides, mg/dL (n=18,833)	**2.1 (1.2, 3.0)**	**4.22E-05**	**2.1 (0.8, 3.5)**	**0.0182**	1.3 (-0.8, 3.4)	0.4684	0.5027	**3.4 (2.6, 4.3)**	**1.49E-13**	**3.2 (1.7, 4.8)**	**0.0003**	2.2 (0.1, 4.3)	0.1175	0.5121
High density Lipoprotein (HDL), mg/dL (n=20,508)	**-0.6 (-1.1, -0.1)**	**0.0563**	-0.4 (-1.2, 0.3)	0.4925	**-1.2 (-2.3, -0.2)**	**0.0886**	0.6083	**-1.5 (-2.0, -1.1)**	**1.03E-09**	-0.9 (-1.7, -0.1)	0.1118	**-1.2 (-2.2, -0.2)**	**0.0888**	0.4125
Low density Lipoprotein (LDL), mg/dL (n=16,525)	**0.6 (0.1, 1.2)**	**0.0886**	0.7 (-0.1, 1.6)	0.2737	-0.6 (-1.9, 0.6)	0.5295	0.4263	0.07 (-0.5, 0.6)	0.8747	0.7 (-0.2, 1.7)	0.2600	-0.8 (-2.0, 0.4)	0.3601	0.1617
Triglycerides/High density Lipoprotein (n=17,761)	**3.1 (1.8, 4.3)**	**1.44E-05**	**2.7 (0.8, 4.6)**	**0.0309**	2.5 (-0.3, 5.3)	0.2402	0.4869	**4.9 (3.6, 6.1)**	**1.43E-13**	**4.1 (2.1, 6.2)**	**0.0006**	3.3 (0.6, 6.1)	**0.0755**	0.4819
Triglycerides/Total cholesterol (n=18,631)	**1.7 (0.8, 2.5)**	**0.0006**	**1.6 (0.3, 3.0)**	**0.0796**	1.8 (-0.2, 3.7)	0.2308	0.5465	**3.3 (2.5, 4.1)**	**1.61E-14**	**2.6 (1.1, 4.0)**	**0.0036**	**2.7 (0.7, 4.6)**	**0.0373**	0.1332
Large LDL, nmol/L (n=1,653)	-0.8 (-3.4, 1.7)	0.6849	-4.3 (-11.7, 3.1)	0.4799	11.1 (-8.1, 30.3)	0.4799	0.8691	**-2.7 (-5.1, -0.4)**	**0.0888**	-5.5 (-13.9, 2.8)	0.3558	5.6 (-15.7, 26.9)	0.7318	0.8543
Medium LDL, nmol/L (n=1,356)	0.9 (-2.7, 4.5)	0.7531	**25.9 (4.9, 47.0)**	**0.0796**	7.0 (-28.6, 42.5)	0.7724	0.6133	**3.9 (0.6, 7.3)**	**0.0881**	**30.6 (10.9, 50.3)**	**0.0164**	-1.9 (-40.7, 36.9)	0.9497	0.5121
Small LDL, nmol/L (n=1,653)	0.9 (-2.5, 4.4)	0.7468	9.0 (-3.4, 21.3)	0.3663	-13.9 (-35.8, 8.0)	0.4438	0.8950	3.2 (-0.05, 6.5)	0.1444	14.8 (1.0, 28.7)	0.1160	**-28.7 (-49.8, -7.7)**	**0.0461**	0.5465
Very small LDL, nmol/L (n=1,356)	1.4 (-2.1, 4.8)	0.6318	**26.8 (6.7, 46.8)**	**0.0549**	10.1 (-23.5, 43.6)	0.6835	0.7345	3.6 (0.3, 6.9)	0.1118	**31.5 (12.8, 50.1)**	**0.0079**	0.3 (-37.0, 37.6)	0.9850	0.6050
Total size of all LDL, nm (n=1,652)	-0.2 (-0.4, 0.03)	0.2893	-0.6 (-1.2, -0.1)	**0.1058**	0.9 (-0.4, 2.3)	0.3654	0.6738	**-0.2 (-0.4, -0.06)**	**0.0437**	**-0.9 (-1.6, -0.3)**	**0.0259**	1.3 (-0.1, 2.7)	0.1596	0.6448
Intermediate density lipoprotein, nmol/L (n=1,653)	3.2 (-2.4, 8.7)	0.4825	-4.0 (-18.8, 10.8)	0.7468	3.0 (-36.5, 41.6)	0.9344	0.8691	3.5 (-1.7, 8.7)	0.3466	13.5 (-3.2, 30.1)	0.2340	-11.1 (-52.6, 30.3)	0.7314	0.7913

Large HDL, nmol/L (n=1,653)	1.0 (-2.1, 4.2)	0.6849	-7.7 (-17.9, 2.5)	0.3435	15.1 (-9.4, 39.7)	0.4674	0.9125	-1.5 (-4.4, 1.5)	0.5146	-9.8 (-21.4, 1.7)	0.2133	24.5 (-0.7, 49.7)	0.1453	0.9261
Medium HDL, nmol/L (n=1,241)	3.8 (-5.9, 13.5)	0.6265	12.8 (-18.7, 44.2)	0.6239	33.7 (-43.1, 110.5)	0.5756	0.9935	0.3 (-8.9, 9.5)	0.9707	29.0 (-5.5, 63.5)	0.2148	36.2 (-48.3, 120.8)	0.5805	0.8230
Small HDL, nmol/L (n=1,653)	1.2 (-0.3, 2.6)	0.2893	3.5 (-0.9, 7.9)	0.2997	1.5 (-11.7, 14.6)	0.9023	0.7899	**1.6 (0.2, 2.9)**	**0.0888**	3.2 (-1.8, 8.2)	0.3601	-5.9 (-19.9, 8.0)	0.5915	0.8088
Total size of all HDL, nm (n=1,653)	0.03 (-0.3, 0.4)	0.9262	-0.7 (-1.8, 0.4)	0.4684	1.1 (-1.7, 3.8)	0.6265	0.9468	-0.3 (-0.6, 0.02)	0.1591	-0.8 (-2.1, 0.4)	0.3446	2.8 (0.1, 5.5)	0.1295	0.8691

[a]Individuls were grouped into 3 subgroups based on race/ethnicity: White (not of Hispanic origin), Black or African-American and Hispanic/Latino. There were not enough participants for subgroup analyses in other racial/ethnic subgroups (Asians/Pacific Islander, Alaskan Natives, Other).

[b]Values presented are percent differences per 1 standard deviation increment in dietary index score obtained from multivariable-adjusted linear regression analyses and the bolded numbers represent statistically significant findings (i.e., FDR p value <0.10). Values are the beta coefficients.

[c]Models were adjusted for total energy intake, age, BMI-continuous, total recreational physical activity, pack-years of smoking, number of supplements used, fasting status at blood draw, educational levels, regular use of NSAID, statins, unopposed estrogen and/or estrogen plus progesterone hormones, hormone therapy (HT) study arms.

[d]Abbreviations: HOMA-IR, Homeostatic model assessment of insulin resistance; HOMA β, Homeostatic model assessment of β-cell function; IGF-1, Insulin-like growth factor-1; IGF-BP1/3/4, Insulin-like growth factor-binding protein 1/3/4; TNF, tumor necrosis factor; VEGF, Vascular endothelial growth factor; VCAM-1, vascular cell adhesion protein 1; ICAM-1, intercellular adhesion molecule 1; GCSF, Granulocyte colony-stimulating factor

Supplemental Table 8. Associations of EDIH and EDIP with biomarkers in categories of regular statin use

	**Empirical Dietary Index for Hyperinsulinemia (EDIH) score**					**Empirical Dietary Inflammatory Index (EDIP) score**				
	Statin non-users[a]		Statin regular users[a]		FDR P-interaction	Statin non-users		Statin regular users		FDR P-interaction
Biomarkers	Percentage Difference[b,c]	FDR p value	Percentage Difference	FDR p value		Percentage Difference	FDR p value	Percentage Difference	FDR p value	
**Insulin and IGF pathway biomarkers**										
Glucose, mg/dL (n=21,669)	**1.3 (1.1, 1.5)**	**5.90E-29**	0.9 (-0.8, 2.7)	0.6380	0.8543	**0.8 (0.6, 1.0)**	**1.86E-12**	0.9 (-0.6, 2.5)	0.4075	0.9261
Insulin, ng/mL (n=23,756)	**7.5 (6.6, 8.3)**	**2.60E-29**	5.8 (-0.7, 12.2)	0.2728	0.8126	**6.8 (6.0, 7.7)**	**4.00E-29**	**6.4 (0.7, 12.2)**	**0.1002**	0.8416
C-peptide, ng/mL (n=943)	**5.1 (1.9, 8.4)**	**0.0129**	-19.4 (-46.6, 7.8)	0.3826	0.6488	2.5 (-0.7, 5.7)	0.2982	-2.2 (-60.7, 17.6)	0.4075	0.5176
HOMA-IR[d] (n=19,865)	**8.4 (7.5, 9.3)**	**2.60E-29**	**9.1 (2.6, 15.6)**	**0.0309**	0.9627	**6.8 (5.9, 7.8)**	**4.00E-29**	**8.9 (3.2, 14.7)**	**0.0139**	0.9281
HOMA-B[d] (n=19865)	**3.6 (2.8, 4.4)**	**5.18E-16**	3.2 (-3.2, 9.5)	0.6464	0.9524	**3.8 (3.0, 4.6)**	**4.36E-18**	4.7 (-0.8, 10.2)	0.2339	0.9755
IGF[d]-1, pg/mL (n=3,126)	-0.5 (-3.4, 2.5)	0.8470	-18.1 (-49.7, 13.6)	0.5652	0.6934	-2.0 (-4.8, 0.8)	0.3354	2.3 (-36.7, 41.1)	0.9438	0.8691
IGFBP[d]1, pg/L (n= 993)	**-9.8 (-14.8, 4.8)**	**0.0012**	-3.5 (-233.8, 226.8)	0.9663	0.7913	**-7.3 (-12.5, -2.1)**	**0.0337**	48.9 (-134.8, 232.7)	0.5320	0.4718
IGFBP3, ng/mL (n=2,349)	-0.7 (-2.5, 1.1)	0.6923	10.5 (-11.6, 32.7)	0.6464	0.6738	0.7 (-1.1, 2.4)	0.6057	12.9 (-2.0, 27.8)	0.2339	0.8230
IGFBP4, ng/mL (n=354)	2.0 (-2.4, 6.4)	0.6677	-	-	0.4036	2.1 (-2.6, 6.8)	0.5352	-	-	0.3161
free IGF-1, pg/mL (n=2,203)	1.2 (-2.8, 5.3)	0.7601	1.3 (-35.7, 38.2)	0.9663	0.9176	-1.8 (-5.6, 2.1)	0.5320	-1.0 (-31.2, 29.1)	0.9553	0.9874
**Inflammation and epithelial dysfunction biomarkers**										
C-reactive protein, mg/L (n=26,482)	**7.7 (6.6, 8.9)**	**2.60E-29**	7.2 (-1.6, 16.1)	0.3317	0.8543	**6.2 (5.0, 7.3)**	**1.50E-25**	6.2 (-1.6, 14.0)	0.2858	0.7326
Serum Amyloid A, mg/L (n=1,181)	3.7 (-1.3, 8.6)	0.3949	-50.5 (-1007.6, -2.7)	0.1846	0.9176	3.5 (-1.0, 8.0)	0.2882	-54.6 (-98.1, -11.1)	0.0908	0.6458
Interleukin-6, pg/mL (n=12,408)	**6.3 (4.5, 8.1)**	**3.01E-11**	-2.0 (-14.6, 10.7)	0.8470	0.6458	**4.8 (3.1, 6.5)**	**2.14E-07**	-4.3 (-15.7, 7.1)	0.6057	0.5574
Interleukin 10, pg/L (n=2,466)	-4.3 (-8.0, -0.5)	0.1126	17.9 (-26.3, 62.0)	0.6890	0.8353	-4.1 (-7.5, -0.6)	0.0908	26.7 (-19.1, 72.5)	0.4075	0.9176
TNF[d] alpha, mg/mL (n=5,302)	**6.0 (2.1, 9.9)**	**0.0132**	22.2 (-8.2, 53.5)	0.3949	0.9532	3.2 (-0.5, 6.9)	0.2339	9.4 (-18.1, 37.0)	0.6228	0.9160
TNF alpha receptor 1, pg/mL (n=3,908)	0.2 (-0.8, 1.3)	0.8470	-3.9 (-13.6, 5.7)	0.6890	0.9935	-0.03 (-1.0, 0.9)	0.9574	-9.5 (-18.4, -0.6)	0.1260	0.8577
TNF alpha receptor 2, pg/mL (n=7,746)	0.4 (-0.5, 1.3)	0.6677	-1.8 (-9.6, 5.9)	0.8465	0.9532	0.06 (-0.8, 0.9)	0.9426	-2.8 (-10.4, 4.9)	0.6146	0.6083
Adiponectin, ng/mL (n=7552)	**-3.7 (-5.5, -1.8)**	**0.0009**	6.7 (-11.1, 24.5)	0.6923	0.8438	**-6.3 (-8.1, -4.6)**	**3.75E-11**	-9.3 (-22.3, 3.6)	0.3345	0.9532
Leptin, ng/mL (n=8,045)	**6.6 (5.0, 8.2)**	**2.63E-14**	5.5 (-6.6, 17.7)	0.6677	0.9991	**4.5 (3.0, 6.0)**	**8.52E-08**	2.8 (-7.0, 12.8)	0.6770	0.9624
VEGF[d], pg.ml (n=873)	-0.8 (-6.3, 4.7)	0.8619	-11.6 (-87.9, 64.7)	0.8470	0.6194	0.5 (-4.6, 5.6)	0.9093	-39.5 (-132.2, 53.2)	0.4777	0.6083
E-selectin, ng/ml (n=3,817)	**2.8 (1.1, 4.6)**	**0.0071**	4.9 (-12.5, 22.3)	0.7864	0.9281	**3.8 (2.1, 5.5)**	**5.72E-05**	7.7 (-4.6, 20.0)	0.3875	0.8543

VCAM-1[d], ng.ml (n=4,050)	0.2 (-1.1, 1.3)	0.8470	4.0 (-5.6, 13.5)	0.6890	0.8577	0.7 (-0.4, 1.7)	0.4075	5.2 (-2.9, 13.4)	0.3875	0.7444
ICAM-1[d], ng/ml (n=1,075)	**5.1 (2.3, 7.8)**	**0.0029**	-19.1 (-53.2, 14.9)	0.5438	0.7913	**3.1 (0.6, 5.7)**	**0.0792**	-20.3 (-78.4, 37.8)	0.6057	0.7205
GCSF[d], pg/ml (n=2,160)	2.4 (-1.4, 6.3)	0.5390	15.3 (-93.7, 124.2)	0.8470	0.9532	0.5 (-3.1, 4.1)	0.8894	47.3 (-53.6, 148.3)	0.4827	0.9964
**Lipid and lipid particle biomarkers**										
Total cholesterol, mg/dL (n=21,378)	0.3 (0.004, 0.5)	0.1846	-0.9 (-2.6, 0.9)	0.6464	0.6194	0.04 (-0.2, 0.3)	0.8668	-1.0 (-2.7, 0.6)	0.3875	0.4988
Triglycerides, mg/dL (n=18,833)	0.7 (0.003, 1.5)	0.1846	0.9 (-3.9, 5.7)	0.8470	0.6738	**1.5 (0.7, 2.2)**	**0.0005**	3.1 (-1.3, 7.4)	0.3354	0.9876
High density Lipoprotein (HDL), mg/dL (n=20,508)	**-0.5 (-0.9, -0.1)**	**0.0384**	0.1 (-2.4, 2.6)	0.9663	0.8126	**-1.1 (-1.4, -0.7)**	**3.37E-08**	-1.0 (-3.4, 1.3)	0.5564	0.9937
Low density Lipoprotein (LDL), mg/dL (n=16,525)	**0.7 (0.3, 1.2)**	**0.0070**	-1.7 (-4.5, 1.1)	0.5438	0.5465	0.4 (-0.06, 0.8)	0.2339	-1.2 (-3.8, 1.4)	0.5320	0.6210
Triglycerides/High density Lipoprotein (n=17,761)	**1.5 (0.5, 2.5)**	**0.0187**	0.5 (-5.9, 6.9)	0.9495	0.6107	**2.5 (1.6, 3.5)**	**3.29E-06**	3.9 (-1.9, 9.8)	0.3733	0.9930
Triglycerides/Total cholesterol (n=18,631)	0.4 (-0.3, 1.1)	0.5390	1.8 (-2.9, 6.5)	0.6923	0.8353	**1.4 (0.7, 2.0)**	**0.0005**	3.7 (-0.6, 8.0)	0.2339	0.8691
Large LDL, nmol/L (n=1,653)	-0.9 (-3.2, 1.5)	0.6923	2.2 (-34.1, 38.5)	0.9597	0.7899	-2.1 (-4.3, 0.1)	0.1913	-7.5 (-31.5, 16.5)	0.6277	0.7899
Medium LDL, nmol/L (n=1,356)	1.7 (-1.7, 5.1)	0.6464	-6.8 (-45.3, 31.7)	0.8470	0.9669	3.7 (0.5, 7.0)	0.0908	2.5 (-24.3, 29.3)	0.9093	0.8691
Small LDL, nmol/L (n=1,653)	1.0 (-2.2, 4.3)	0.7524	-7.2 (-39.3, 24.9)	0.8465	0.8950	1.7 (-1.4, 4.8)	0.4476	2.7 (-14.7, 28.0)	0.6277	0.6083
Very small LDL, nmol/L (n=1,356)	2.3 (-1.0, 5.6)	0.4433	-11.2 (-45.4, 23.0)	0.6939	0.9782	**3.6 (0.5, 6.8)**	**0.0908**	-0.8 (-25.2, 23.5)	0.9553	0.9261
Total size of all LDL, nm (n=1,652)	-0.2 (-0.4, 0.004)	0.1983	0.8 (-1.3, 2.8)	0.6923	0.8353	**-0.2 (-0.4, -0.04)**	**0.0656**	-0.1 (-1.5, 1.3)	0.9426	0.8240
Intermediate density lipoprotein, nmol/L (n=1,653)	2.3 (-2.8, 7.3)	0.6677	-8.4 (-61.5, 44.7)	0.8470	0.8950	4.3 (-0.6, 9.1)	0.2339	-10.9 (-46.1, 24.3)	0.6277	0.9524
Large HDL, nmol/L (n=1,653)	0.6 (-2.3, 3.5)	0.8470	-15.0 (-58.1, 28.1)	0.6923	0.8126	-0.4 (-3.2, 2.3)	0.8637	-17.8 (-45.7, 10.2)	0.3821	0.1911
Medium HDL, nmol/L (n=1,241)	7.7 (-1.2, 16.5)	0.2936	-102.0 (-229.7, 25.8)	0.3317	0.1263	6.7 (-1.7, 15.2)	0.2858	-79.1 (-187.2, 29.1)	0.2982	0.2351
Small HDL, nmol/L (n=1,653)	1.1 (-0.3, 2.4)	0.3481	-0.7 (-17.0, 15.5)	0.9663	0.8577	1.1 (-0.1, 2.4)	0.2339	2.6 (-8.2, 13.4)	0.7158	0.9746
Total size of all HDL, nm (n=1,653)	0.0006(-0.3, 0.3)	0.9973	-0.6 (-3.9, 2.7)	0.8470	0.9532	-0.1 (-0.4, 0.2)	0.5244	-0.7 (-2.9, 1.5)	0.6217	0.7205

[a]Individuls were divided into 2 subgroups based on the statin use status: non-users and regular users.

[b]Values presented are percent differences per 1 standard deviation increment in dietary index score obtained from multivariable-adjusted linear regression analyses and the bolded numbers represent statistically significant findings (i.e., FDR p value <0.10). Values are the beta coefficients.

[c]Models were adjusted for total energy intake, age, BMI-continuous, total recreational physical activity, pack-years of smoking; number of supplements used; fasting status at blood draw, race/ethnic groups, educational levels, regular use of NSAID, unopposed estrogen and/or estrogen plus progesterone hormones, hormone therapy (HT) study arms.

[d]Abbreviations: HOMA-IR, Homeostatic model assessment of insulin resistance; HOMA β, Homeostatic model assessment of β-cell function; IGF-1, Insulin-like growth factor-1; IGF-BP1/3/4, Insulin-like growth factor-binding protein 1/3/4; TNF, tumor necrosis factor; VEGF, Vascular endothelial growth factor; VCAM-1, vascular cell adhesion protein 1; ICAM-1, intercellular adhesion molecule 1; GCSF, Granulocyte colony-stimulating factor

Supplemental Table 9. Associations of EDIH and EDIP with biomarkers in categories of regular NSAID use

	**Empirical Dietary Index for Hyperinsulinemia (EDIH) score**					**Empirical Dietary Inflammatory Index (EDIP) score**				
	NSAID non-users[a]		NSAID regular users[a]			NSAID non-users		NSAID regular users		
Biomarkers	Percentage Difference[b,c]	FDR p value	Percentage Difference	FDR p value	FDR P-interaction	Percentage Difference	FDR p value	Percentage Difference	FDR p value	FDR P-interaction
**Insulin and IGF pathway biomarkers**										
Glucose, mg/dL (n=21,669)	**1.2 (1.0, 1.5)**	**2.48E-23**	**1.6 (1.0, 2.2)**	**7.49E-07**	0.9755	**0.8 (0.5, 1.0)**	**2.27E-09**	**1.2 (0.6, 1.8)**	**0.0002**	0.9532
Insulin, ng/mL (n=23,756)	**7.3 (6.4, 8.2)**	**2.70E-29**	**8.2 (5.9, 10.6)**	**9.17E-11**	0.9874	**6.7 (5.9, 7.7)**	**4.00E-29**	**7.2 (4.9, 9.5)**	**9.05E-09**	0.8511
C-peptide, ng/mL (n=943)	**3.9 (0.6, 7.1)**	**0.0498**	9.7 (-3.5, 22.8)	0.2626	0.6083	2.3 (-0.9, 5.5)	0.2696	0.02 (-12.0, 12.1)	0.9973	0.9532
HOMA-IR[d] (n=19,865)	**8.1 (7.1, 9.1)**	**2.70E-29**	**10.4 (7.8, 12.9)**	**2.16E-14**	0.8710	**6.7 (5.7, 7.6)**	**4.00E-29**	**8.1 (5.7, 10.6)**	**1.90E-09**	0.9510
HOMA-B[d] (n=19865)	**3.5 (2.6, 4.4)**	**2.49E-13**	**4.2 (1.9, 6.5)**	**0.0022**	0.9755	**3.9 (3.0, 4.8)**	**7.32E-17**	**3.3 (1.0, 5.5)**	**0.0150**	0.6015
IGF[d]-1, pg/mL (n=3,126)	-0.8 (-4.0, 2.3)	0.7472	0.6 (-7.9, 9.1)	0.9214	0.9524	-2.0 (-5.0, 0.9)	0.2903	-0.5 (-8.8, 7.9)	0.9734	0.8240
IGFBP[d]1, pg/L (n= 993)	**-9.5 (-14.9, -4.2)**	**0.0029**	-7.3 (-22.8, 8.2)	0.4832	0.9532	**-8.0 (-13.5, -2.5)**	**0.0163**	3.0 (-13.7, 19.7)	0.8751	0.5514
IGFBP3, ng/mL (n=2,349)	-0.4 (-2.4, 1.5)	0.7953	-2.8 (-8.2, 2.6)	0.4430	0.8543	0.8 (-1.1, 2.7)	0.5413	-0.1 (-5.4, 5.1)	0.9825	0.9935
IGFBP4, ng/mL (n=354)	3.2 (-1.8, 8.2)	0.3485	5.9 (-5.5, 17.2)	0.4430	0.7899	4.3 (-1.0, 9.6)	0.2181	0.8 (-10.5, 12.1)	0.9734	0.7913
free IGF-1, pg/mL (n=2,203)	-0.4 (-4.7, 3.9)	0.9011	**15.6 (4.8, 26.4)**	**0.0176**	0.2735	-2.5 (-6.6, 1.5)	0.3354	7.0 (-3.5, 17.5)	0.3083	0.5465
**Inflammation and epithelial dysfunction biomarkers**										
C-reactive protein, mg/L (n=26,482)	**7.7 (6.5, 9.0)**	**2.70E-29**	**7.7 (4.6, 10.8)**	**7.26E-06**	0.9532	**6.3 (5.1, 7.5)**	**1.49E-23**	**5.3 (2.3, 8.2)**	**0.0023**	0.8088
Serum Amyloid A, mg/L (n=1,181)	5.0 (-0.3, 10.2)	0.1368	0.4 (-14.3, 15.1)	0.9557	0.7221	3.7 (-1.1, 8.4)	0.2445	1.6 (-12.2, 15.4)	0.9462	0.9532
Interleukin-6, pg/mL (n=12,408)	**6.2 (4.3, 8.1)**	**6.44E-10**	**6.1 (1.0, 11.2)**	**0.0498**	0.7913	**4.2 (2.4, 6.0)**	**2.81E-05**	**8.4 (3.5, 13.3)**	**0.0031**	0.8230
Interleukin 10, pg/L (n=2,466)	-4.0 (-8.1, 0.1)	0.1216	-5.6 (-14.7, 3.4)	0.3515	0.9627	-3.8 (-7.7, 0.2)	0.1545	-4.3 (-12.0, 3.4)	0.3951	0.9548
TNF[d] alpha, mg/mL (n=5,302)	**6.4 (2.3, 10.5)**	**0.0101**	**6.3 (-5.0, 17.6)**	**0.4214**	0.6934	1.5 (-2.4, 5.5)	0.5896	**15.3 (5.3, 25.3)**	**0.0103**	0.5630
TNF alpha receptor 1, pg/mL (n=3,908)	-0.2 (-1.3, 0.9)	0.8449	**3.1 (0.1, 6.1)**	**0.1006**	0.3315	-0.1 (-1.2, 0.9)	0.9462	0.1 (-2.8, 3.1)	0.9788	0.8438
TNF alpha receptor 2, pg/mL (n=7,746)	0.1 (-0.9, 1.1)	0.8926	**2.5 (0.1, 4.9)**	**0.1006**	0.6083	-0.1 (-1.0, 0.9)	0.9734	1.4 (-1.0, 3.7)	0.3748	0.6210
Adiponectin, ng/mL (n=7552)	**-3.7 (-5.7, -1.7)**	**0.0015**	-2.5 (-7.6, 2.6)	0.4655	0.5027	**-6.0 (-7.9, -4.2)**	**4.21E-09**	**-7.9 (-12.8, -3.0)**	**0.0062**	0.9532
Leptin, ng/mL (n=8,045)	**6.7 (4.9, 8.4)**	**7.45E-13**	**6.5 (2.1, 11.0)**	**0.0157**	0.6083	**4.4 (2.8, 6.1)**	**5.54E-07**	**5.7 (1.6, 9.8)**	**0.0237**	0.8543
VEGF[d], pg.ml (n=873)	0.9 (-5.1, 7.0)	0.8449	-8.0 (-20.9, 5.0)	0.3558	0.7030	0.9 (-4.8, 6.6)	0.8973	-0.4 (-11.1, 10.2)	0.9788	0.9964

E-selectin, ng/ml (n=3,817)	**2.4 (0.6, 4.3)**	**0.0301**	**6.5 (2.1, 11.0)**	**0.0157**	0.8126	**3.5 (1.7, 5.2)**	**6.28E-04**	**8.0 (3.4, 12.6)**	**0.0031**	0.8126
VCAM-1[d], ng.ml (n=4,050)	-0.2 (-1.4, 1.0)	0.8317	**4.1 (0.9, 7.2)**	**0.0336**	0.2770	0.8 (-0.4, 1.9)	0.3153	1.4 (-1.6, 4.3)	0.4946	0.8543
ICAM-1[d], ng/ml (n=1,075)	**4.1 (1.1, 7.1)**	**0.0253**	**11.8 (4.6, 19.0)**	**0.0076**	0.8230	2.4 (-0.4, 5.2)	0.2085	5.4 (-0.2, 11.1)	0.1545	0.8543
GCSF[d], pg/ml (n=2,160)	1.1 (-3.2, 5.3)	0.7583	5.8 (-3.0, 14.7)	0.3264	0.7899	-1.0 (-5.1, 3.1)	0.7820	7.7 (0.2, 15.1)	0.1342	0.6083
**Lipid and lipid particle biomarkers**										
Total cholesterol, mg/dL (n=21,378)	0.2 (-0.04, 0.5)	0.1922	0.4 (-0.3, 1.0)	0.4430	0.8329	0.02 (-0.3, 0.3)	0.9734	-0.01 (-0.6, 0.6)	0.9825	0.9874
Triglycerides, mg/dL (n=18,833)	0.6 (-0.2, 1.3)	0.2626	2.0 (-0.04, 4.0)	0.1216	0.8543	**1.6 (0.8, 2.3)**	**0.0003**	1.1 (-0.8, 3.0)	0.3795	0.9176
High density Lipoprotein (HDL), mg/dL (n=20,508)	**-0.5 (-0.9, -0.1)**	**0.0336**	-0.4 (-1.4, 0.6)	0.5886	0.8126	**-1.1 (-1.5, -0.7)**	**7.38E-08**	-0.9 (-1.9, 0.002)	0.1441	0.8438
Low density Lipoprotein (LDL), mg/dL (n=16,525)	**0.8 (0.3, 1.2)**	**0.0052**	0.2 (-0.9, 1.3)	0.8340	0.9233	0.3 (-0.2, 0.8)	0.3150	0.6 (-0.5, 1.7)	0.4041	0.8691
Triglycerides/High density Lipoprotein (n=17,761)	**1.3 (0.3, 2.4)**	**0.0353**	2.4 (-0.3, 5.2)	0.1727	0.9874	**2.7 (1.7, 3.7)**	**2.98E-06**	2.0 (-0.6, 4.6)	0.2445	0.9154
Triglycerides/Total cholesterol (n=18,631)	0.3 (-0.5, 1.0)	0.5988	1.7 (-0.2, 3.6)	0.1609	0.8543	**1.5 (0.8, 2.2)**	**0.0003**	1.1 (-0.7, 2.9)	0.3728	0.9160
Large LDL, nmol/L (n=1,653)	0.5 (-2.0, 3.0)	0.8032	**-10.3 (-16.9, -3.7)**	**0.0104**	0.2735	-2.0 (-4.4, 0.3)	0.2085	-5.7 (-11.8, 0.5)	0.1759	0.9261
Medium LDL, nmol/L (n=1,356)	-0.1 (-3.7, 3.5)	0.9557	**13.0 (3.0, 22.69)**	**0.0336**	0.2072	2.8 (-0.6, 6.2)	0.2122	9.5 (0.2, 18.8)	0.1356	0.5790
Small LDL, nmol/L (n=1,653)	-0.5 (-4.0, 2.9)	0.8449	**10.8 (1.1, 20.6)**	**0.0748**	0.4125	1.2 (-2.1, 4.4)	0.6071	6.9 (-2.1, 15.8)	0.2445	0.7345
Very small LDL, nmol/L (n=1,356)	0.9 (-2.6, 4.4)	0.7583	**11.1 (1.1, 21.1)**	**0.0748**	0.4036	2.8 (-0.5, 6.1)	0.2085	8.4 (-0.8, 17.7)	0.1801	0.6083
Total size of all LDL, nm (n=1,652)	-0.1 (-0.3, 0.1)	0.6464	**-0.9 (-1.4, -0.4)**	**0.0040**	0.2193	-0.2 (-0.4, 0.005)	0.1545	**-0.6 (-1.1, -0.1)**	**0.0467**	0.6934
Intermediate density lipoprotein, nmol/L (n=1,653)	2.7 (-2.7, 8.1)	0.4577	1.9 (-12.9, 16.7)	0.8630	0.8873	4.5 (-0.6, 9.6)	0.1900	6.5 (-7.0, 20.0)	0.4723	0.9532
Large HDL, nmol/L (n=1,653)	2.1 (-0.9, 5.2)	0.2953	**-9.4 (-18.5, -0.4)**	**0.0999**	0.1713	-0.2 (-3.0, 2.7)	0.9734	-6.2 (-14.4, 2.1)	0.2575	0.7021
Medium HDL, nmol/L (n=1,241)	6.3 (-3.1, 15.6)	0.3212	10.2 (-17.2, 37.5)	0.5988	0.7437	6.5 (-2.5, 15.4)	0.2696	6.0 (-18.4, 30.5)	0.7820	0.9573
Small HDL, nmol/L (n=1,653)	1.2 (-0.2, 2.6)	0.1885	0.2 (-4.1, 4.5)	0.9453	0.5394	1.1 (-0.2, 2.5)	0.2085	0.2 (-3.7, 4.2)	0.9734	0.8126
Total size of all HDL, nm (n=1,653)	0.1 (-0.2, 0.5)	0.5988	-0.8 (-1.7, 0.2)	0.2044	0.4036	-0.1 (-0.4, 0.2)	0.7363	-0.7 (-1.6, 0.2)	0.2122	0.4526

[a]Individuls were divided into 2 subgroups based on the NSAID use status: non-users and ever uses.
[b]Values presented are percent differences per 1 standard deviation increment in dietary index score obtained from multivariable-adjusted linear regression analyses and the bolded numbers represent statistically significant findings (i.e., FDR p value <0.10). Values are the beta coefficients.
[c]Models were adjusted for total energy intake, age, BMI-continuous, total recreational physical activity, pack-years of smoking, number of supplements used, fasting status at blood draw, race/ethnic groups, educational levels, regular use of statins, unopposed estrogen and/or estrogen plus progesterone hormones, hormone therapy (HT) study arms.
[d]Abbreviations: NSAID, Non-steroidal anti-inflammatory drugs; HOMA-IR, Homeostatic model assessment of insulin resistance; HOMA β, Homeostatic model assessment of β-cell function; IGF-1, Insulin-like growth factor-1; IGF-BP1/3/4, Insulin-like growth factor-binding protein 1/3/4; TNF, tumor necrosis factor; VEGF, Vascular endothelial growth factor; VCAM-1, vascular cell adhesion protein 1; ICAM-1, intercellular adhesion molecule 1; GCSF, Granulocyte colony-stimulating factor

Supplemental Table 10. Associations of EDIH and EDIP with biomarkers in categories of unopposed estrogen use

Biomarkers	Empirical Dietary Index for Hyperinsulinemia (EDIH) score							Empirical Dietary Inflammatory Index (EDIP) score						
	Never users[a]		Past users[a]		Current users[a]		FDR P-interaction	Never users		Past users		Current users		FDR P-interaction
	Percentage Difference[b,c]	FDR p value	Percentage Difference	FDR p value	Percentage Difference	FDR p value		Percentage Difference	FDR p value	Percentage Difference	FDR p value	Percentage Difference	FDR p value	
**Insulin and IGF pathway biomarkers**														
Glucose, mg/dL (n=21,669)	**1.3 (1.0, 1.5)**	**6.55E-19**	**1.6 (1.0, 2.1)**	**1.05E-06**	**1.2 (0.7, 1.7)**	**8.93E-06**	0.7345	**0.9 (0.6, 1.1)**	**6.04E-10**	**0.8 (0.2, 1.4)**	**0.0246**	**0.7 (0.3, 1.2)**	**0.0120**	0.8543
Insulin, ng/mL (n=23,756)	**7.2 (6.2, 8.2)**	**6.00E-29**	**8.5 (6.3, 10.7)**	**1.18E-12**	**7.7 (5.4, 9.9)**	**3.93E-10**	0.8353	**6.3 (5.3, 7.3)**	**6.00E-29**	**6.5 (4.3, 8.7)**	**1.38E-07**	**9.5 (7.3, 11.7)**	**2.88E-15**	0.2735
C-peptide, ng/mL (n=943)	4.0 (-0.03, 8.0)	0.1755	8.8 (-4.2, 21.8)	0.3788	5.1 (-1.3, 11.5)	0.2724	0.9624	2.0 (-1.9, 5.9)	0.5190	-9.2 (-18.9, 0.5)	0.1815	**7.8 (1.1, 14.5)**	**0.0795**	0.5176
HOMA-IR[d] (n=19,865)	**8.2 (7.1, 9.3)**	**6.00E-29**	**9.7 (7.3, 12.1)**	**6.81E-14**	**8.3 (6.0, 10.6)**	**5.22E-11**	0.7913	**6.7 (5.6, 7.8)**	**6.00E-29**	**6.8 (4.4, 9.3)**	**3.52E-07**	**8.4 (6.1, 10.8)**	**4.64E-11**	0.8691
HOMA-B [d] (n=19865)	**3.6 (2.6, 4.6)**	**1.68E-11**	**4.1 (1.9, 6.3)**	**0.0015**	**3.1 (1.0, 5.2)**	**0.0210**	0.9573	**3.5 (2.6, 4.5)**	**3.19E-11**	**4.4 (2.3, 6.6)**	**0.0004**	**4.7 (2.6, 6.8)**	**0.0001**	0.8240
IGF[d]-1, pg/mL (n=3,126)	1.0 (-4.4, 2.4)	0.7291	-0.1 (-7.6, 7.3)	0.9925	2.3 (-5.6, 10.1)	0.7291	0.8950	-1.4 (-4.8, 1.9)	0.5923	-3.6 (-10.3, 3.2)	0.5118	-1.9 (-9.0, 5.2)	0.7680	0.8691
IGFBP[d]1, pg/L (n= 993)	**-11.8 (-18.2, -5.3)**	**0.0024**	-1.6 (-19.6, 16.5)	0.9506	-3.6 (-12.4, 5.3)	0.6149	0.8230	**-12.8 (-19.5, -6.1)**	**0.0012**	5.8 (-11.5, 23.1)	0.6820	-4.5 (-13.7, 4.7)	0.5313	0.1617
IGFBP3, ng/mL (n=2,349)	-1.6 (-3.8, 0.7)	0.3627	4.4 (-1.1, 10.0)	0.2724	-0.6 (-4.2, 2.9)	0.8547	0.6273	-0.4 (-2.7, 1.8)	0.8414	2.9 (-2.0, 7.9)	0.4564	2.2 (-1.0, 5.3)	0.3647	0.7437
IGFBP4, ng/mL (n=354)	3.8 (-1.8, 9.4)	0.3788	2.9 (-6.9, 12.6)	0.7291	-	-	0.9782	6.1 (0.5, 11.7)	0.1165	-9.0 (-20.7, 2.7)	0.2956	-	-	0.2648
free IGF-1, pg/mL (n=2,203)	4.7 (-0.0004, 9.4)	0.1751	4.8 (-6.2, 15.8)	0.5918	**-19.6 (-32.1, -7.1)**	**0.0127**	0.1486	0.8 (-3.7, 5.3)	0.8608	-4.3 (-14.8, 6.2)	0.6074	**-13.4 (-24.1, -2.7)**	**0.0520**	0.3923
**Inflammation and epithelial dysfunction biomarkers**														
C-reactive protein, mg/L (n=26,482)	**7.4 (6.1, 8.8)**	**2.14E-24**	**9.0 (6.0, 12.1)**	**6.18E-08**	**4.9 (2.3, 7.6)**	**0.0018**	**0.0498**	**5.8 (4.5, 7.2)**	**7.89E-16**	**7.5 (4.6, 10.5)**	**6.34E-06**	**3.8 (1.3, 6.4)**	**0.0137**	0.4988
Serum Amyloid A, mg/L (n=1,181)	4.0 (-2.1, 10.1)	0.3978	1.4 (-15.0, 17.8)	0.9506	-1.6 (-12.1, 9.0)	0.8881	0.7345	5.1 (-0.6, 10.7)	0.2079	5.0 (-8.7, 18.7)	0.6571	-4.8 (-14.6, 5.0)	0.5313	0.4473
Interleukin-6, pg/mL (n=12,408)	**6.5 (4.4, 8.7)**	**1.89E-08**	**5.7 (0.8, 10.7)**	**0.0977**	**5.9 (2.0, 9.9)**	**0.0189**	0.9468	**5.0 (3.0, 7.1)**	**1.45E-05**	**6.0 (1.3, 10.7)**	**0.0465**	3.8 (0.01, 7.6)	0.1469	0.7913
Interleukin 10, pg/L (n=2,466)	-2.3 (-6.9, 2.3)	0.5479	-2.7 (-14.0, 8.7)	0.7752	**-10.5 (-19.1, -1.8)**	**0.0783**	0.8230	-4.3 (-8.6, -0.1)	0.1413	-3.3 (-14.0, 7.5)	0.7271	-1.8 (-9.6, 6.0)	0.7875	0.8438
TNF[d] alpha, mg/mL (n=5,302)	4.3 (-0.4, 8.9)	0.2154	9.0 (-2.3, 20.3)	0.2724	**12.4 (3.8, 20.9)**	**0.0223**	0.6934	3.3 (-1.1, 7.8)	0.3173	7.7 (-3.2, 18.5)	0.3470	1.9 (-6.1, 9.9)	0.7787	0.8353
TNF alpha receptor 1, pg/mL (n=3,908)	-0.001 (-1.2, 1.2)	0.9988	-0.2 (-2.6, 2.3)	0.9506	1.7 (-1.7, 5.1)	0.5457	0.8543	-0.6 (-1.7, 0.5)	0.5118	-0.7 (-2.9, 1.6)	0.7401	2.6 (-0.3, 5.6)	0.2079	0.4869
TNF alpha receptor 2, pg/mL (n=7,746)	0.3 (-0.8, 1.4)	0.7553	0.8 (-1.9, 3.5)	0.7291	0.2 (-1.9, 2.3)	0.9506	0.9612	0.2 (-0.9, 1.2)	0.8608	-0.1 (-2.6, 2.4)	0.9781	-0.1 (-2.1, 1.9)	0.9692	0.9874

Adiponectin, ng/mL (n=7552)	**-4.4 (-6.7, -2.2)**	**0.0008**	-1.4 (-7.2, 4.3)	0.7654	-2.3 (-6.2, 1.6)	0.4668	0.9176	**-5.1 (-7.3, -2.9)**	**3.69E-05**	**-8.0 (-13.2, -2.8)**	**0.0120**	**-9.1 (-12.7, -5.4)**	**1.17E-05**	0.4263
Leptin, ng/mL (n=8,045)	**6.3 (4.3, 8.3)**	**6.82E-09**	4.0 (-0.8, 8.8)	0.2372	**8.2 (4.8, 11.7)**	**2.09E-05**	0.9160	**3.7 (1.9, 5.6)**	**6.00E-04**	3.8 (-0.5, 8.1)	0.2238	**6.8 (3.6, 10.0)**	**0.0002**	0.7437
VEGF[d], pg.ml (n=873)	-2.0 (-8.0, 4.1)	0.7021	-13.7 (-42.6, 15.3)	0.5642	5.1 (-8.1, 18.4)	0.6311	0.8240	-2.8 (-8.7, 3.2)	0.5580	9.6 (-13.3, 32.5)	0.5923	5.2 (-6.2, 16.6)	0.5633	0.8088
E-selectin, ng/ml (n=3,817)	**2.9 (0.9, 5.0)**	**0.0264**	2.0 (-2.4, 6.5)	0.5796	3.6 (-0.5, 7.7)	0.2194	0.9755	**3.4 (1.4, 5.4)**	**0.0050**	3.3 (-1.1, 7.7)	0.3173	**6.8 (2.7, 10.9)**	**0.0052**	0.8401
VCAM-1[d], ng.ml (n=4,050)	0.1 (-1.3, 1.5)	0.9506	3.5 (-0.05, 7.1)	0.1758	-0.8 (-3.2, 1.5)	0.6772	0.7345	1.1 (-0.2, 2.4)	0.2494	1.8 (-1.6, 5.1)	0.5118	-0.3 (-2.7, 2.0)	0.8608	0.9612
ICAM-1[d], ng/ml (n=1,075)	2.5 (-0.7, 5.7)	0.2932	11.3 (0.6, 22.0)	0.1422	**10.9 (4.3, 17.4)**	**0.0073**	0.1713	2.3 (-0.7, 5.2)	0.2956	8.9 (-0.7, 18.5)	0.1938	1.6 (-4.7, 7.9)	0.7745	0.8543
GCSF[d], pg/ml (n=2,160)	1.2 (-3.8, 6.2)	0.7654	0.4 (-10.4, 11.3)	0.9676	7.1 (-1.1, 15.2)	0.2264	0.6871	1.3 (-3.4, 6.0)	0.7512	-0.8 (-11.9, 10.3)	0.9481	0.5 (-6.8, 7.9)	0.9481	0.8543
**Lipid and lipid particle biomarkers**														
Total cholesterol, mg/dL (n=21,378)	0.3 (-0.3, 0.6)	0.2074	0.3 (-0.4, 0.9)	0.5929	0.04 (-0.6, 0.7)	0.9506	0.9640	0.02 (-0.3, 0.3)	0.9526	0.06 (-0.6, 0.6)	0.9253	-0.01 (-0.6, 0.6)	0.9903	0.9108
Triglycerides, mg/dL (n=18,833)	0.8 (-0.1, 1.6)	0.2133	0.3 (-1.5, 2.1)	0.8727	-0.03 (-2.0, 1.9)	0.9925	0.8543	**1.6 (0.7, 2.4)**	**0.0013**	6.2 (-1.2, 2.4)	0.6820	1.0 (-1.0, 2.9)	0.5311	0.9160
High density Lipoprotein (HDL), mg/dL (n=20,508)	-0.4 (-0.8, 0.02)	0.2054	-0.9 (-0.8, 0.1)	0.2057	-0.8 (-1.8, 0.1)	0.2194	0.9160	**-1.0 (-1.4, -0.6)**	**2.70E-05**	**-1.4 (-2.3, -0.5)**	**0.0118**	**-1.6 (-2.6, -0.7)**	**0.0036**	0.7717
Low density Lipoprotein (LDL), mg/dL (n=16,525)	**0.7 (0.2, 1.2)**	**0.0211**	0.9 (-0.1, 1.9)	0.2194	0.7 (-0.6, 2.0)	0.4778	0.8207	0.3 (-0.2, 0.8)	0.4182	0.9 (-0.2, 1.9)	0.2403	0.6 (-0.7, 1.9)	0.5633	0.7462
Triglycerides/High density Lipoprotein (n=17,761)	1.3 (0.1, 2.5)	0.1129	1.6 (-0.9, 4.2)	0.3978	1.4 (-1.2, 4.1)	0.4888	0.9233	**2.5 (1.3, 3.6)**	**0.0002**	2.1 (-0.4, 4.6)	0.2494	**3.4 (0.8, 6.1)**	**0.0401**	0.8438
Triglycerides/Total cholesterol (n=18,631)	0.4 (-0.4, 1.2)	0.5488	0.1 (-1.6, 1.8)	0.9566	-0.01 (-1.9, 1.9)	0.9988	0.8438	**1.4 (0.6, 2.2)**	**0.0020**	0.6 (-1.1, 2.3)	0.6744	1.1 (-0.8, 2.9)	0.4564	0.8873
Large LDL, nmol/L (n=1,653)	-2.1 (-4.9, 0.8)	0.3324	-0.4 (-6.6, 5.8)	0.9506	3.5 (-2.1, 9.1)	0.4365	0.6273	-0.2 (-4.9, 0.4)	0.2399	-3.5 (-9.5, 2.5)	0.4564	-2.2 (-7.9, 3.4)	0.6182	0.8691
Medium LDL, nmol/L (n=1,356)	3.0 (-1.0, 7.1)	0.3187	2.0 (-6.7, 10.7)	0.7759	-5.4 (-14.9, 4.1)	0.4793	0.6934	3.7 (-0.08, 7.5)	0.1602	1.7 (-6.7, 10.0)	0.8284	4.6 (-4.5, 13.8)	0.5295	0.9530
Small LDL, nmol/L (n=1,653)	2.3 (-1.5, 6.2)	0.4432	2.0 (-5.8, 9.9)	0.7654	-4.4 (-13.2, 4.5)	0.5488	0.8126	2.3 (-1.3, 5.8)	0.4141	0.1 (-7.5, 7.7)	0.9892	2.6 (-6.3, 11.5)	0.7330	0.8710
Very small LDL, nmol/L (n=1,356)	3.6 (-0.3, 7.5)	0.2088	3.4 (-5.0, 11.8)	0.6131	-5.2 (-14.6, 4.3)	0.4907	0.6934	3.8 (0.2, 7.5)	0.1281	1.1 (-6.9, 9.1)	0.8727	4.2 (-4.9, 13.3)	0.5586	0.8873
Total size of all LDL, nm (n=1,652)	-0.2 (-0.4, -0.01)	0.1498	-0.2 (-0.7, 0.3)	0.5918	0.1 (-0.3, 0.5)	0.7654	0.8438	-0.2 (-0.4, -0.005)	0.1407	-0.2 (-0.7, 0.2)	0.5313	-0.3 (-0.7, 0.2)	0.4105	0.9612
Intermediate density lipoprotein, nmol/L (n=1,653)	2.7 (-3.4, 8.8)	0.5918	-0.9 (-14.6, 12.9)	0.9506	1.0 (-10.9, 12.8)	0.9506	0.9930	3.7 (-2.0, 9.3)	0.4105	4.9 (-8.4, 18.1)	0.6571	0.8 (-11.1, 12.7)	0.9526	0.8577
Large HDL, nmol/L (n=1,653)	-1.8 (-5.4, 1.9)	0.5642	-3.4 (-10.8, 4.0)	0.5796	**7.9 (1.9, 13.9)**	**0.0463**	0.3532	-2.0 (-5.4, 1.5)	0.4692	-0.04 (-7.2, 7.1)	0.9920	-1.0 (-7.1, 5.1)	0.8608	0.9532
Medium HDL, nmol/L (n=1,241)	9.2 (-1.5, 20.0)	0.2270	-13.1 (-35.6, 9.4)	0.4668	8.0 (-14.4, 30.5)	0.6747	0.8691	5.6 (-4.4, 15.6)	0.4871	4.5 (-18.4, 27.4)	0.8284	1.9 (-20.5, 24.3)	0.9459	0.9532

Small HDL, nmol/L (n=1,653)	0.5 (-1.1, 2.1)	0.7291	1.1 (-2.2, 4.4)	0.7012	3.9 (0.3, 7.5)	0.1341	0.7651	1.1 (-0.4, 2.5)	0.3385	-0.8 (-4.0, 2.4)	0.7680	3.8 (0.2, 7.5)	0.1223	0.6083
Total size of all HDL, nm (n=1,653)	-0.04 (-0.4, 0.3)	0.9506	-0.7 (-1.4, 0.08)	0.2188	0.3 (-0.4, 1.1)	0.5938	0.7651	-0.3 (-0.6, 0.1)	0.3196	-0.03 (-0.8, 0.7)	0.9692	-0.1 (-0.9, 0.7)	0.8608	0.9603

[a]Individuls were divided into 3 subgroups based on the unopposed estrogen usage status: never users, past users and current users.

[b]Values presented are percent differences per 1 standard deviation increment in dietary index score obtained from multivariable-adjusted linear regression analyses and the bolded numbers represent statistically significant findings (i.e., FDR p value <0.05). Values are the beta coefficients.

cModels were adjusted for total energy intake, age, BMI-continuous, total recreational physical activity, pack-years of smoking, number of supplements used, fasting status at blood draw, race/ethnic groups, educational levels, regular use of NSAID, statins, hormone therapy (HT) study arms.

[d]Abbreviations: NSAID, Non-steroidal anti-inflammatory drugs; HOMA-IR, Homeostatic model assessment of insulin resistance; HOMA β, Homeostatic model assessment of β-cell function; IGF-1, Insulin-like growth factor-1; IGF-BP1/3/4, Insulin-like growth factor-binding protein 1/3/4; TNF, tumor necrosis factor; VEGF, Vascular endothelial growth factor; VCAM-1, vascular cell adhesion protein 1; ICAM-1, intercellular adhesion molecule 1; GCSF, Granulocyte colony-stimulating factor

List of Women's Health Initiative Investigators

Program Office: (National Heart, Lung, and Blood Institute, Bethesda, Maryland) Jacques Rossouw, Shari Ludlam, Joan McGowan, Leslie Ford, and Nancy Geller

Clinical Coordinating Center: (Fred Hutchinson Cancer Research Center, Seattle, WA) Garnet Anderson, Ross Prentice, Andrea LaCroix, and Charles Kooperberg

Investigators and Academic Centers: (Brigham and Women's Hospital, Harvard Medical School, Boston, MA) JoAnn E. Manson; (MedStar Health Research Institute/Howard University, Washington, DC) Barbara V. Howard; (Stanford Prevention Research Center, Stanford, CA) Marcia L. Stefanick; (The Ohio State University, Columbus, OH) Rebecca Jackson; (University of Arizona, Tucson/Phoenix, AZ) Cynthia A. Thomson; (University at Buffalo, Buffalo, NY) Jean Wactawski-Wende; (University of Florida, Gainesville/Jacksonville, FL) Marian Limacher; (University of Iowa, Iowa City/Davenport, IA) Jennifer Robinson; (University of Pittsburgh, Pittsburgh, PA) Lewis Kuller; (Wake Forest University School of Medicine, Winston-Salem, NC) Sally Shumaker; (University of Nevada, Reno, NV) Robert Brunner

Women's Health Initiative Memory Study: (Wake Forest University School of Medicine, Winston-Salem, NC) Mark Espeland

For a list of all the investigators who have contributed to WHI science, please visit: https://www.whi.org/researchers/Documents%20%20Write%20a%20Paper/WHI%20Investigator %20Long%20List.pdf
